# Transformation of Silyl‐Protected Tetrafluorinated Thia[6]helicene S‐Oxide into a Difluorinated Coronene via Induced Desilylation

**DOI:** 10.1002/chem.202502242

**Published:** 2025-08-25

**Authors:** Ayumu Nakao, Hiroshi Katagiri, Takashi Murase

**Affiliations:** ^1^ Faculty of Science Yamagata University 1‐4‐12 Kojirakawa‐machi Yamagata 990‐8560 Japan; ^2^ Graduate School of Organic Materials Science Yamagata University 4‐3‐16 Jonan Yonezawa 992‐8510 Japan

**Keywords:** coronene, desilylation, fluorine, helicenes, thiophene

## Abstract

Sulfur oxidation of thiophene rings is an effective strategy for constructing substituted and polycyclic arenes via Diels–Alder reactions, wherein each thiophene ring is converted into a benzene ring. In the context of converting helicenes into planar coronenes, tetrafluorinated thia[6]helicene *S*,*S*‐dioxide undergoes a smooth intramolecular Diels–Alder reaction to afford the difluorinated coronene. However, the corresponding *S*‐oxide counterpart is affected by competing side reactions that limit its utility. Herein, we demonstrate that introducing a trimethylsilyl (TMS) group onto the thiophene *S*‐oxide ring suppresses self‐condensation and enables more efficient coronene conversion than its *S*,*S*‐dioxide counterpart. Unexpectedly, the TMS group is removed during the transformation, yielding both the TMS‐protected and deprotected forms of the difluorinated coronene. Mechanistic investigations suggest that this desilylation arises from the combined effect of Si···F interactions and in‐situ‐generated sulfur monoxide (SO). These findings provide insights into the reactivity control of thiophene *S*‐oxides and demonstrate how silicon, fluorine, and SO can influence cascade transformations toward functionalized polycyclic aromatic systems.

## Graphical abstract

Trimethylsilyl‐protected tetrafluorinated thia[6]helicene *S*‐oxide undergoes a more efficient transformation to difluorinated coronene than its unprotected *S*‐oxide and *S*,*S*‐dioxide counterparts. Si···F interactions and in‐situ‐generated sulfur monoxide facilitate selective desilylation, offering mechanistic insight into this cascade transformation.

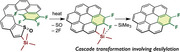

## 1 Introduction

Helicenes, with their screw‐shaped structures, exemplify a well‐known structural contradiction: while polycyclic aromatic systems are typically expected to be planar because of their conjugation, helicenes instead form nonplanar, fully aromatic helices.^[^ ^1^ ^]^ This unique geometry not only exhibits inherent chirality but also imparts helicenes with intramolecular Diels–Alder reactivity.^[^ ^2^, ^3^ ^]^ An overlooked aspect of helicene chemistry is their ability to transform into planar structures under specific external stimuli, even without the addition of reagents. For example, [5]helicene can be converted into benzo[*ghi*]perylene through photochemical cyclodehydrogenation,^[^ ^4^ ^]^ or even thermal cyclodehydrogenation using flash vacuum pyrolysis.^[^ ^5^ ^]^ Remarkably, heating [6]helicene to 485 °C in a sealed tube induces its planarization, yielding trace amounts of coronene.^[^ ^6^ ^]^ These transformations illustrate the structural versatility of helicenes, bridging the gap between nonplanar and planar polycyclic aromatic systems. However, their limited efficiency in the case of [6]helicene highlights the challenges that remain in realizing their synthetic potential.

**Scheme 1:**
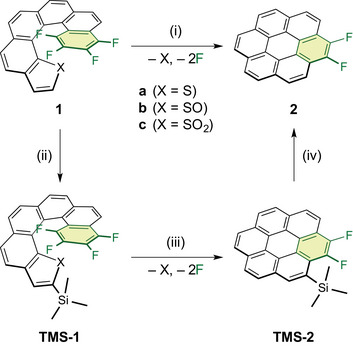
“Helix‐to‐disc” conversion of F_4_‐thia[6]helicene **1** into F_2_‐coronene **2**. (i, iii) A cascade reaction triggered by an intramolecular Diels–Alder reaction, followed by cheletropic extrusion of sulfur‐containing species and elimination of two fluorine atoms, (ii) silylation of the thiophene ring, and (iv) desilylation at the coronene framework. Helicene structures are depicted in the *M* configuration for clarity; however, all samples were used as racemic mixtures.

To address the inefficient transformation of [6]helicene into coronene, we recently demonstrated that replacing one of its terminal benzene rings with a thiophene ring having the sulfur atom oriented inwards enabled its photochemical transformation into coronene.^[^ ^7^ ^]^ The introduction of four fluorine atoms to the opposite terminal benzene ring produced F_4_‐thia[6]helicene **1a**, which underwent facile photochemical transformation into F_2_‐coronene **2** (Scheme 1, route (i)). The corresponding thermal transformation is facilitated by *S*‐oxidation of the thiophene ring to its *S*,*S*‐dioxide form. Specifically, heating F_4_‐thia[6]helicene *S*,*S*‐dioxide **1c** in mesitylene at 160 °C afforded **2** in 55% yield,^[^ ^7^ ^]^ representing a significant improvement in efficiency compared with the reaction of the parent [6]helicene. Although the *S*‐oxide form **1b** also undergoes this “helix‐to‐disc” conversion, its efficiency is reduced by the effect of competing side reactions, including deoxygenation of the thiophene *S*‐oxide ring and self‐condensation of **1b** via a Diels–Alder reaction.

As revealed by our prior DFT analysis,^[^ ^7^ ^]^ the transformation of **1c** into **2** begins with an intramolecular Diels–Alder reaction. The calculations showed that the LUMO is found on the thiophene *S*,*S*‐dioxide ring (diene) and the HOMO on the F_4_‐benzene ring (dienophile), suggesting an inverse electron‐demand nature. Favorable overlap of these frontier orbitals, enforced by the helicene topology, dictates the regioselectivity of the [4+2] cycloaddition. This cycloaddition is followed by spontaneous cheletropic extrusion of sulfur dioxide (SO_2_) and elimination of two fluorine atoms. A similar sequence is proposed for the transformation of **1b** into **2**, except for the extrusion of sulfur monoxide (SO). Although susceptible to side reactions, **1b** was calculated to have a lower activation free energy for the intramolecular Diels–Alder reaction, indicating higher intrinsic reactivity than **1c**.

Given that a thiophene *S*‐oxide ring is generally considered more favorable for Diels–Alder reactions than a thiophene *S*,*S*‐dioxide ring,^[^ ^8^ ^]^ the reduced efficiency of **1b** in its transformation into **2** was unexpected. Indeed, thiophene *S*‐oxides are known to act as useful dienes, affording substituted arenes.^[^ ^9^ ^]^ A striking example of this reactivity is the synthesis of hexaarylbenzenes with six different substituents.^[^ ^10^ ^]^ This transformation was achieved through Diels–Alder reactions between tetraarylthiophene *S*‐oxides and diarylethynes, enabling regioselective aryl group installation on the central benzene core. Notably, the corresponding tetraarylthiophene *S*,*S*‐dioxides did not undergo Diels–Alder reactions with diarylethynes. This result highlights the distinct reactivity of substituted thiophene *S*‐oxides, which are inherently nonplanar in conformation.^[^ ^11^ ^]^ Thus, substituted thiophene *S*‐oxides retain their high reactivity while suppressing self‐condensation, enabling the desired transformations.

Herein, we report trimethylsilyl (TMS)‐protected F_4_‐thia[6]helicene *S*‐oxide **TMS‐1b**, which bears a TMS group at the terminal thiophene *S*‐oxide ring (Scheme 1, route (ii)). Among silyl protecting groups, TMS was selected for its minimal steric hindrance during coronene conversion and facile removal afterward (Scheme 1, routes (iii, iv)). **TMS‐1b** exhibited higher efficiency in coronene conversion than **1c**, despite the steric hindrance introduced by the TMS group, which is absent in **1c**. Furthermore, no self‐condensation was observed. Unexpectedly, TMS cleavage occurred during the transformation into **TMS‐2**, yielding not only **TMS‐2** but also **2**. X‐ray crystallographic analysis of **TMS‐2** provided insight into this desilylation process. The close proximity of the Si and F atoms in **TMS‐2** highlights a key structural feature of this transformation, shedding light on their roles in the reaction mechanism.

## 2 Results and Discussion

### 2.1 Synthesis of TMS‐1

Thiahelicenes have been synthesized via various methods, among which the pioneering work by Wynberg et al. demonstrated the effectiveness of stilbene‐type photocyclization.^[^ ^12^ ^]^ This approach has since been widely utilized, predominantly yielding thiahelicenes with sulfur atoms oriented outwards, with octathia[15]helicene, the longest known thiahelicene, being a representative case.^[^ ^13^ ^]^ However, inward‐oriented thiahelicenes have been less explored, although recent studies by Guijarro et al. have demonstrated their synthesis via photocyclization,^[^ ^14^ ^]^ as exemplified by the preparation of **1a** in this study.

**Scheme 2:**
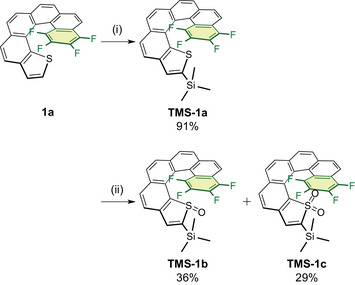
Synthesis of **TMS‐1**. Conditions: (i) LDA, TMS‐Cl, THF, −80 °C, 4 h; (ii) *m*‐CPBA (1.0 equiv.), CH_2_Cl_2_, 0 °C, 5 h.

One advantage of thiahelicenes is their ability to undergo regioselective functionalization at the terminal thiophene ring.^[^ ^15^ ^]^ Such functionalization can be achieved through direct electrophilic substitution or via the generation of α‐anions followed by reaction with electrophiles, enabling precise and versatile modifications. The functionalization of **1a** was carried out using lithium diisopropylamide (LDA), followed by the addition of TMS‐Cl, to afford **TMS‐1a** in 91% yield (Scheme 2). The bulky LDA base was selected instead of the conventional *n*‐BuLi to avoid nucleophilic aromatic substitution at the terminal F_4_‐benzene ring.^[^ ^16^ ^]^

The selective oxidation of a thiophene ring to its *S*‐oxide is typically achieved using *m*‐CPBA in the presence of BF_3_·Et_2_O.^[^ ^17^ ^]^ Using this method, **1b** was prepared from **1a** in 97% yield.^[^ ^7^ ^]^ However, applying this method to **TMS‐1a** resulted in the rapid removal of the TMS group, regenerating **1a**. Therefore, **TMS‐1a** was treated with *m*‐CPBA (1.0 equiv.) in the absence of BF_3_·Et_2_O, affording a mixture of **TMS‐1b** (36%), **TMS‐1c** (29%), and unreacted **TMS‐1a** (29%). The TMS‐functionalized products were readily separated by column chromatography on silica gel with CHCl_3_, eluting in the following order: **TMS‐1a** < **TMS‐1c** < **TMS‐1b**. Notably, **TMS‐1b** exhibited enhanced stability in solution, resisting self‐condensation and deoxygenation for over one month at room temperature (Figure S1). In contrast, **1b** gradually underwent these side reactions under the same conditions, demonstrating the stabilizing effect of the TMS group.

### 2.2 Conversion Into TMS‐2

**Scheme 3:**
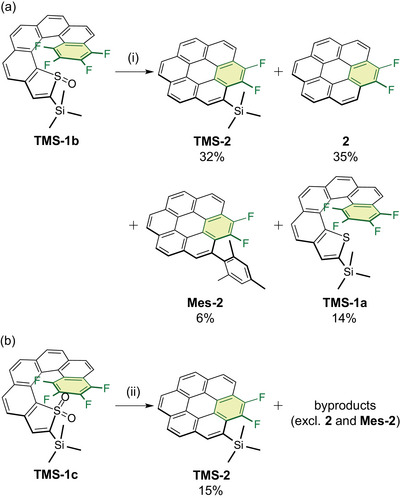
(a,b) Thermal transformation of **TMS‐1b** and **TMS‐1c** into **TMS‐2**. Conditions: (i) mesitylene, 160 °C, 30 min; (ii) mesitylene, 160 °C, 80 min.

With the thermally stable **TMS‐1b** in hand, we next performed its coronene conversion in mesitylene at 160 °C (Scheme 3a). After heating for 30 min, the starting material was completely consumed, yielding **TMS‐2** (32%), **2** (35%), and **Mes‐2** (6%), in which the TMS group was removed and solvent mesitylene was attached onto the coronene core. Additionally, deoxygenation of the thiophene *S*‐oxide ring afforded **TMS‐1a** (14%). Hence, the combined yields of **TMS‐2**, **2**, and **Mes‐2** resulted in an overall coronene conversion of 73%, surpassing the 55% yield obtained from **1c**. In contrast, **TMS‐1c** required prolonged heating (80 min) for complete consumption, yet the coronene conversion yield was significantly lower (15%), accompanied by numerous complex byproducts, excluding **2** and **Mes‐2** (Scheme 3b). These results indicate that the TMS group retarded coronene conversion but preserved the intrinsic high reactivity of the thiophene *S*‐oxide ring. Unlike **2**, which adopts a completely planar structure, **TMS‐2** and **Mes‐2** exhibit enhanced solubility in CH_2_Cl_2_ and CHCl_3_ owing to the steric effects of the TMS and mesityl groups.

**Figure 1:**
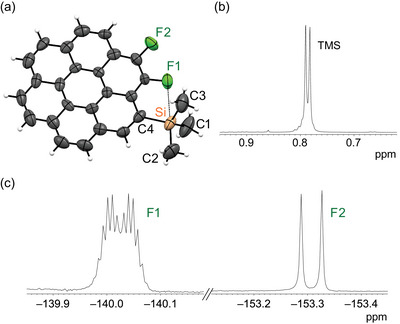
(a) Crystal structure of **TMS‐2**. Thermal displacement ellipsoid plot of **TMS‐2** at the 50% probability level. Selected interatomic distances (Å): C1–Si, 1.843(9); C2–Si, 1.903(8); C3–Si, 1.840(8); C4–Si, 1.895(5); Si···F1, 2.842(4). (b) ^1^H NMR spectrum (500 MHz, 298 K, CDCl_3_) of **TMS‐2** (TMS region only). (c) ^19^F NMR spectrum (470 MHz, 298 K, CDCl_3_) of **TMS‐2**.

The structure of **TMS‐2** was unambiguously determined by X‐ray crystallographic analysis (Figure 1a).^[^ ^18^ ^]^ The Si atom was in close contact with the F1 atom (Si···F1, 2.842 Å), forming a pseudo‐pentacoordinate Si center.^[^ ^19^ ^]^ This distance was considerably shorter than the sum of the van der Waals radii of silicon and fluorine (3.57 Å), suggesting a significant Si···F interaction. Notably, the C2–Si bond (1.903 Å), which was positioned trans to the F1 atom, was longer than the C1–Si bond (1.843 Å) and the C3–Si bond (1.840 Å) in the TMS group. Similarly, the aromatic C4–Si bond (1.895 Å) was elongated and weakened, resembling the C2–Si bond. The close proximity of the F1 atom influenced the NMR spectra of **TMS‐2** (Figure 1b, c). The ^1^H NMR signal of the TMS protons appeared as a doublet at *δ* = 0.79 ppm because of long‐range H–F1 coupling (^6^ *J*_HF_ = 4.1 Hz). The ^19^F NMR signal of the F1 atom was distinctive, appearing as a doublet of decets, with an initial doublet splitting because of vicinal F1–F2 coupling (^3^ *J*_FF_ = 18.2 Hz), followed by further splitting into a decet caused by the nine protons of the TMS group.

**Figure 2:**
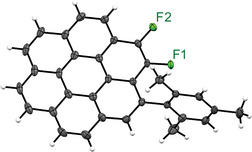
Crystal structure of **Mes‐2**. Thermal displacement ellipsoid plot of **Mes‐2** at the 50% probability level.

The X‐ray crystal structure of **Mes‐2** provided conclusive evidence that solvent mesitylene had indeed bound to the coronene core, confirming the unexpected formation of the arylated product (Figure 2).^[^ ^18^ ^]^ In the ^1^H NMR spectrum of **Mes‐2**, the aromatic protons of the mesityl group appeared at 7.15 ppm and its methyl protons resonated at 2.50 and 2.04 ppm with an intensity ratio of 1:2. Unlike the TMS methyl protons of **TMS‐2**, these singlet signals indicated that the mesityl methyl protons were distant from the fluorine atoms and that the mesityl group underwent restricted rotation around the coronene core on the NMR timescale. In the ^19^F NMR spectrum, the F1 and F2 atoms appeared at −146.5 and −152.2 ppm, respectively, behaving as doublets because only vicinal F1–F2 coupling occurred. This simple splitting pattern for the F1 atom contrasted with the more complex signal observed for F1 in **TMS‐2** (Figure 1c).

First, we hypothesized that the removal of the TMS group during the transformation of **TMS‐1b** into **TMS‐2** at 160 °C occurred spontaneously because of the weakened aromatic C4–Si bond in **TMS‐2**. However, when isolated, **TMS‐2** remained intact even after heating at 160 °C for 2 h, suggesting that thermal cleavage of the C4–Si bond alone was insufficient to account for the TMS group removal. Given that two fluorine atoms were eliminated during the transformation, we next hypothesized that the liberated fluorine species interacted with the Si atom, facilitating the cleavage of the C4–Si bond. Indeed, treatment of **TMS‐2** with Bu_4_NF in THF at room temperature effected facile desilylation within 3 h, affording **2** in quantitative yield (Scheme 1, route (iv)). However, during the transformation of **TMS‐1c** into **TMS‐2**, no desilylation was observed, even though the same elimination of two fluorine atoms occurred (Scheme 3b). Therefore, the liberated fluorine species were not fluoride anions and did not contribute to the cleavage of the C4–Si bond.

**Scheme 4:**
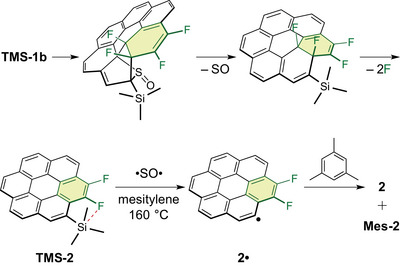
Proposed mechanism for the formation of **2** and **Mes‐2** from **TMS‐1b** via homolytic cleavage of the aromatic C–Si bond in **TMS‐2**, assisted by Si···F interactions. •SO• is the notation used to emphasize that SO exhibits diradical character. Note that **TMS‐2** remains stable at 160 °C in mesitylene under conditions where SO is not generated.

As with **1b** and **1c**,^[^ ^7^ ^]^ the transformation of **TMS‐1b** and **TMS‐1c** into **TMS‐2** is also expected to be initiated by the inverse electron‐demand intramolecular Diels–Alder reaction between the TMS‐protected *S*‐oxidized thiophene ring (diene) and the F_4_‐benzene ring (dienophile) (Scheme 4). The key difference between the two systems lies in the subsequent extrusion step: the former releases highly reactive SO, whereas the latter releases thermodynamically stable SO_2_. In both cases, these extrusion processes are accompanied by the elimination of two bridgehead fluorine atoms, driven by aromatization toward the coronene framework. The formation of **Mes‐2** as a byproduct suggests the involvement of radical intermediates, since such reactivity cannot be readily explained by ionic or concerted mechanisms. We therefore propose that SO, which exhibits diradical character similar to O_2_,^[^ ^20^ ^]^ induces homolytic cleavage of the aromatic C–Si bond in **TMS‐2** under the present reaction conditions (160 °C, mesitylene) (Scheme 4). Upon TMS removal, the resulting F_2_‐coronenyl radical **2**• can either abstract a hydrogen atom from the solvent mesitylene to yield **2** or undergo direct arylation with mesitylene to form **Mes‐2**. The radical nature of SO, particularly its reactivity at the S atom, is reminiscent of thiyl radicals (RS•), which have recently been reported to mediate radical desilylation of arylsilanes.^[^ ^21^ ^]^ This resemblance further supports the involvement of SO in TMS removal via a radical pathway.

### 2.3 Impact of Fluorine on the TMS Group Removal

**Scheme 5:**
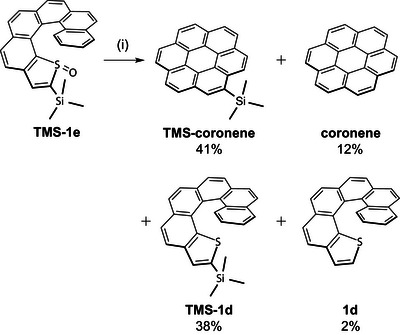
Thermal transformation of **TMS‐1e**. Conditions: (i) mesitylene, 160 °C, 3 h.

Deoxygenated **TMS‐1a** was detected in the reaction mixture of **TMS‐1b**, but no further desilylation to **1a** was observed (Scheme 3a), suggesting that Si···F interactions play a significant role in promoting TMS group removal. To further investigate the role of fluorine, we examined the coronene conversion of the non‐fluorinated *S*‐oxide **TMS‐1e** (Scheme 5). Heating **TMS‐1e** in mesitylene at 160 °C for 3 h afforded **TMS‐coronene** (41%) and **coronene** (12%), corresponding to 53% coronene conversion, which is lower than the 73% observed for **TMS‐1b**. Desilylation also occurred in the absence of fluorine, confirming that the fluorine atoms eliminated during the transformation of **TMS‐1b** were not directly responsible for TMS cleavage. Instead, desilylation is likely facilitated by in‐situ‐generated SO. Prolonged heating led to unavoidable deoxygenation of the thiophene *S*‐oxide ring, yielding **TMS‐1d** (38%) and its desilylated counterpart **1d** (2%).

The desilylation behavior between the fluorinated and non‐fluorinated systems showed a marked contrast. In the fluorinated system, desilylation occurred selectively at **TMS‐2**, whereas in the non‐fluorinated system, it proceeded from both **TMS‐coronene** and **TMS‐1d**. Furthermore, the extent of desilylation was significantly lower in the non‐fluorinated system: the total yield of desilylated products was 14% (**coronene**: 12%, **1d**: 2%), whereas it reached 41% in the fluorinated system (**2**: 35%, **Mes‐2**: 6%). Since SO is expected to be generated in both systems, this contrast indicates that SO alone cannot account for the observed desilylation behavior. As shown in Scheme 4, Si···F interactions are proposed to act synergistically with SO to promote TMS removal. As previously observed in the TMS‐free system,^[^ ^7^ ^]^ fluorine substitution also accelerated coronene conversion, with **TMS‐1e** requiring 3 h for completion versus just 30 min for **TMS‐1b**. These experimental results collectively demonstrate that fluorine plays a dual role: enhancing the efficiency of the coronene transformation and assisting SO‐induced desilylation through Si···F interactions.

## 3 Conclusion

In summary, we demonstrated that TMS‐protected F_4_‐thia[6]helicene *S*‐oxide **TMS‐1b** undergoes a highly efficient “helix‐to‐disc” conversion under thermal conditions. While thiophene *S*‐oxides are inherently more reactive than their *S*,*S*‐dioxide counterparts, the transformation of F_4_‐thia[6]helicene *S*‐oxide **1b** into F_2_‐coronene **2** had previously been challenging because of competing side reactions. In this study, the introduction of a TMS group at the thiophene *S*‐oxide ring was found to prevent its self‐condensation, thereby enabling its selective coronene conversion. Note that the TMS group was eliminated during the transformation, yielding not only **TMS‐2** but also **2** and **Mes‐2**, the latter featuring a mesityl group attached to the coronene core from solvent mesitylene. Mechanistic investigations indicated that this unexpected desilylation results from the combined effect of Si···F interactions and in‐situ‐generated SO. The suppressed desilylation in the non‐fluorinated system highlights the crucial role of fluorine in promoting TMS group removal. These findings provide new insights into the distinct roles of silicon, fluorine, and in‐situ‐generated SO in facilitating cascade transformations of helicenes and expand the synthetic utility of thiophene *S*‐oxides in the development of functionalized polycyclic aromatic systems.

## 4 Experimental Section

### Thermal transformation of TMS‐1b

A mesitylene solution of **TMS‐1b** (27.8 mg, 0.0562 mmol in 28 mL of mesitylene, 2.0 mM) was heated at 160 °C for 30 min. After confirming completion of the reaction by TLC, the solution was passed through a silica gel column (hexane/toluene = 10:1), then triturated with MeOH (2 × 1 mL) and hexane/CHCl_3_ (3:1, 2 × 4 mL). The resulting precipitate was collected and further purified by recycling preparative GPC (CHCl_3_) to give **TMS‐2** (7.31 mg, 0.0179 mmol, 32%), **2** (6.62 mg, 0.0197 mmol, 35%), **Mes‐2** (1.50 mg, 3.30 × 10^−3^ mmol, 6%), and **TMS‐1a** (3.88 mg, 8.11 × 10^−3^ mmol, 14%).

**TMS‐2**: pale yellow solid; TLC (hexane/CHCl_3_ = 3:1) *R*_f_ = 0.68; M.p. 268.5–270.0 °C; ^1^H NMR (500 MHz, CDCl_3_, 298 K) *δ* = 9.11 (s, 1H), 8.81 (d, 1H, *J* = 8.6 Hz), 8.73 (d, 1H, *J* = 8.4 Hz), 8.70 (d, 1H, *J* = 8.4 Hz), 8.66 (d, 1H, *J* = 8.4 Hz), 8.64 (d, 1H, *J* = 8.4 Hz), 8.63 (d, 1H, *J* = 8.6 Hz), 8.61 (d, 1H, *J* = 8.4 Hz), 8.56 (d, 1H, *J* = 8.4 Hz), 0.79 (d, 9H, ^6^ *J*_HF_ = 4.1 Hz); Chemical shifts highly depend on the sample concentration; ^19^F NMR (470 MHz, CDCl_3_, 298 K) *δ* = −140.0 (doublet of decets, 1F, ^3^ *J*_FF_ = 18.2 Hz, ^6^ *J*_HF_ = 4.1 Hz), −153.3 (d, 1F, ^3^ *J*_FF_ = 18.2 Hz); ^13^C{^1^H, ^19^F} NMR (125 MHz, CDCl_3_, 298 K) *δ* = 145.1 (CF), 144.3 (CF), 135.6 (CH), 131.9 (C), 129.1 (C), 128.5 (C), 128.0 (C), 127.3 (C), 126.8 (CH), 126.4 (CH), 126.3 (CH+CH+CH), 126.00 (CH), 125.96 (CH), 123.0 (C), 122.0 (C), 121.6 (C), 121.3 (C+C), 119.6 (C), 119.4 (C), 119.2 (C), 117.7 (CH), 1.14 (SiMe_3_); HR‐MS (APCI) *m*/*z* calcd for [M]^+^: 408.1140, found: 408.1135.

**Mes‐2**: pale orange solid; TLC (hexane/CHCl_3_ = 3:1) *R*_f_ = 0.63; M.p. > 285.0 °C (decomp.); ^1^H NMR (500 MHz, CDCl_3_, 298 K) *δ* = 9.09 (d, 1H, *J* = 8.7 Hz), 8.99 (d, 1H, *J* = 8.8 Hz), 8.97 (d, 1H, *J* = 8.5 Hz), 8.96 (d, 1H, *J* = 8.4 Hz), 8.94 (d, 1H, *J* = 8.1 Hz), 8.94 (d, 1H, *J* = 8.1 Hz), 8.92 (d, 1H, *J* = 8.5 Hz), 8.89 (d, 1H, *J* = 8.5 Hz), 8.72 (s, 1H), 7.15 (s, 2H), 2.50 (s, 3H), 2.04 (s, 6H); Chemical shifts highly depend on the sample concentration; ^19^F NMR (470 MHz, CDCl_3_, 298 K) *δ* = −146.5 (d, 1F, ^3^ *J*_FF_ = 17.6 Hz), −152.2 (d, 1F, ^3^ *J*_FF_ = 17.6 Hz); ^13^C{^1^H, ^19^F} NMR (125 MHz, CDCl_3_, 298 K) *δ* = 145.0 (CF), 144.6 (CF), 139.2 (C), 136.9 (C), 136.1 (2C), 134.0 (C), 129.4 (CH), 129.2 (C), 129.0 (C), 128.6 (C), 128.5 (C), 127.9 (2CH), 127.4 (CH), 126.9 (CH), 126.8 (CH), 126.6 (CH), 126.52 (CH), 126.50 (CH), 126.2 (CH), 122.2 (C), 122.1 (C), 122.0 (C), 121.5 (C), 120.8 (C), 120.0 (C), 119.9 (C), 118.9 (C), 118.2 (CH), 21.3 (CH_3_), 20.9 (2CH_3_); HR‐MS (APCI) *m*/*z* calcd for [M+H]^+^: 455.1606, found: 455.1602.

### Thermal transformation of TMS‐1c

A mesitylene solution of **TMS‐1c** (28.1 mg, 0.0550 mmol in 27.5 mL of mesitylene, 2.0 mM) was heated at 160 °C for 80 min. After confirming completion of the reaction by TLC, the reaction mixture was concentrated *in vacuo*. The resulting residue was passed through a silica gel column (hexane/toluene = 10:1), then triturated with MeOH (2 × 1 mL). The precipitate was collected and further purified by recycling preparative GPC (CHCl_3_) to give **TMS‐2** (3.3 mg, 8.1 × 10^−3^ mmol, 15%). The reaction also gave numerous complex byproducts, but thorough analysis confirmed that neither **2** nor **Mes‐2** was present.

### TMS‐Deprotection of TMS‐2

**TMS‐2** (5.0 mg, 0.012 mmol) was dissolved in THF (0.25 mL), and Bu_4_NF in THF (1 M, 0.040 mL) was added dropwise. The resulting solution was stirred at room temperature for 3 h. The reaction mixture was then quenched with saturated NH_4_Cl solution (3 mL), and the resulting precipitate was triturated with water (2 × 3 mL) to give **2** (3.3 mg). The supernatant was extracted with toluene, and the combined organic layer was concentrated. The resulting residue was further triturated with water (2 × 1 mL) to give an additional portion of **2** (0.7 mg). In total, **2** was obtained quantitatively as a pale yellow solid (4.0 mg, 0.012 mmol), showing analytical purity in its NMR spectra.

### Thermal transformation of TMS‐1e

A mesitylene solution of **TMS‐1e** (19.9 mg, 0.0471 mmol in 23.5 mL of mesitylene, 2.0 mM) was heated at 160 °C for 3 h. After confirming completion of the reaction by TLC, the solution was passed through a silica gel column (hexane) and triturated with MeOH (2 × 1 mL). The resulting precipitate was collected and found to contain **TMS‐coronene** and **coronene**. The supernatant was concentrated *in vacuo*, and the residue was purified by column chromatography on silica gel (hexane) to give **TMS‐coronene**, **coronene**, **TMS‐1d**, and **1d**. In total, **TMS‐coronene** (7.14 mg, 0.0192 mmol, 41%), **coronene** (1.72 mg, 5.73 × 10^−3^ mmol, 12%), **TMS‐1d** (7.33 mg, 0.0180 mmol, 38%), and **1d** (0.33 mg, 9.87 × 10^−4^ mmol, 2%) were obtained.

**TMS‐coronene**: pale yellow solid; TLC (hexane/CHCl_3_ = 3:1) *R*_f_ = 0.61; M.p. 256.0–258.0 °C; ^1^H NMR (500 MHz, CDCl_3_, 298 K) *δ* = 9.18 (d, 1H, *J* = 8.7 Hz), 9.11 (s, 1H), 8.93 (d, 1H, *J* = 8.7 Hz), 8.90–8.85 (m, 8H), 0.82 (s, 9H); Chemical shifts highly depend on the sample concentration; ^13^C{^1^H} NMR (125 MHz, CDCl_3_, 298 K) *δ* = 136.5 (C), 133.6 (CH), 132.3 (C), 129.0 (C), 128.7 (C), 128.6 (C), 128.3 (C), 127.8 (C), 126.6 (CH), 126.34 (CH), 126.27 (CH), 126.19 (CH), 126.15 (CH+CH), 126.2 (CH), 126.1 (CH), 126.0 (CH), 125.7 (CH), 123.2 (C), 123.0 (C), 122.7 (C), 122.53 (C), 122.45 (C), 122.4 (C), 0.72 (SiMe_3_); HR‐MS (APCI) *m*/*z* calcd for [M]^+^: 372.1329, found: 372.1332.

## Supporting Information

The authors have cited additional references within the Supporting Information.^[^ ^22^, ^23^, ^24^, ^25^ ^]^

## Conflict of Interest

The authors declare no conflict of interest.

## Supporting information



Supporting Information

Supporting Information

## Data Availability

The data that support the findings of this study are available in the supplementary material of this article.
