# Supplementary material for: Transformation of Silyl‐Protected Tetrafluorinated Thia[6]helicene S‐Oxide into a Difluorinated Coronene via Induced Desilylation
Source: Chemistry. 2025 Aug 25;31(57):e02242. doi: 10.1002/chem.202502242 (PMC12520054; doi:10.1002/chem.202502242)
Supplement: Supplementary file 1 — Supporting Information [file CHEM-31-e02242-s002.pdf]

Supporting Information

Transformation of Silyl-Protected Tetrafluorinated Thia[6]helicene *S*-Oxide into a Difluorinated Coronene via Induced Desilylation

Ayumu Nakao[1], Hiroshi Katagiri[2], and Takashi Murase[1*]

[1]Faculty of Science, Yamagata University,

1-4-12 Kojirakawa-machi, Yamagata, Yamagata 990-8560, Japan.

[2]Graduate School of Organic Materials Science, Yamagata University,

4-3-16 Jonan, Yonezawa, Yamagata 992-8510, Japan.

*E-mail: tmurase@sci.kj.yamagata-u.ac.jp

Contents

1. Materials and General Methods **3**

2. Synthesis and Physical properties **4**

2-1. Synthesis of **TMS-1a** 4

2-2. Synthesis of **TMS-1b** and **TMS-1c** 5

2-3. Thermal transformation of **TMS-1b** 6

2-4. Thermal transformation of **TMS-1c** 7

2-5. TMS-Deprotection of **TMS-2** 8

2-6. Synthesis of **TMS-1d** 8

2-7. Synthesis of **TMS-1e** 9

2-8. Thermal transformation of **TMS-1e** 10

3. NMR Monitoring the stability of 1b and TMS-1b (Figure S1) **11**

4. Single-crystal X-ray crystallography **11**

TMS-2 (Table S1, Figure S2) 13

Mes-2 (Table S1, Figure S3) 14

5. NMR spectra **15**

TMS-1a (Figures S4–S8) 15

TMS-1b (Figures S9–S13) 18

TMS-1c (Figures S14–S18) 21

TMS-2 (Figures S19–S24) 24

Mes-2 (Figures S25–S29) 27

TMS-1d (Figures S30–S34) 30

TMS-1e (Figures S35–S39) 33

TMS-coronene (Figures S40–S43) 36

6. MS spectra **38**

7. References **46**

1. Materials and General Methods

^{1}H, ^{13}C, ^{19}F, and other 2D NMR spectra were recorded on a JEOL JNM-ECZ-500 R (500 MHz for ^{1}H, 125 MHz for ^{13}C, and 470 MHz for ^{19}F) spectrometer at 298 K. ^{1}H and ^{13}C NMR spectra were referenced internally to tetramethylsilane (TMS) as a standard (δ = 0 ppm). ^{19}F NMR spectra were referenced internally to hexafluorobenzene (C_6F_6) in $CDCl_3$, which was sealed in a capillary tube, as a standard (δ = –164.9 ppm). ESI-TOF-MS and APCI-TOF-MS spectra were recorded on a Bruker micrOTOF II spectrometer. Melting points were determined with a Barnstead MEL-TEMP 1001D melting point apparatus. Recycling preparative HPLC (GPC) was carried out using a LC-2000 plus semi-preparative HPLC system (JASCO) equipped with a YMC-GPC T4000 column (eluent: $CHCl_3$). Photoirradiation was carried out with a high-pressure mercury lamp (HB400X-15 400 W, SEN LIGHTS CORP) in a quartz water-cooling jacket, which was placed at the center of a reaction vessel (500 or 1000 mL) for internal irradiation. TLC analyses were carried out on aluminium sheets coated with silica gel 60 (Merck 5554-1M). Solvents and reagents were purchased from commercial suppliers (TCI, FUJIFILM Wako Pure Chemical, Kanto Chemical, Nacalai Tesque, Sigma-Aldrich) and used without further purification. F_4-Thia[6]helicene **1a** and thia[6]helicene **1d** were prepared as described in our previous paper.[S1] Physical data for F_2-coronene **2** are also available therein.

2. Synthesis and Physical properties

2-1. Synthesis of **TMS-1a**

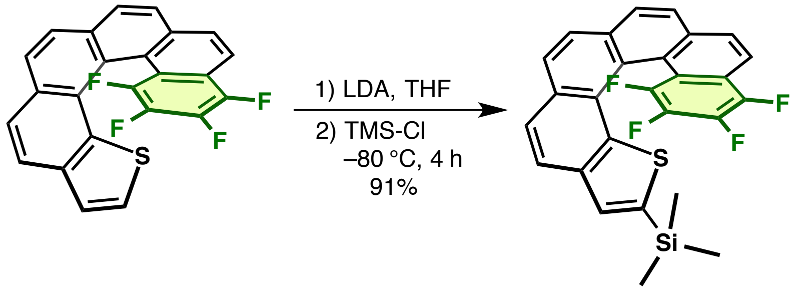

A solution of **1a** (101 mg, 0.250 mmol) in dry THF (2.5 mL) was cooled to –80 °C under a nitrogen atmosphere. LDA (2.0 M solution in THF/heptane/ethylbenzene, 0.25 mL, 0.50 mmol) was added dropwise over 3 min. After stirring for 2 h, TMS-Cl (0.063 mL, 0.50 mmol) was added dropwise over 1 min. The resulting solution was stirred at –80 °C for an additional 2 h. The reaction mixture was then quenched with water (2 mL) and extracted with EtOAc. The organic layer was washed with water, dried over anhydrous Na_2SO_4, filtered, and concentrated *in vacuo*. The residue was purified by column chromatography on silica gel (hexane) and triturated with MeOH to give **TMS-1a** (109 mg, 0.228 mmol, 91%) as a pale yellow solid.

TLC (hexane/$CHCl_3$ = 3:1) R_f = 0.60; M.p. 175.5–176.5 °C; ^{1}H NMR (500 MHz, $CDCl_3$, 298 K) δ = 8.25 (dd, 1H, *J* = 8.9 Hz, 1.1 Hz), 8.12 (d, 1H, *J* = 8.1 Hz), 8.107 (d, 1H, *J* = 8.1 Hz), 8.105 (d, 1H, *J* = 8.9 Hz), 8.03 (d, 1H+1H, *J* = 8.1 Hz), 7.96 (d, 1H, *J* = 8.4 Hz), 7.91 (d, 1H, *J* = 8.4 Hz), 7.45 (s, 1H), 0.18 (s, 9H); ^{19}F NMR (470 MHz, $CDCl_3$, 298 K) δ = –131.9 (ddd, 1F, *J* = 18.7 Hz, 14.2 Hz, 3.5 Hz), –153.8 (dd, 1F, *J* = 20.2 Hz, 13.2 Hz), –162.9 (td, 1F, *J* = 20.0 Hz, 4.4 Hz), –163.2 (t, 1F, *J* = 20.2 Hz); ^{13}C{^{1}H, ^{19}F} NMR (125 MHz, $CDCl_3$, 298 K) δ = 144.4 (CF), 142.4 (CF), 140.0 (C), 139.8 (C), 139.3 (C), 138.3 (CF), 137.9 (CF), 132.6 (C), 132.2 (C), 130.4 (CH), 129.8 (C), 129.1 (CH), 128.5 (CH+CH), 128.2 (C), 126.4 (CH), 125.3 (C), 125.0 (CH), 124.5 (CH), 123.0 (CH), 120.1 (C), 118.2 (CH), 118.1 (C), 118.0 (C), –0.50 ($SiMe_3$); HR-MS (ESI) *m*/*z* calcd for $[M+Na]^+$: 501.0727, found: 501.0717.

2-2. Synthesis of **TMS-1b** and **TMS-1c**

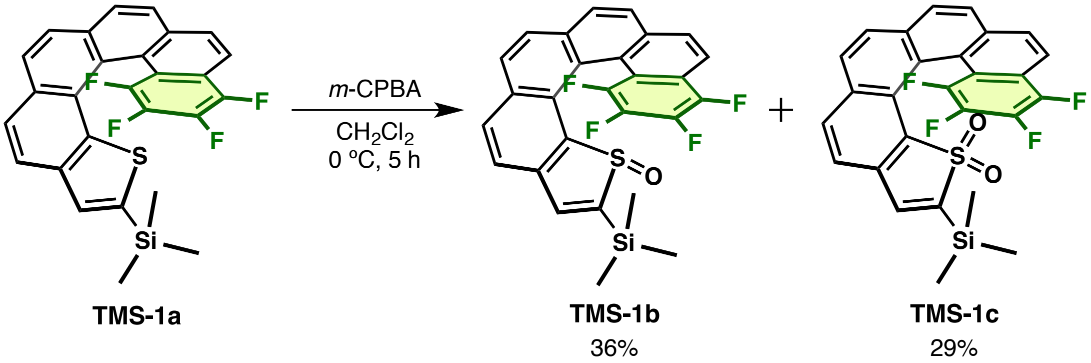

TMS-1a (116 mg, 0.242 mmol) was dissolved in CH_2Cl_2 (19 mL). To this solution, *m*-CPBA (41.9 mg, 70% wt, 0.243 mmol) was slowly added. The resulting solution was stirred at 0 °C for 5 h. The reaction mixture was washed with 10% aqueous solution of $NaHCO_3$ (3 × 20 mL) and extracted with CH_2Cl_2. The combined organic layer was washed with brine, dried over Na_2SO_4, filtered, and concentrated *in vacuo*. The residue was purified by column chromatography on silica gel ($CHCl_3$) to give **TMS-1b** (43.3 mg, 0.0875 mmol, 36%) and **TMS-1c** (35.7 mg, 0.0699 mmol, 29%), along with the recovery of unreacted **TMS-1a** (33.8 mg, 0.0706 mmol, 29%).

TMS-1b: yellow solid; TLC (hexane/EtOAc = 1:1) R_f = 0.45; M.p. > 178.0 °C (decomp.); ^{1}H NMR (500 MHz, $CDCl_3$, 298 K) δ = 8.26 (dd, 1H, *J* = 8.7 Hz, 1.2 Hz), 8.11 (d, 1H, *J* = 8.1 Hz), 8.07 (d, 1H, *J* = 8.7 Hz), 8.05 (d, 1H, *J* = 8.1 Hz), 8.01 (d, 1H, *J* = 8.1 Hz), 7.95 (d, 1H, *J* = 8.4 Hz), 7.86 (d, 1H, *J* = 8.4 Hz), 7.68 (d, 1H, *J* = 7.8 Hz), 7.13 (s, 1H), 0.18 (s, 9H); ^{19}F NMR (470 MHz, $CDCl_3$, 298 K) δ = –134.8 (ddd, 1F, *J* = 21.0 Hz, 15.0 Hz, 2.5 Hz), –152.3 (dd, 1F, *J* = 19.7 Hz, 13.7 Hz), –162.0 (td, 1F, *J* = 20.0 Hz, 3.7 Hz), –162.8 (t, 1F, *J* = 19.7 Hz); ^{13}C{^{1}H, ^{19}F} NMR (125 MHz, $CDCl_3$, 298 K) δ = 154.9 (C), 144.8 (CF), 142.9 (CF), 142.5 (C), 139.8 (CH), 138.9 (CF), 138.4 (CF), 138.2 (C), 133.4 (C), 132.3 (CH), 132.2 (C), 131.4 (C), 129.6 (C), 128.7 (CH), 128.5 (CH), 128.1 (CH), 128.0 (CH), 126.7 (CH), 123.32 (C), 123.27 (CH), 121.2 (C), 119.1 (C), 118.8 (CH), 116.7 (C), –1.34 ($SiMe_3$); HR-MS (ESI) *m*/*z* calcd for [M+Na]$^+$: 517.0676, found: 517.0678.

TMS-1c: yellow solid; TLC (hexane/EtOAc = 1:1) R_f = 0.71; M.p. > 212.0 °C (decomp.); ^{1}H NMR (500 MHz, $CDCl_3$, 298 K) δ = 8.23 (dd, 1H, *J* = 8.7 Hz, 1.2 Hz), 8.15 (d, 1H, *J* = 8.1 Hz), 8.08 (d, 1H, *J* = 8.1 Hz), 8.02 (d, 1H, *J* = 9.0 Hz), 7.97 (d, 1H, *J* = 8.1 Hz), 7.93 (d, 1H, *J* = 8.4 Hz), 7.86 (d, 1H, *J* = 8.4 Hz), 7.47 (d, 1H, *J* = 8.1 Hz), 7.10 (s, 1H), 0.17 (s, 9H); ^{19}F NMR (470 MHz, $CDCl_3$, 298 K) δ = –137.7 ~ –137.9 (m, 1F), –152.9 (dd, 1F, *J* = 20.2 Hz, 13.2 Hz), –162.5 (t, 1F, *J* = 19.7 Hz), –162.8 (td, 1F, *J* = 20.2 Hz, 4.05 Hz); ^{13}C{^{1}H, ^{19}F} NMR (125 MHz, $CDCl_3$, 298 K) δ = 147.7 (C), 144.3 (CF), 142.8 (CF), 139.1 (CF), 138.2 (CF), 138.1 (CH), 137.2 (C), 134.1 (C), 133.1 (CH), 132.9 (C), 132.8 (C), 132.3 (C), 129.0 (CH), 128.3 (C), 127.7 (CH), 127.2 (CH), 127.0 (CH), 126.6 (CH), 124.7 (C), 122.8 (CH), 122.7 (C), 119.9 (C), 119.1 (CH), 118.1 (C), –1.74 ($SiMe_3$); HR-MS (ESI) *m*/*z* calcd for [M+Na]$^+$: 533.0625, found: 533.0620.

2-3. Thermal transformation of **TMS-1b**

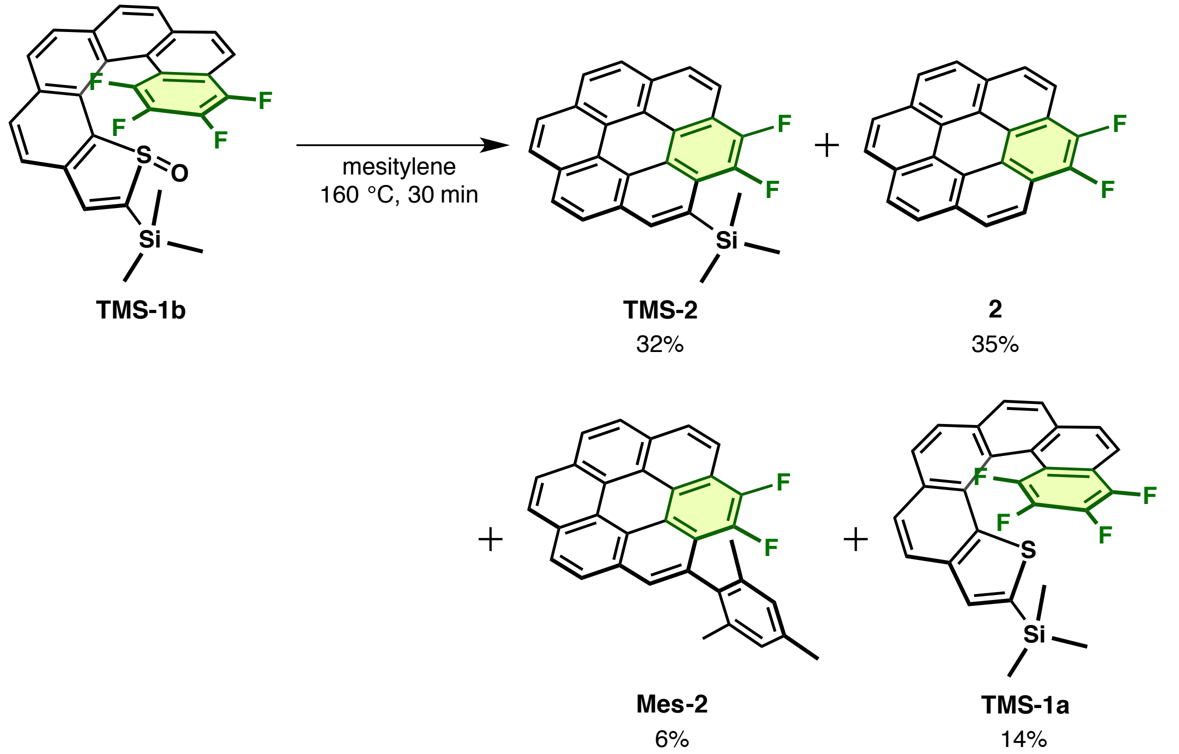

A mesitylene solution of **TMS-1b** (27.8 mg, 0.0562 mmol in 28 mL of mesitylene, 2.0 mM) was heated at 160 °C for 30 min. After confirming completion of the reaction by TLC, the solution was passed through a silica gel column (hexane/toluene = 10:1), then triturated with MeOH (2 × 1 mL) and hexane/$CHCl_3$ (3:1, 2 × 4 mL). The resulting precipitate was collected and further purified by recycling preparative GPC ($CHCl_3$) to give **TMS-2** (7.31 mg, 0.0179 mmol, 32%), **2** (6.62 mg, 0.0197 mmol, 35%), **Mes-2** (1.50 mg, 3.30×10^{-3} mmol, 6%), and **TMS-1a** (3.88 mg, 8.11×10^{-3} mmol, 14%).

TMS-2: pale yellow solid; TLC (hexane/$CHCl_3$ = 3:1) R_f = 0.68; M.p. 268.5–270.0 °C; ^{1}H NMR (500 MHz, $CDCl_3$, 298 K) δ = 9.11 (s, 1H), 8.81 (d, 1H, J = 8.6 Hz), 8.73 (d, 1H, J = 8.4 Hz), 8.70 (d, 1H, J = 8.4 Hz), 8.66 (d, 1H, J = 8.4 Hz), 8.64 (d, 1H, J = 8.4 Hz), 8.63 (d, 1H, J = 8.6 Hz), 8.61 (d, 1H, J = 8.4 Hz), 8.56 (d, 1H, J = 8.4 Hz), 0.79 (d, 9H, $^6J_{HF}$ = 4.1 Hz); *Chemical shifts highly depend on the sample concentration*; ^{19}F NMR (470 MHz, $CDCl_3$, 298 K) δ = –140.0 (doublet of decets, 1F, $^3J_{FF}$ = 18.2 Hz, $^6J_{HF}$ = 4.1 Hz), –153.3 (d, 1F, $^3J_{FF}$ = 18.2 Hz); ^{13}C{^{1}H, ^{19}F} NMR (125 MHz, $CDCl_3$, 298 K) δ = 145.1 (CF), 144.3 (CF), 135.6 (CH), 131.9 (C), 129.1 (C), 128.5 (C), 128.0 (C), 127.3 (C), 126.8 (CH), 126.4 (CH), 126.3 (CH+CH+CH), 126.00 (CH), 125.96 (CH), 123.0 (C), 122.0 (C), 121.6 (C), 121.3 (C+C), 119.6 (C), 119.4 (C), 119.2 (C), 117.7 (CH), 1.14 ($SiMe_3$); HR-MS (APCI) m/z calcd for $[M]^+$: 408.1140, found: 408.1135.

Mes-2: pale orange solid; TLC (hexane/$CHCl_3$ = 3:1) R_f = 0.63; M.p. > 285.0 °C (decomp.); ^{1}H NMR (500 MHz, $CDCl_3$, 298 K) δ = 9.09 (d, 1H, J = 8.7 Hz), 8.99 (d, 1H, J = 8.8 Hz), 8.97 (d, 1H, J = 8.5 Hz), 8.96 (d, 1H, J = 8.4 Hz), 8.94 (d, 1H, J = 8.1 Hz), 8.94 (d, 1H, J = 8.1 Hz), 8.92 (d, 1H,

J = 8.5 Hz), 8.89 (d, 1H, J = 8.5 Hz), 8.72 (s, 1H), 7.15 (s, 2H), 2.50 (s, 3H), 2.04 (s, 6H); *Chemical shifts highly depend on the sample concentration*; ^{19}F NMR (470 MHz, $CDCl_3$, 298 K) δ = –146.5 (d, 1F, $^3J_{FF}$ = 17.6 Hz), –152.2 (d, 1F, $^3J_{FF}$ = 17.6 Hz); ^{13}C{^{1}H, ^{19}F} NMR (125 MHz, $CDCl_3$, 298 K) δ = 145.0 (CF), 144.6 (CF), 139.2 (C), 136.9 (C), 136.1 (2C), 134.0 (C), 129.4 (CH), 129.2 (C), 129.0 (C), 128.6 (C), 128.5 (C), 127.9 (2CH), 127.4 (CH), 126.9 (CH), 126.8 (CH), 126.6 (CH), 126.52 (CH), 126.50 (CH), 126.2 (CH), 122.2 (C), 122.1 (C), 122.0 (C), 121.5 (C), 120.8 (C), 120.0 (C), 119.9 (C), 118.9 (C), 118.2 (CH), 21.3 (CH_3), 20.9 ($2CH_3$); HR-MS (APCI) *m*/*z* calcd for $[M+H]^+$: 455.1606, found: 455.1602.

2-4. Thermal transformation of **TMS-1c**

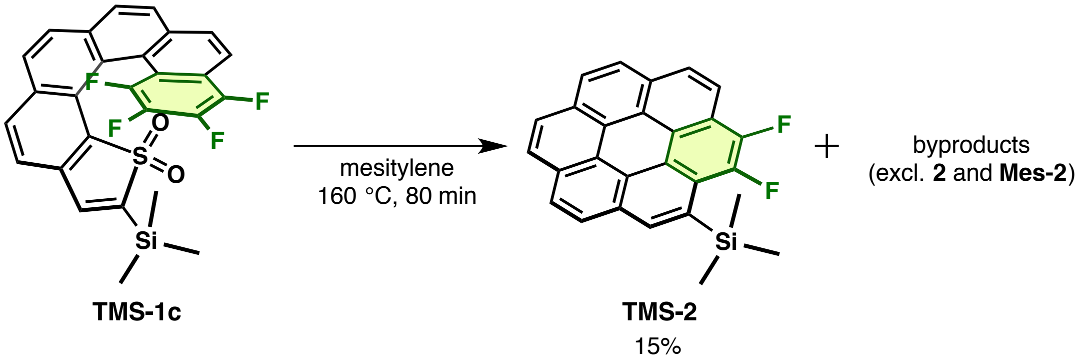

A mesitylene solution of **TMS-1c** (28.1 mg, 0.0550 mmol in 27.5 mL of mesitylene, 2.0 mM) was heated at 160 °C for 80 min. After confirming completion of the reaction by TLC, the reaction mixture was concentrated *in vacuo*. The resulting residue was passed through a silica gel column (hexane/toluene = 10:1), then triturated with MeOH (2 × 1 mL). The precipitate was collected and further purified by recycling preparative GPC ($CHCl_3$) to give **TMS-2** (3.3 mg, 8.1×10^{-3} mmol, 15%). The reaction also gave numerous complex byproducts, but thorough analysis confirmed that neither **2** nor **Mes-2** was present.

2-5. TMS-Deprotection of **TMS-2**

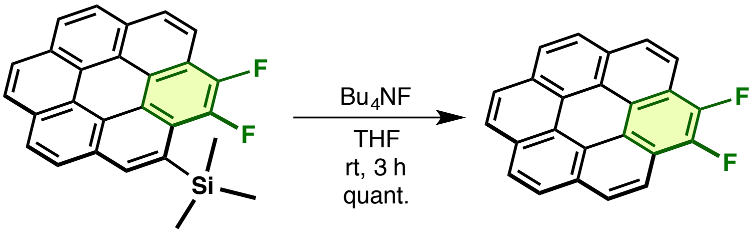

TMS-2   **2**

TMS-2 (5.0 mg, 0.012 mmol) was dissolved in THF (0.25 mL), and Bu_4NF in THF (1 M, 0.040 mL) was added dropwise. The resulting solution was stirred at room temperature for 3 h. The reaction mixture was then quenched with saturated NH_4Cl solution (3 mL), and the resulting precipitate was triturated with water (2 × 3 mL) to give **2** (3.3 mg). The supernatant was extracted with toluene, and the combined organic layer was concentrated. The resulting residue was further triturated with water (2 × 1 mL) to give an additional portion of **2** (0.7 mg). In total, **2** was obtained quantitatively as a pale yellow solid (4.0 mg, 0.012 mmol), showing analytical purity in its NMR spectra.

2-6. Synthesis of **TMS-1d**

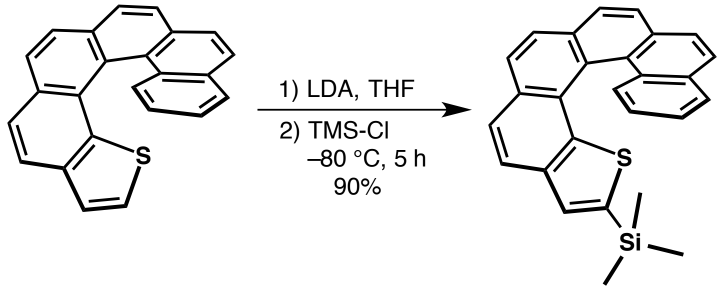

1d   **TMS-1d**

A solution of **1d** (133 mg, 0.398 mmol) in dry THF (4 mL) was cooled to –80 °C under a nitrogen atmosphere. LDA (2.0 M solution in THF/heptane/ethylbenzene, 0.40 mL, 0.80 mmol) was added dropwise over 2 min. After stirring for 2 h, TMS-Cl (0.151 mL, 1.20 mmol) was added dropwise over 1 min. The resulting solution was stirred at –80 °C for an additional 3 h. The reaction mixture was then quenched with water (4 mL) and extracted with EtOAc. The organic layer was washed with water, dried over anhydrous Na_2SO_4, filtered, and concentrated *in vacuo*. The residue was purified by column chromatography on silica gel (hexane/$CHCl_3$ = 5:1) to give **TMS-1d** (146 mg, 0.359 mmol, 90%) as a pale yellow solid.

TLC (hexane/$CHCl_3$ = 3:1) R_f = 0.54; M.p. 132.5–134.0 °C; ^{1}H NMR (500 MHz, $CDCl_3$, 298 K) δ = 8.06 (d, 1H, J = 8.4 Hz), 8.01 (d, 1H, J = 9.0 Hz), 7.992 (d, 1H, J = 8.4 Hz), 7.989 (d, 1H, J = 8.1 Hz), 7.98 (d, 1H, J = 9.0 Hz), 7.96 (d, 1H, J = 8.1 Hz), 7.95 (dd, 1H, J = 8.4 Hz, 1.5 Hz), 7.94

(d, 1H, J = 8.4 Hz), 7.91 (d, 1H, J = 8.4 Hz), 7.89 (d, 1H, J = 8.4 Hz), 7.37 (s, 1H), 7.35 (td, 1H, J = 7.4 Hz, 1.2 Hz), 6.90 (td, 1H, J = 7.7 Hz, 1.2 Hz), 0.10 (s, 9H); $^{13}C\{^1H\}$ NMR (125 MHz, $CDCl_3$, 298 K) δ = 141.6 (C), 141.0 (C), 139.5 (C), 132.8 (C), 131.9 (C), 131.6 (C), 130.7 (C), 130.5 (C), 129.3 (CH), 128.0 (CH+CH), 127.6 (CH), 127.3 (C), 127.2 (CH), 127.1 (CH), 126.5 (CH), 126.2 (CH), 126.1 (C), 125.8 (CH), 125.3 (CH), 125.0 (CH), 124.8 (CH), 124.2 (C), 122.6 (CH), –0.47 ($SiMe_3$); HR-MS (ESI) m/z calcd for $[M+Na]^+$: 429.1104, found: 429.1095.

2-7. Synthesis of **TMS-1e**

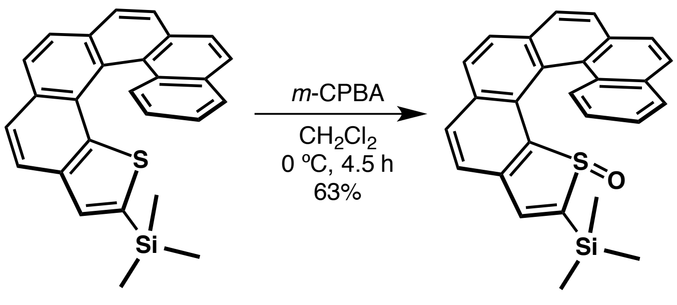

TMS-1d **TMS-1e**

TMS-1d (110 mg, 0.269 mmol) was dissolved in CH_2Cl_2 (21 mL). To this solution, *m*-CPBA (46.6 mg, 70% wt, 0.270 mmol) was slowly added. The resulting solution was stirred at 0 °C for 4.5 h. The reaction mixture was washed with 10% aqueous solution of $NaHCO_3$ (3 × 20 mL) and extracted with CH_2Cl_2. The combined organic layer was washed with brine, dried over Na_2SO_4, filtered, and concentrated *in vacuo*. The residue was purified by column chromatography on silica gel ($CHCl_3$) to give **TMS-1e** (71.9 mg, 0.170 mmol, 63%) as a yellow solid.

TLC (hexane/EtOAc = 1:1) R_f = 0.41; M.p. > 175.0 °C (decomp.); ^1H NMR (500 MHz, $CDCl_3$, 298 K) δ = 8.06 (d, 1H, J = 7.8 Hz), 8.02 (d, 1H, J = 8.7 Hz), 8.004 (d, 1H, J = 8.4 Hz), 7.994 (d, 1H, J = 7.8 Hz), 7.988 (d, 1H, J = 8.1 Hz), 7.94 (d, 1H, J = 8.4 Hz), 7.88 (d, 1H, J = 8.4 Hz), 7.86 (d, 1H, J = 7.2 Hz), 7.84 (d, 1H, J = 8.4 Hz), 7.61 (d, 1H, J = 8.1 Hz), 7.45 (td, 1H, J = 6.9 Hz, 1.2 Hz), 7.09 (td, 1H, J = 6.9 Hz, 1.2 Hz), 7.06 (s, 1H), 0.11 (s, 9H); $^{13}C\{^1H\}$ NMR (125 MHz, $CDCl_3$, 298 K) δ = 155.5 (C), 145.8 (C), 139.9 (CH), 138.2 (C), 133.8 (C), 132.7 (C), 132.14 (C), 132.10 (CH), 131.6 (C), 129.8 (C), 128.8 (CH), 128.5 (C), 128.4 (CH), 128.2 (CH), 127.8 (CH), 127.4 (CH), 127.3 (C), 126.9 (CH), 126.6 (CH), 126.4 (CH), 126.1 (CH), 125.1 (CH), 122.7 (CH), 122.3 (C), –1.38 ($SiMe_3$); HR-MS (ESI) m/z calcd for $[M+Na]^+$: 445.1053, found: 445.1054.

2-8. Thermal transformation of **TMS-1e**

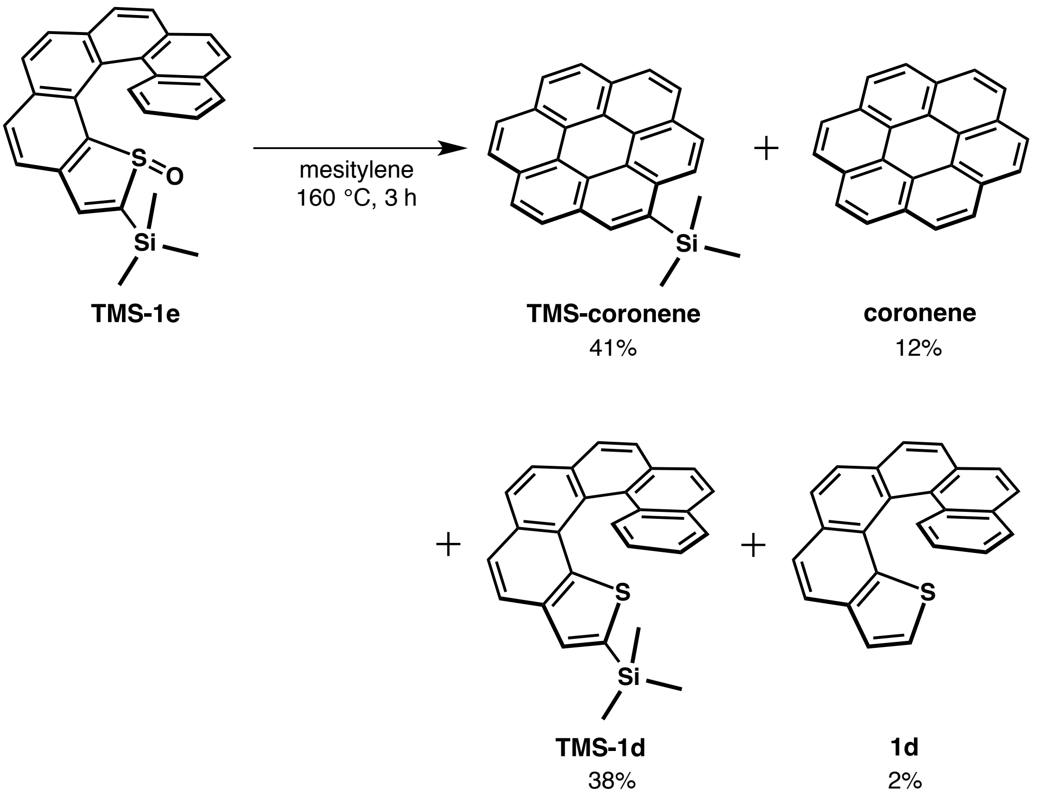

A mesitylene solution of **TMS-1e** (19.9 mg, 0.0471 mmol in 23.5 mL of mesitylene, 2.0 mM) was heated at 160 °C for 3 h. After confirming completion of the reaction by TLC, the solution was passed through a silica gel column (hexane) and triturated with MeOH (2 × 1 mL). The resulting precipitate was collected and found to contain **TMS-coronene** and **coronene**. The supernatant was concentrated *in vacuo*, and the residue was purified by column chromatography on silica gel (hexane) to give **TMS-coronene**, **coronene**, **TMS-1d**, and **1d**. In total, **TMS-coronene** (7.14 mg, 0.0192 mmol, 41%), **coronene** (1.72 mg, 5.73×10^{-3} mmol, 12%), **TMS-1d** (7.33 mg, 0.0180 mmol, 38%), and **1d** (0.33 mg, 9.87×10^{-4} mmol, 2%) were obtained.

TMS-coronene: pale yellow solid; TLC (hexane/$CHCl_3$ = 3:1) R_f = 0.61; M.p. 256.0–258.0 °C; ^{1}H NMR (500 MHz, $CDCl_3$, 298 K) δ = 9.18 (d, 1H, J = 8.7 Hz), 9.11 (s, 1H), 8.93 (d, 1H, J = 8.7 Hz), 8.90–8.85 (m, 8H), 0.82 (s, 9H); *Chemical shifts highly depend on the sample concentration*; ^{13}C{^{1}H} NMR (125 MHz, $CDCl_3$, 298 K) δ = 136.5 (C), 133.6 (CH), 132.3 (C), 129.0 (C), 128.7 (C), 128.6 (C), 128.3 (C), 127.8 (C), 126.6 (CH), 126.34 (CH), 126.27 (CH), 126.19 (CH), 126.15 (CH+CH), 126.2 (CH), 126.1 (CH), 126.0 (CH), 125.7 (CH), 123.2 (C), 123.0 (C), 122.7 (C), 122.53 (C), 122.45 (C), 122.4 (C), 0.72 ($SiMe_3$); HR-MS (APCI) m/z calcd for $[M]^+$: 372.1329, found: 372.1332.

3. NMR Monitoring the stability of 1b and TMS-1b

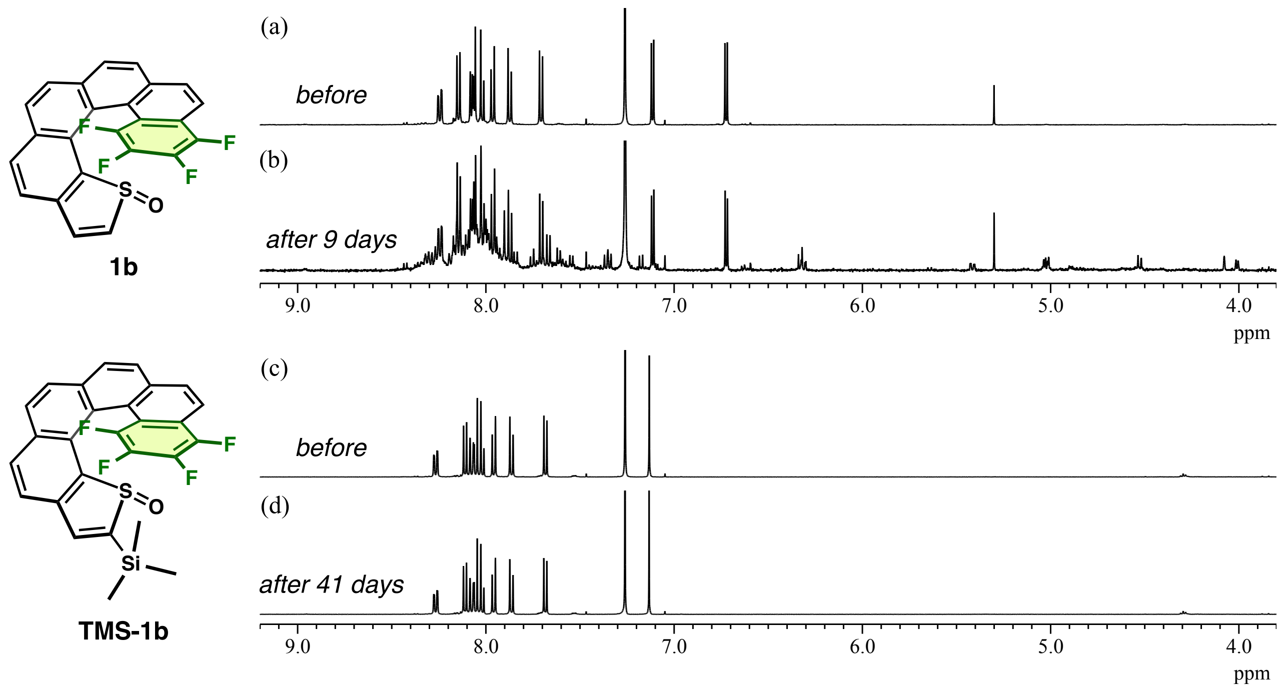

Figure S1. NMR Monitoring of the stability of **1b** and **TMS-1b**. A $CDCl_3$ solution of each compound was left to stand in an NMR test tube at room temperature. ^1H NMR spectra (500 MHz, 298 K, $CDCl_3$) of **1b**: (a) before and (b) after 9 days; and **TMS-1b**: (c) before and (d) after 41 days.

4. Single-crystal X-ray crystallography

X-ray diffraction data for **TMS-2** and **Mes-2** were collected on a Rigaku XtaLAB Synergy-S diffractometer using Mo $K\alpha$ radiation (λ = 0.71075 Å). Single crystals suitable for X-ray analysis were obtained by slow evaporation of CH_2Cl_2/MeOH (2:1) solutions of the compounds at room temperature. The crystal data and structure refinements are listed in Table S1, and the packing structures are shown in Figures S1 and S2. Data collection, cell refinements, and data reductions were conducted using CrysAlisPro software.[S2] These structures were solved by direct methods using the SHELXT program[S3] and refined by the full-matrix least-squares on F^2 using the SHELXL program[S4] running with the Yadokari-XG 2009 software program.[S5] The crystallographic data have been deposited in the Cambridge Crystallographic Data Centre (CCDC) as CCDC 2466142 and 2466143.

Table S1. Crystal data and structure refinement for **TMS-2** and **Mes-2**

Compound	**TMS-2**	**Mes-2**
Empirical formula	$C_{27}H_{18}F_2Si$	$2(C_{33}H_{20}F_2)\cdot CH_2Cl_2$
Formula weight	408.50	993.90
Temperature (K)	260	100
Wavelength (Å)	0.71073	0.71073
Crystal system	Monoclinic	Monoclinic
Space group	$P2_1/n$	$C2/c$
a (Å)	11.2580(17)	11.7740(2)
b (Å)	7.0000(1)	13.4698(3)
c (Å)	24.861(4)	30.2007(7)
α (°)	90	90
β (°)	90.030(13)	100.222(2)
γ (°)	90	90
Volume (Å^3)	1959.2(5)	4713.61(17)
Z	4	4
Density (calculated) (g/cm^3)	1.385	1.401
Absorption coefficient (mm^{-1})	0.150	0.200
F(000)	848	2056
Crystal size (mm^3)	0.100 × 0.020 × 0.010	0.100 × 0.070 × 0.030
Theta range for data collection (°)	2.440 to 24.999	2.319 to 27.498
Index ranges	$-12 \le h \le 13$<br>$-8 \le k \le 8$<br>$-29 \le l \le 29$	$-15 \le h \le 15$<br>$-17 \le k \le 17$<br>$-39 \le l \le 38$
Reflections collected	15565	54163
Independent reflections	3446<br>[R(int) = 0.0737]	5435<br>[R(int) = 0.0303]
Completeness (%)	99.7<br>(theta = 24.999º)	99.9<br>(theta = 27.498º)
Absorption correction	Multi-scan	Multi-scan
Max. and min. transmission	1.00000 and 0.52382	1.00000 and 0.91572
Refinement method	Full-matrix<br>least-squares on F^2	Full-matrix<br>least-squares on F^2
Data / restrains / parameters	3446 / 51 / 275	5435 / 0 / 333
Goodness-of-fit on F^2	1.080	1.045
Final R indices [I > 2sigma(I)]	R_1 = 0.0898<br>wR_2 = 0.2072	R_1 = 0.0549<br>wR_2 = 0.1486
R indices (all data)	R_1 = 0.1360<br>wR_2 = 0.2315	R_1 = 0.0599<br>wR_2 = 0.1534
Largest diff. peak and hole (e.Å^{-3})	0.504 and –0.180	0.714 and –0.605
CCDC Number	2466143	2466142

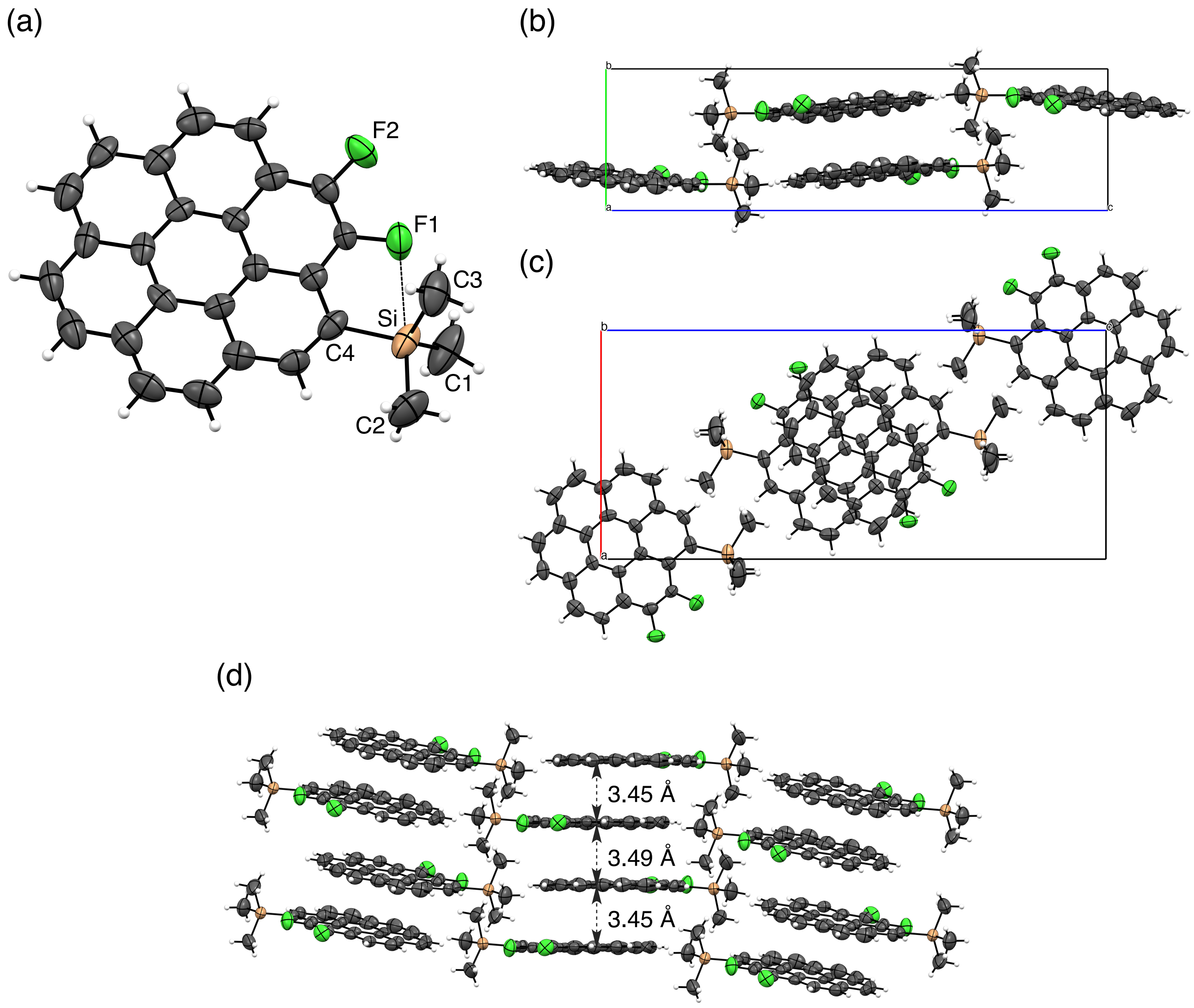

Figure S2. ORTEP drawings of **TMS-2** with 50% probability ellipsoids. (a) Asymmetric unit. Selected interatomic distances (Å): C1–Si, 1.843(9); C2–Si, 1.903(8); C3–Si, 1.840(8); C4–Si, 1.895(5); Si···F1, 2.842(4). (b–d) Crystal packing: (b) viewed along the *a*-axis (*bc* plane); (c) viewed along the *b*-axis (*ac* plane); (d) showing the interplanar distance (3.45 and 3.49 Å) between neighboring coronene cores. The coronene core is flat, with all 24 carbon atoms lying in the same plane (r.m.s. deviation = 0.0227 Å).

[Note] X-ray diffraction data for **TMS-2** were collected at 260 K, as the crystal integrity was compromised at lower temperatures. Upon cooling below 260 K, the single crystals frequently exhibited visible physical damage, such as bending or cracking. These changes were accompanied by a gradual deterioration in diffraction quality, with frames becoming increasingly disordered within one minute of exposure. Even after returning to room temperature, the crystallinity could not be restored. This behavior is likely due to anisotropic lattice contraction upon cooling, particularly along the columnar stacking axis (*b*-axis), where strong π–π interactions between coronene cores may cause significant uniaxial shrinkage. Such structural changes can lead to a loss of

single-crystal order, possibly as a result of a phase transition or mechanical stress induced by differential contraction.

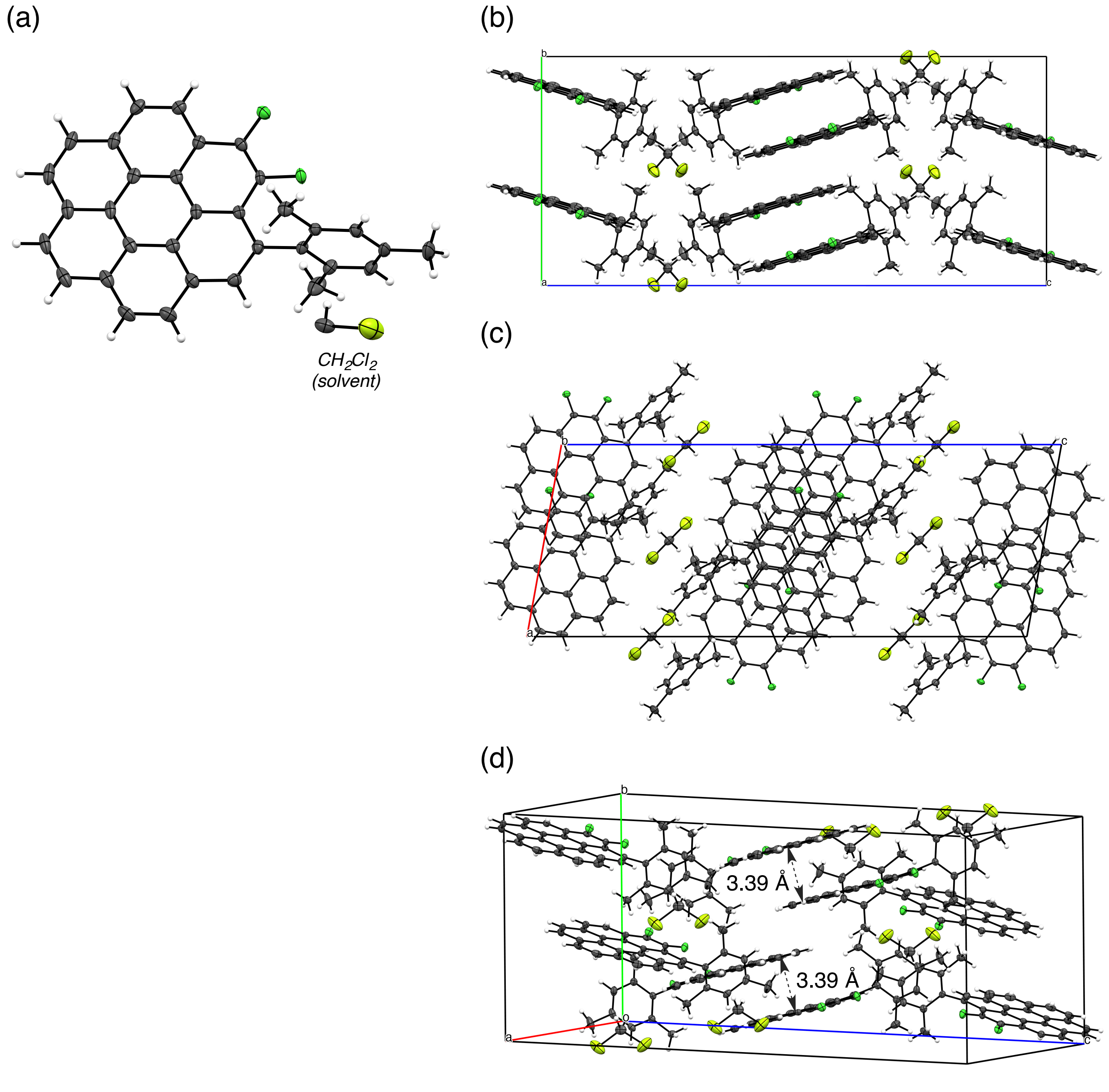

Figure S3. ORTEP drawings of **Mes-2** with 50% probability ellipsoids. (a) Asymmetric unit. (b–d) Crystal packing: (b) viewed along the *a*-axis (*bc* plane); (c) viewed along the *b*-axis (*ac* plane); (d) showing the interplanar distance (3.39 Å) between neighboring coronene cores. The coronene core is flat, with all 24 carbon atoms lying in the same plane (r.m.s. deviation = 0.0212 Å).

5. NMR and MS spectra

TMS-1a

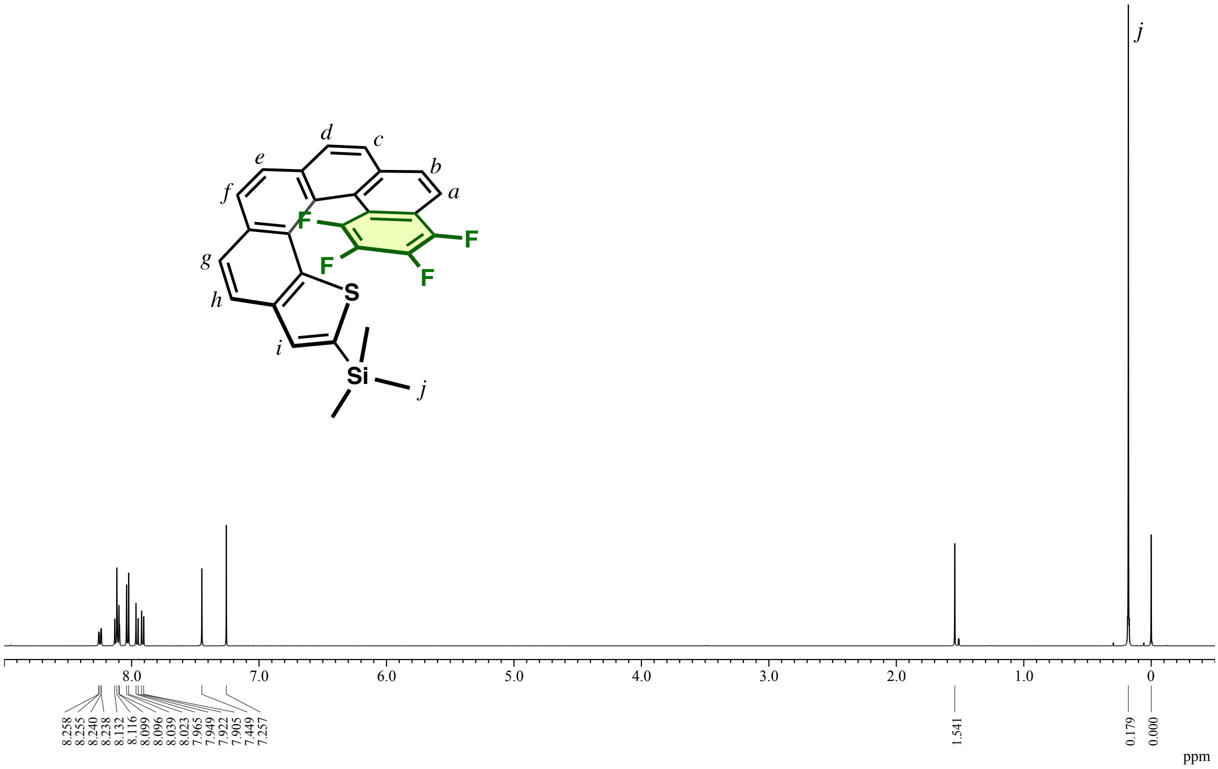

Figure S4. ^{1}H NMR spectrum (500 MHz, $CDCl_3$, 298 K) of **TMS-1a**.

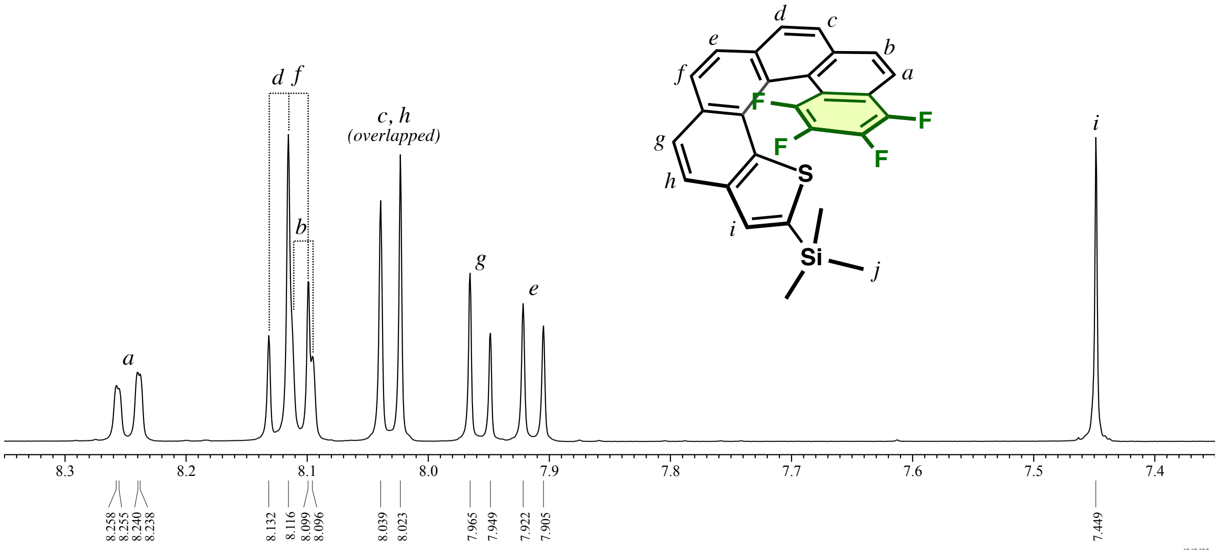

Figure S5. Enlarged ^{1}H NMR spectrum (500 MHz, $CDCl_3$, 298 K) of **TMS-1a**.

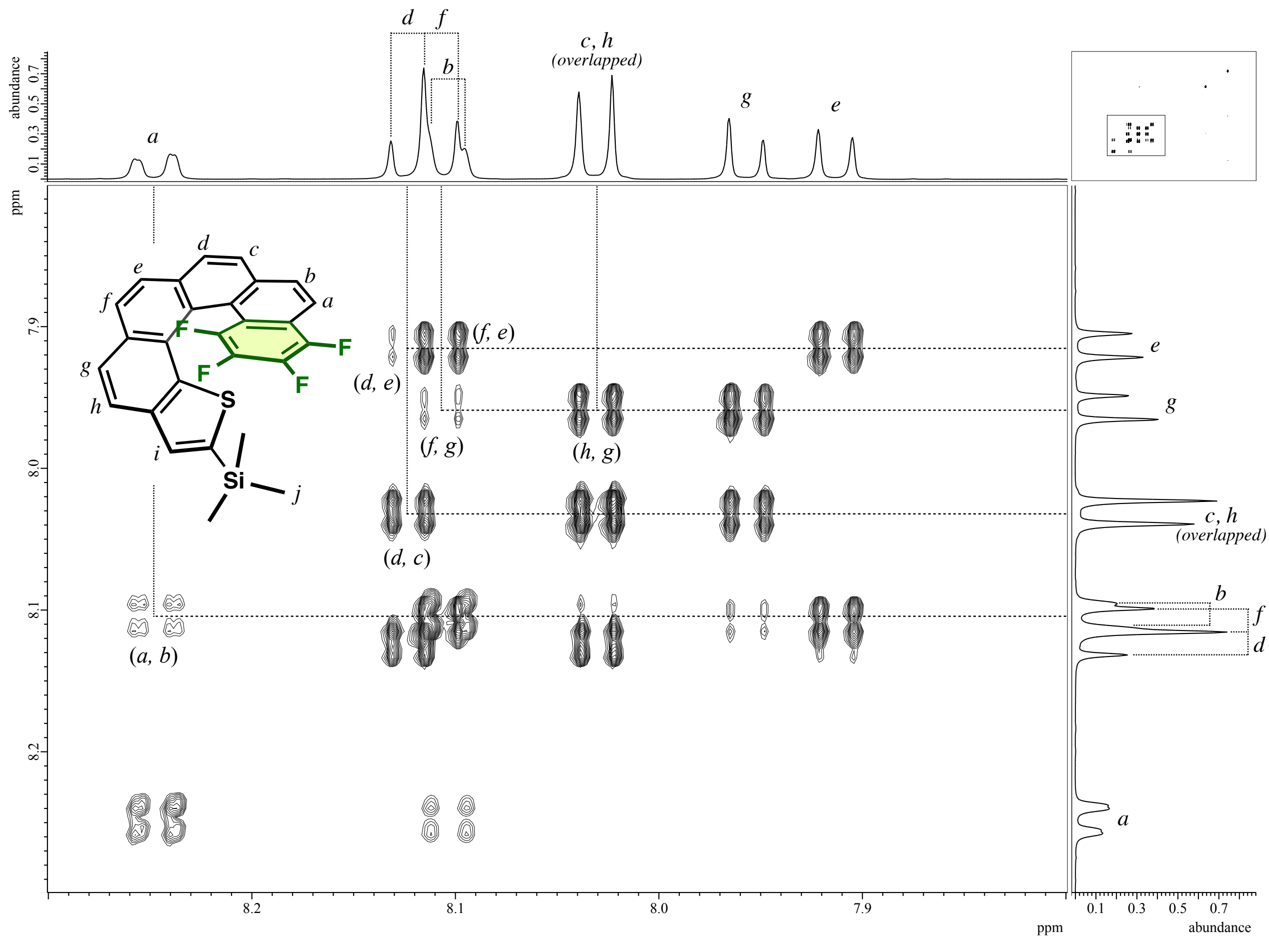

Figure S6. ^{1}H–^{1}H COSY NMR spectrum (500 MHz, $CDCl_3$, 298 K) of **TMS-1a**.

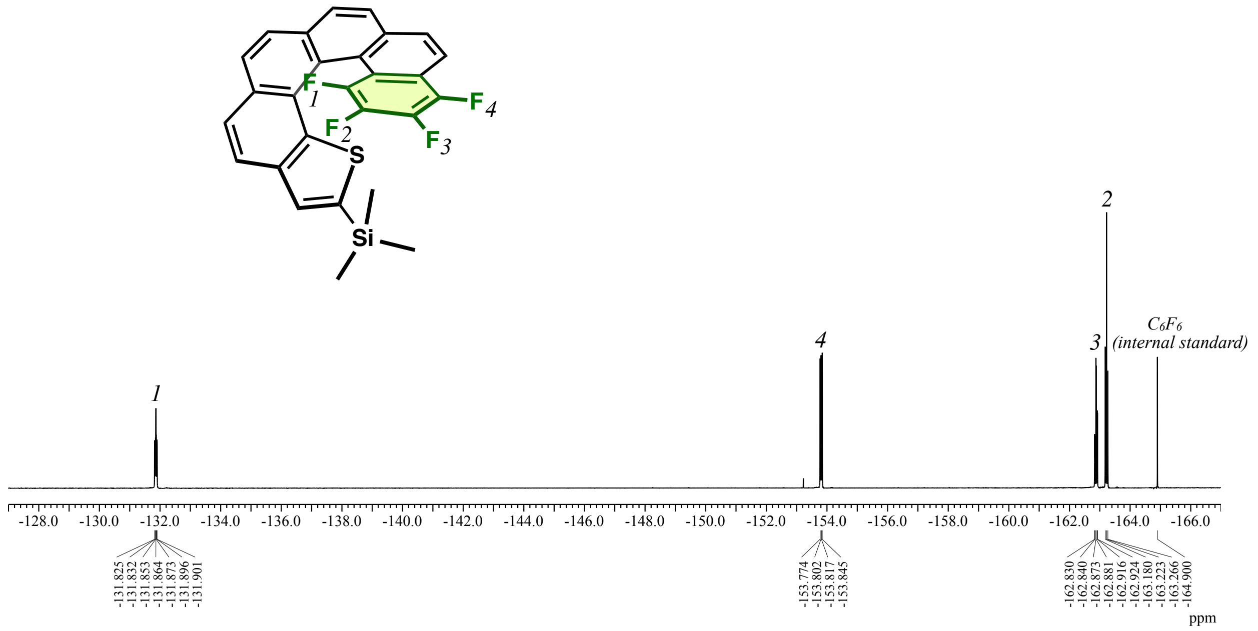

Figure S7. ^{19}F NMR spectrum (470 MHz, $CDCl_3$, 298 K) of **TMS-1a**. Signal assignments were made based on comparison with the ^{19}F NMR spectrum of **1a**.[S1]

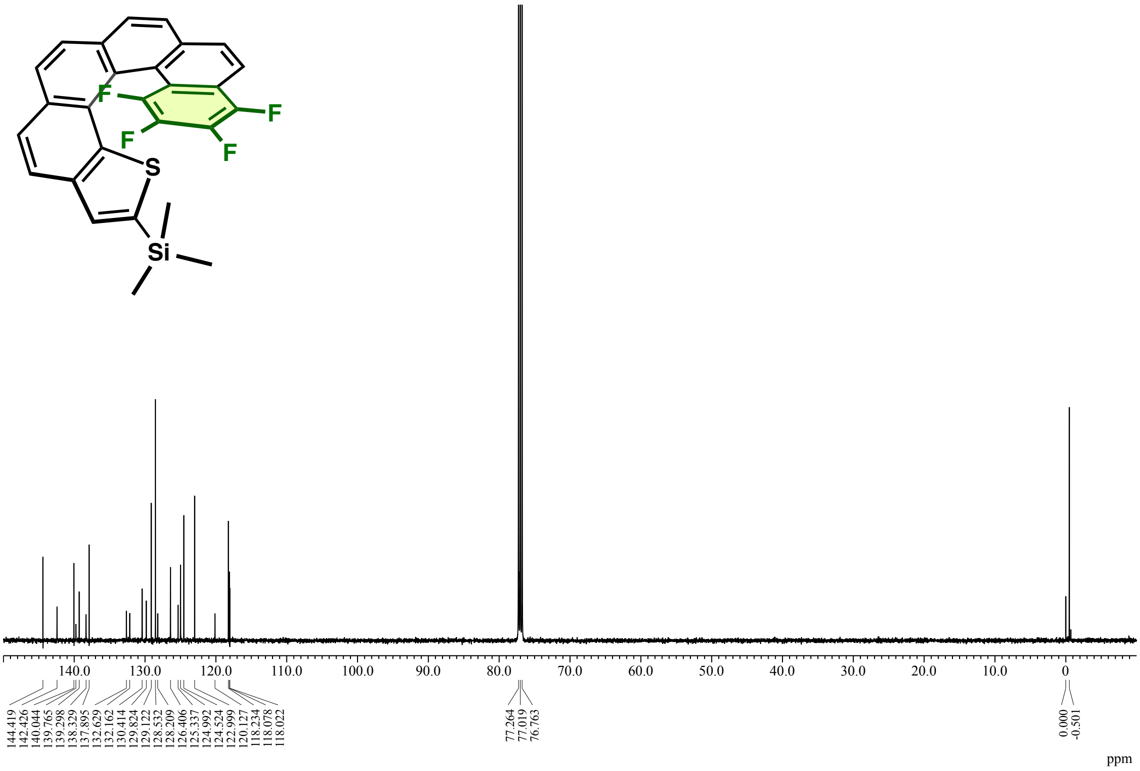

Figure S8. $^{13}C\{^{1}H, ^{19}F\}$ NMR spectrum (125 MHz, $CDCl_3$, 298 K) of **TMS-1a**.

TMS-1b

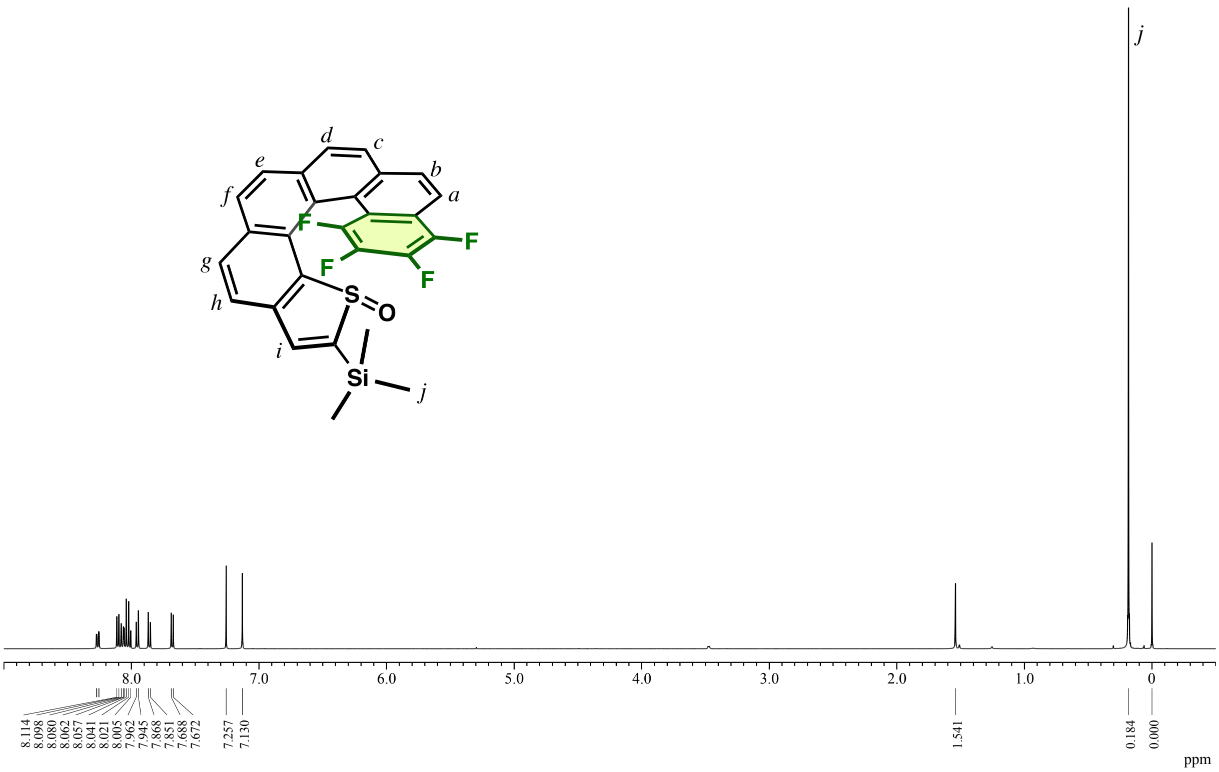

Figure S9. ^{1}H NMR spectrum (500 MHz, $CDCl_3$, 298 K) of **TMS-1b**.

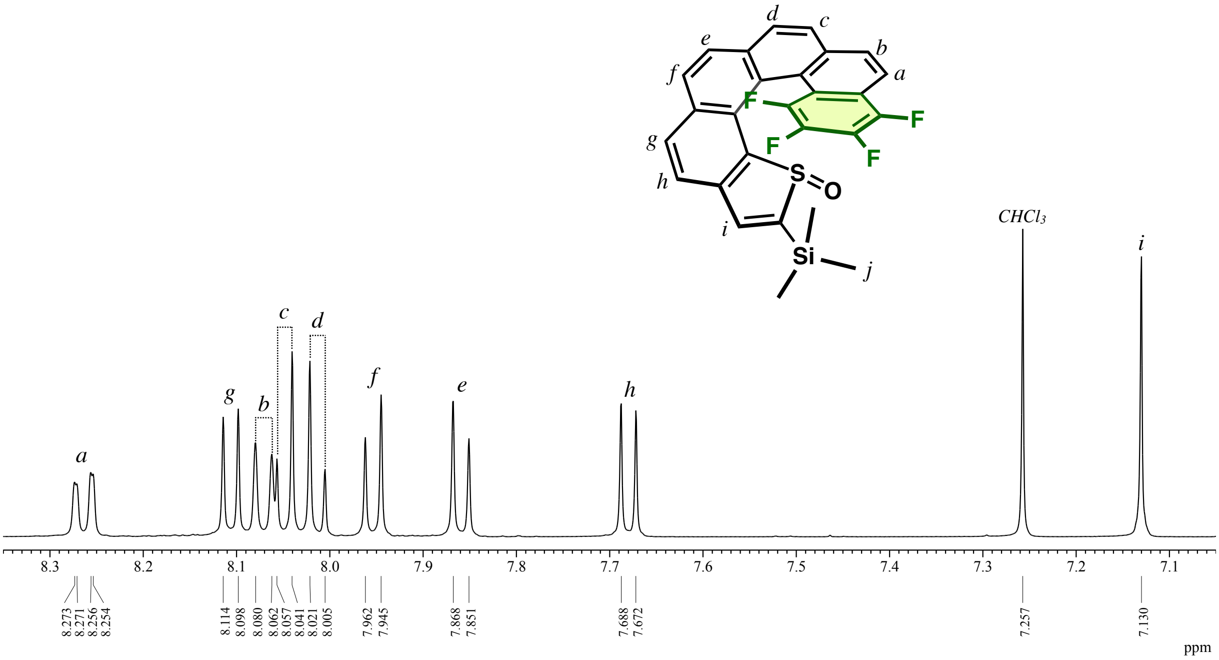

Figure S10. Enlarged ^{1}H NMR spectrum (500 MHz, $CDCl_3$, 298 K) of **TMS-1b**.

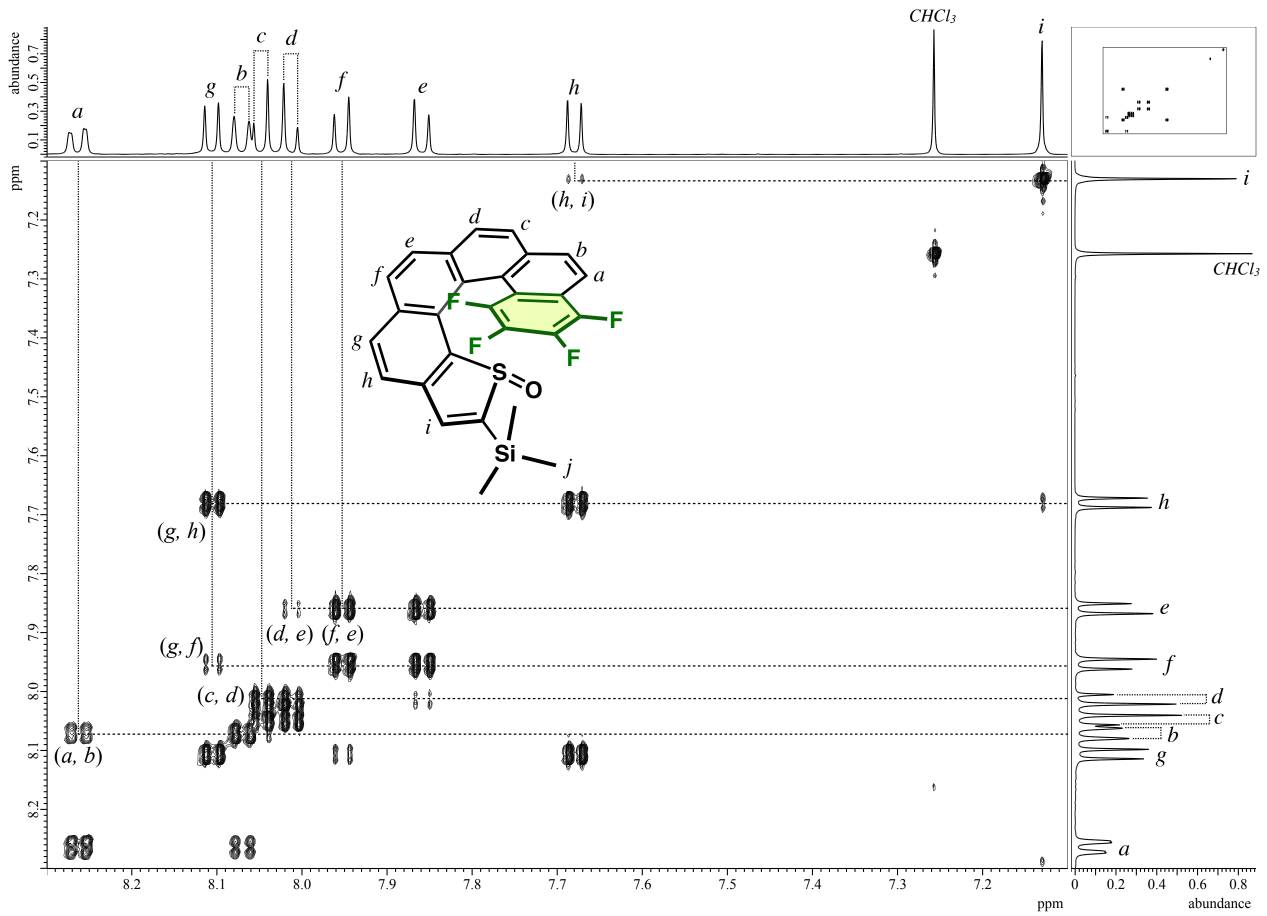

Figure S11. ^{1}H–^{1}H COSY NMR spectrum (500 MHz, $CDCl_3$, 298 K) of **TMS-1b**.

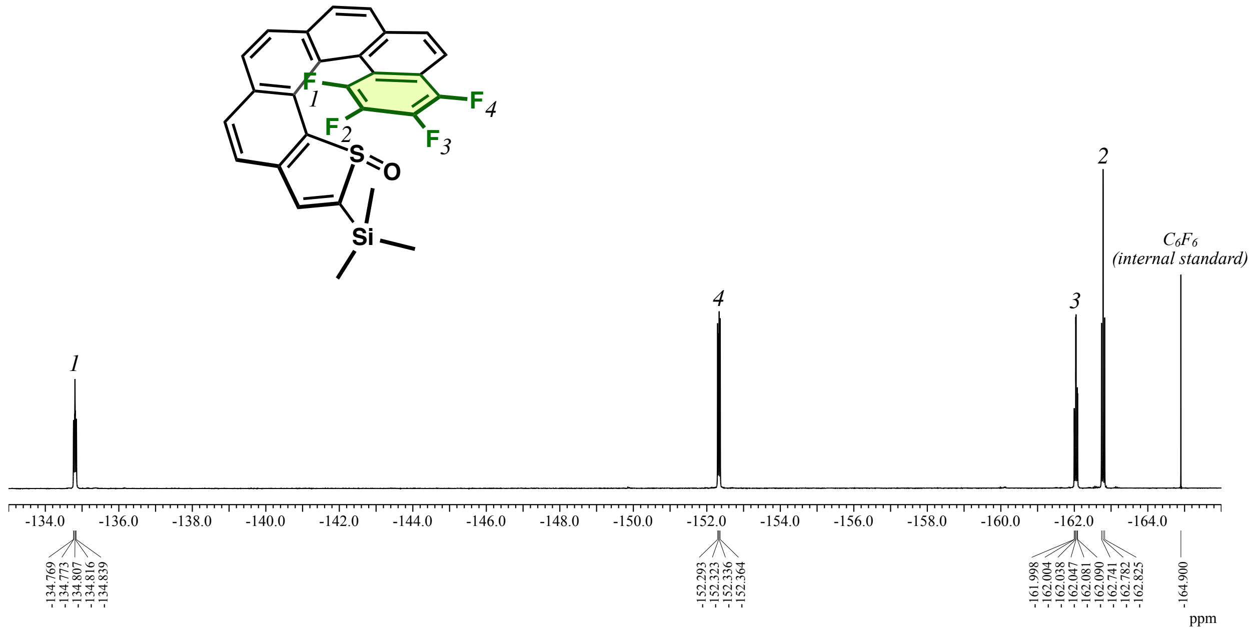

Figure S12. ^{19}F NMR spectrum (470 MHz, $CDCl_3$, 298 K) of **TMS-1b**. Signal assignments were made based on comparison with the ^{19}F NMR spectrum of **1b**.[S1]

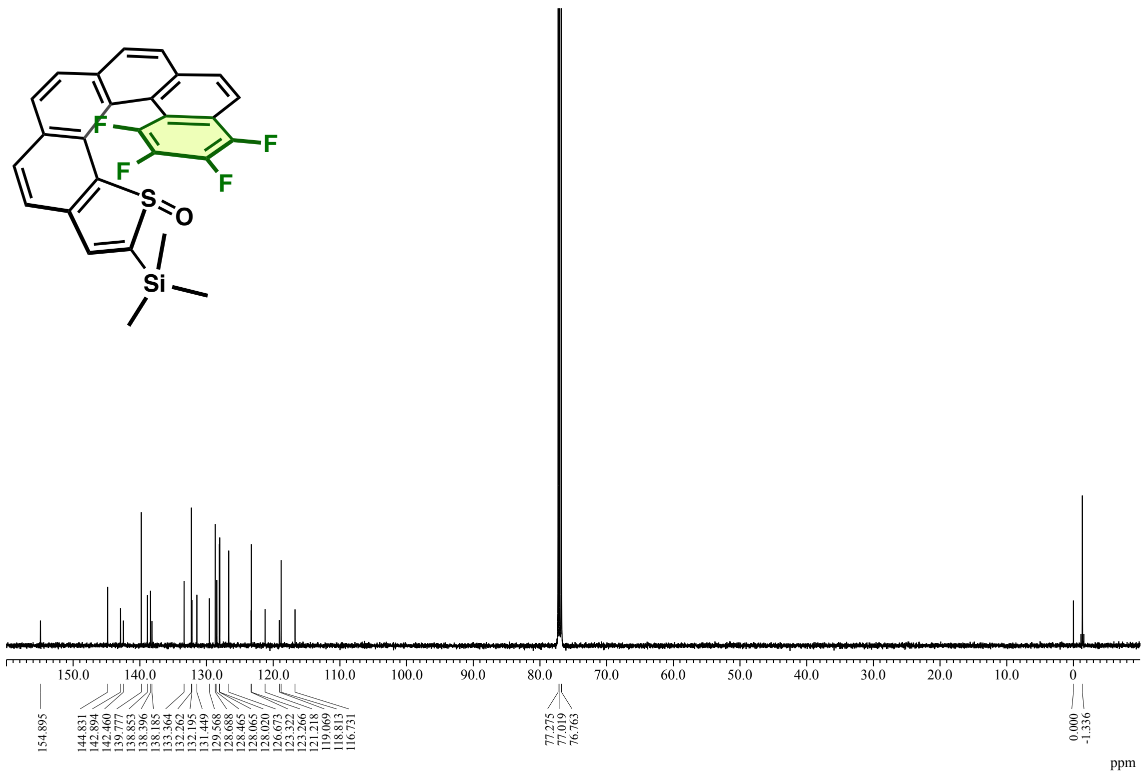

Figure S13. $^{13}C\{^{1}H, ^{19}F\}$ NMR spectrum (125 MHz, $CDCl_3$, 298 K) of **TMS-1b**.

TMS-1c

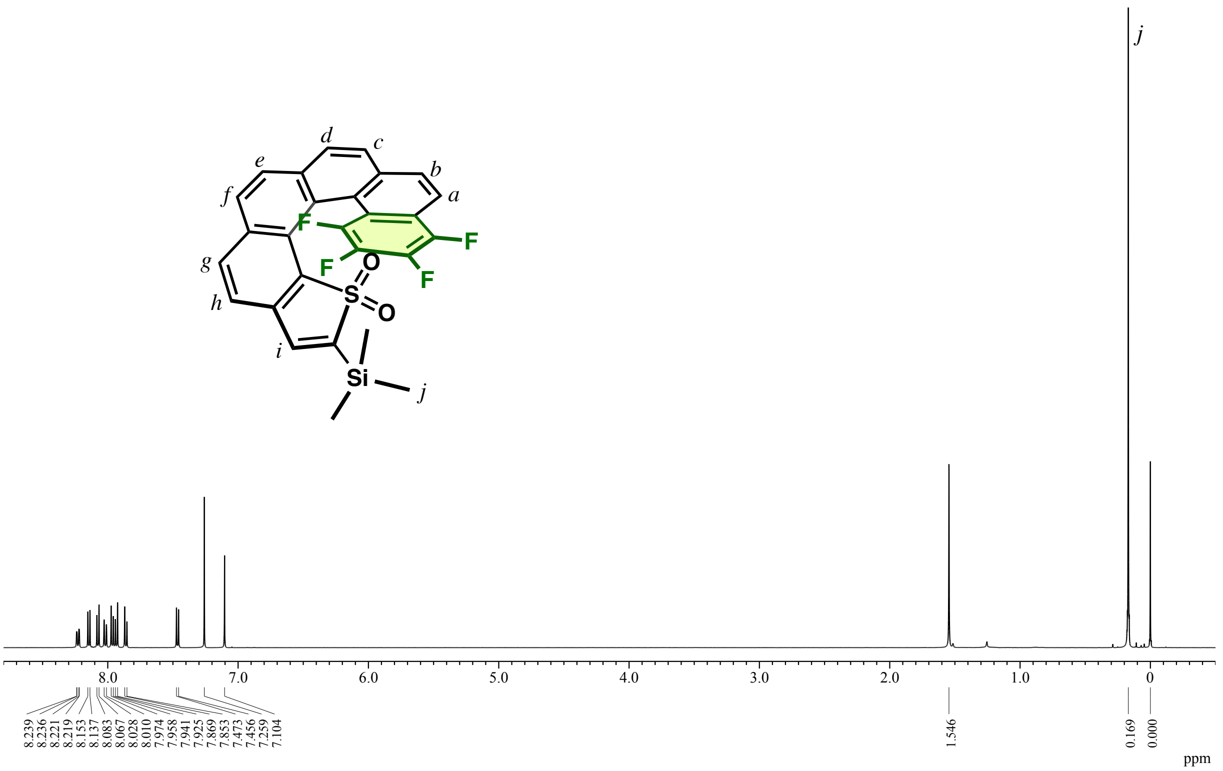

Figure S14. ^{1}H NMR spectrum (500 MHz, $CDCl_3$, 298 K) of **TMS-1c**.

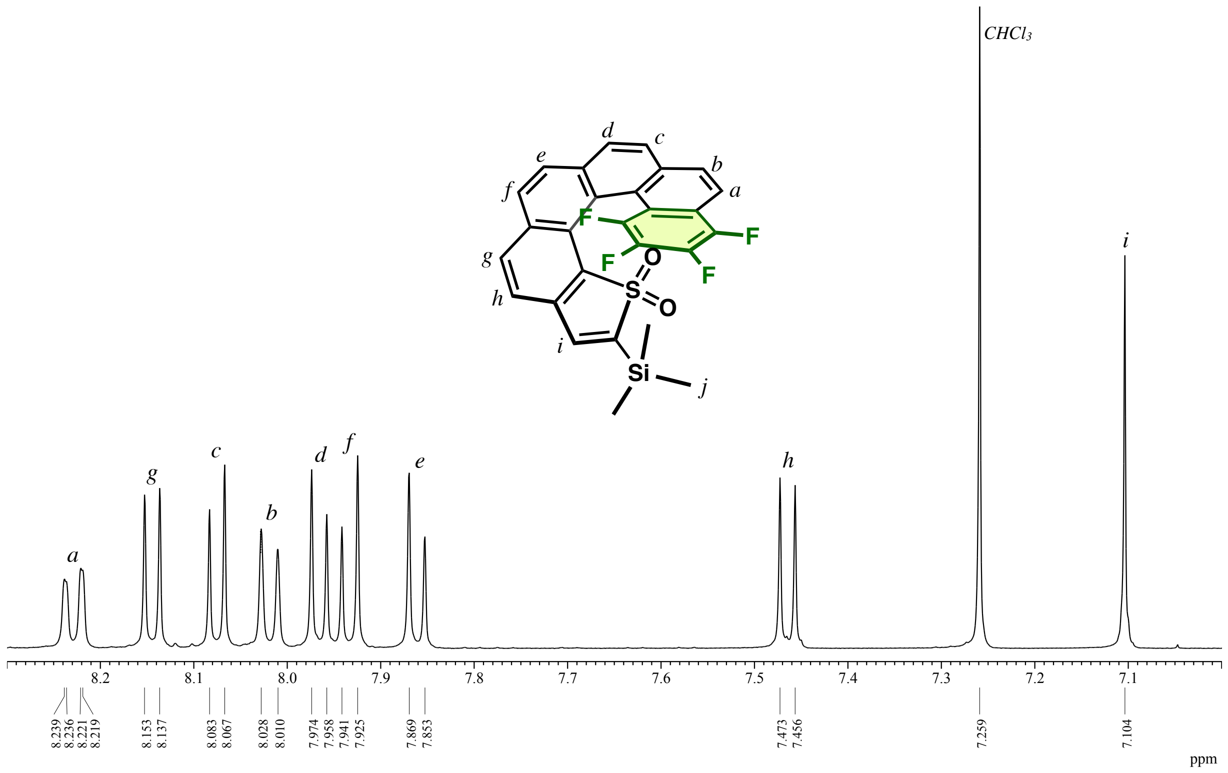

Figure S15. Enlarged ^{1}H NMR spectrum (500 MHz, $CDCl_3$, 298 K) of **TMS-1c**.

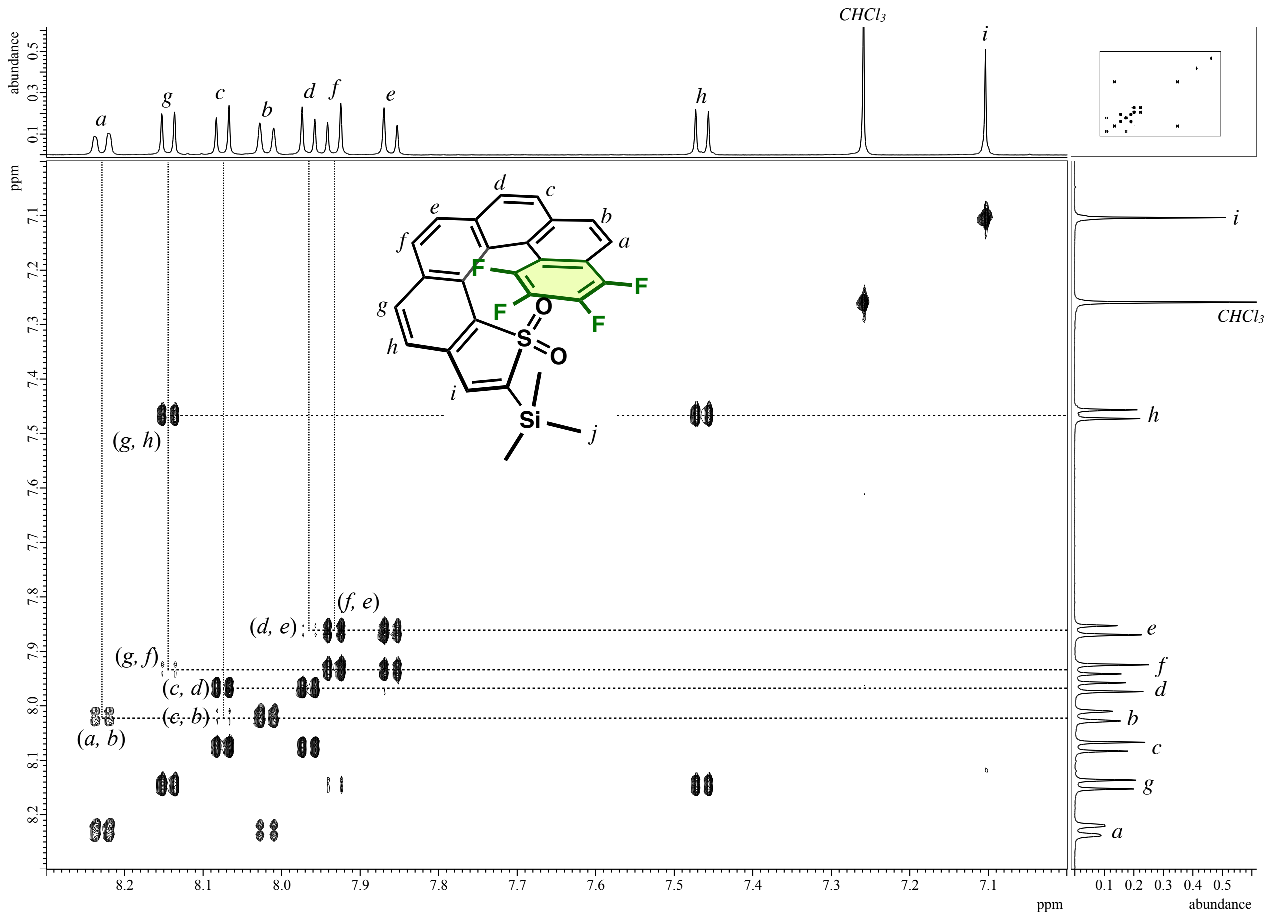

Figure S16. ^{1}H–^{1}H COSY NMR spectrum (500 MHz, $CDCl_3$, 298 K) of **TMS-1c**.

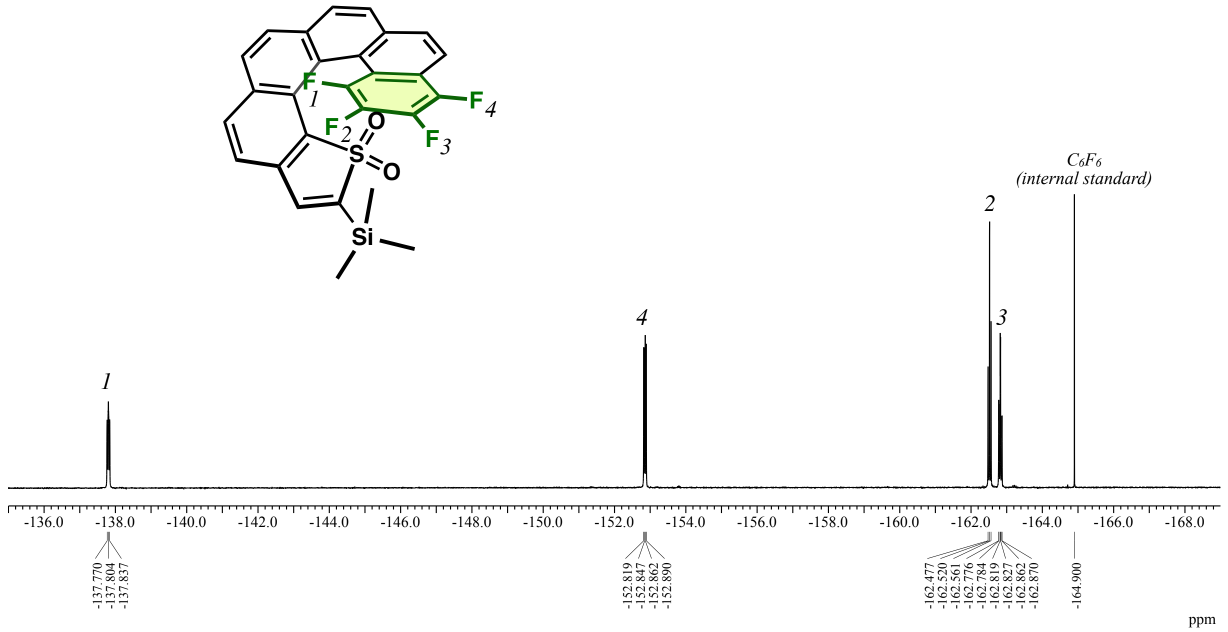

Figure S17. ^{19}F NMR spectrum (470 MHz, $CDCl_3$, 298 K) of **TMS-1c**. Signal assignments were made based on comparison with the ^{19}F NMR spectrum of **1c**.[S1]

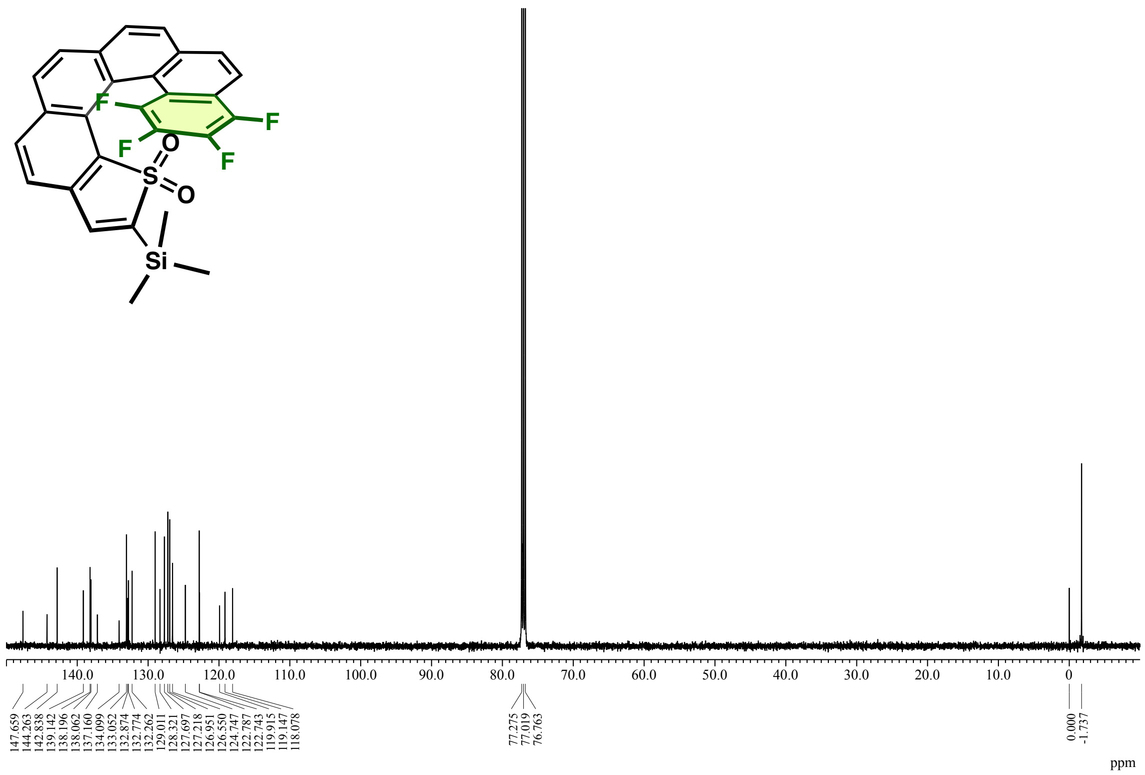

Figure S18. $^{13}C\{^{1}H, ^{19}F\}$ NMR spectrum (125 MHz, $CDCl_3$, 298 K) of **TMS-1c**.

TMS-2

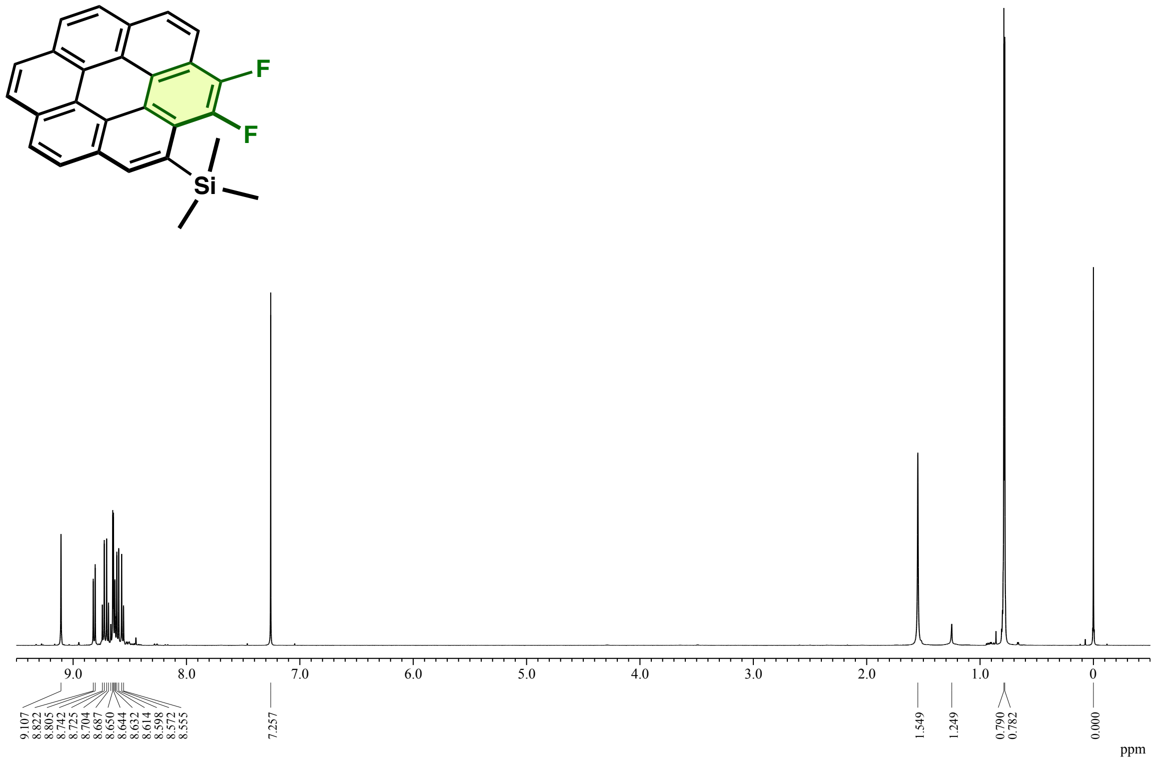

Figure S19. ^{1}H NMR spectrum (500 MHz, $CDCl_3$, 298 K) of **TMS-2**.

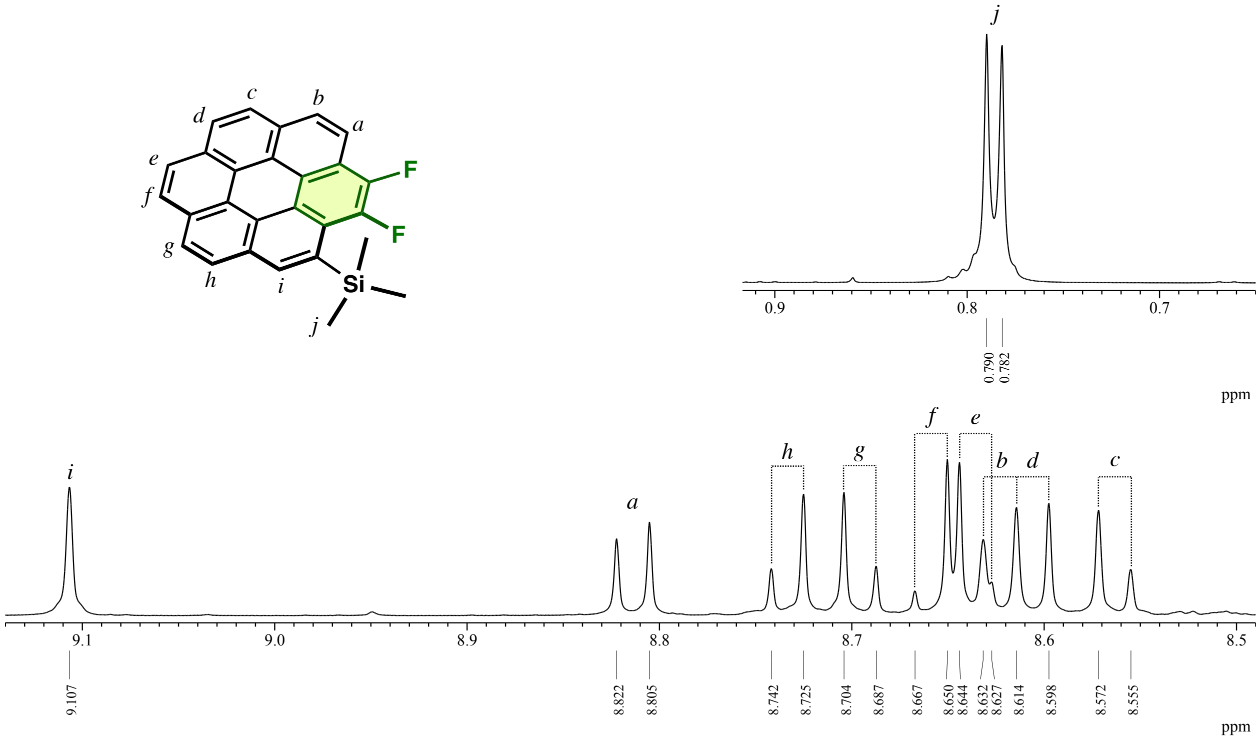

Figure S20. Enlarged ^{1}H NMR spectrum (500 MHz, $CDCl_3$, 298 K) of **TMS-2**.

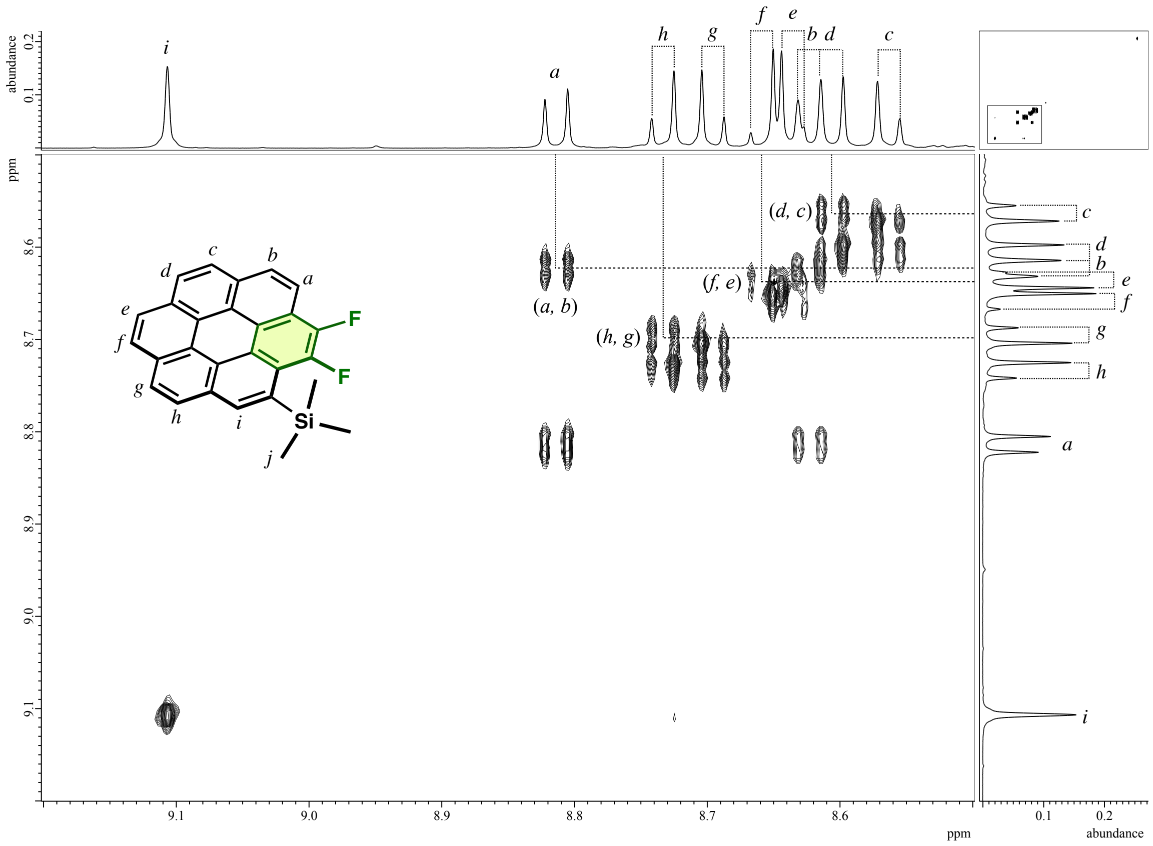

Figure S21. ^1H–^1H COSY NMR spectrum (500 MHz, $CDCl_3$, 298 K) of **TMS-2**.

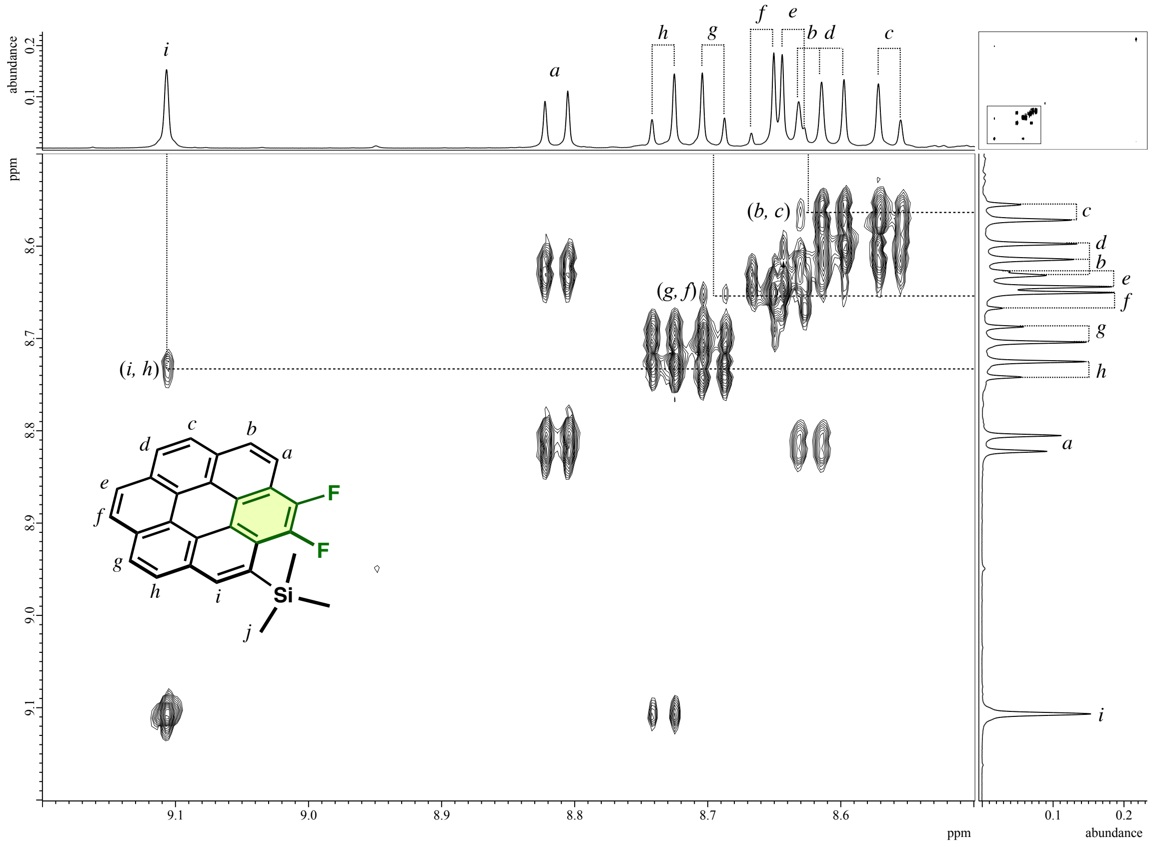

Figure S22. ^1H–^1H long-range COSY NMR spectrum (500 MHz, $CDCl_3$, 298 K) of **TMS-2**.

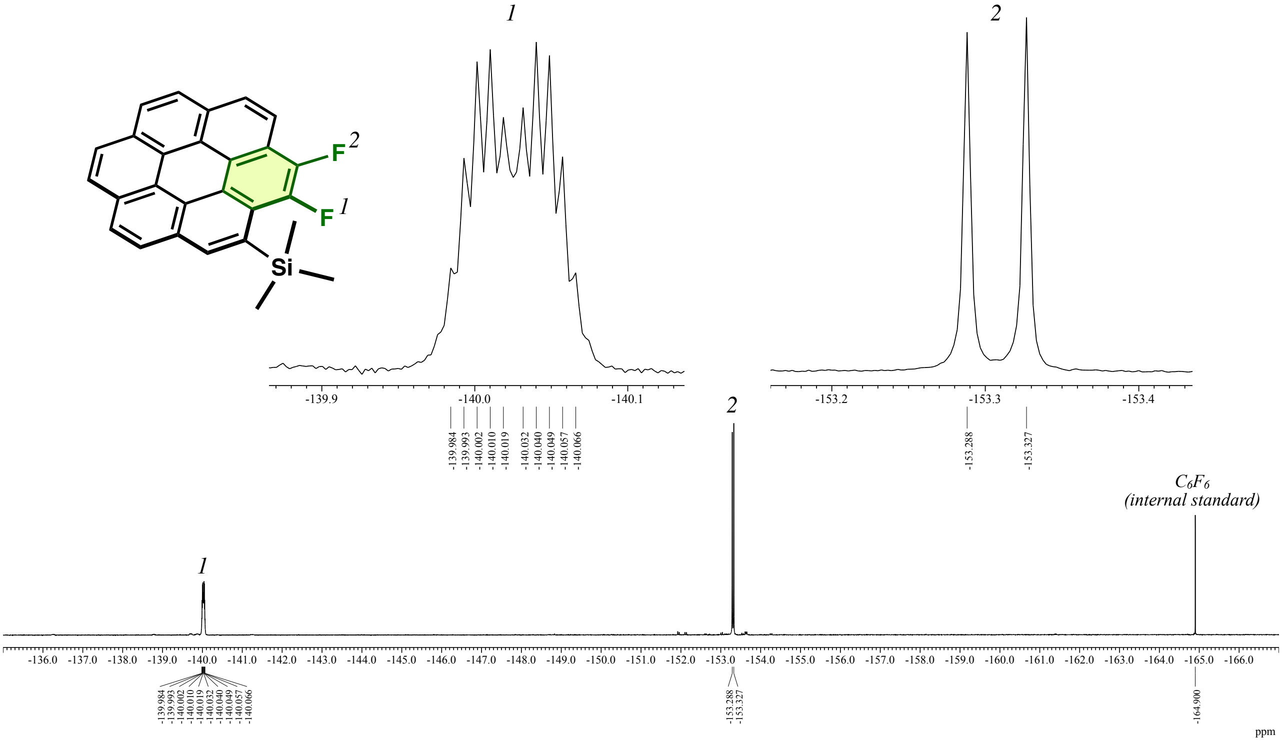

Figure S23. ^{19}F NMR spectrum (470 MHz, $CDCl_3$, 298 K) of **TMS-2**.

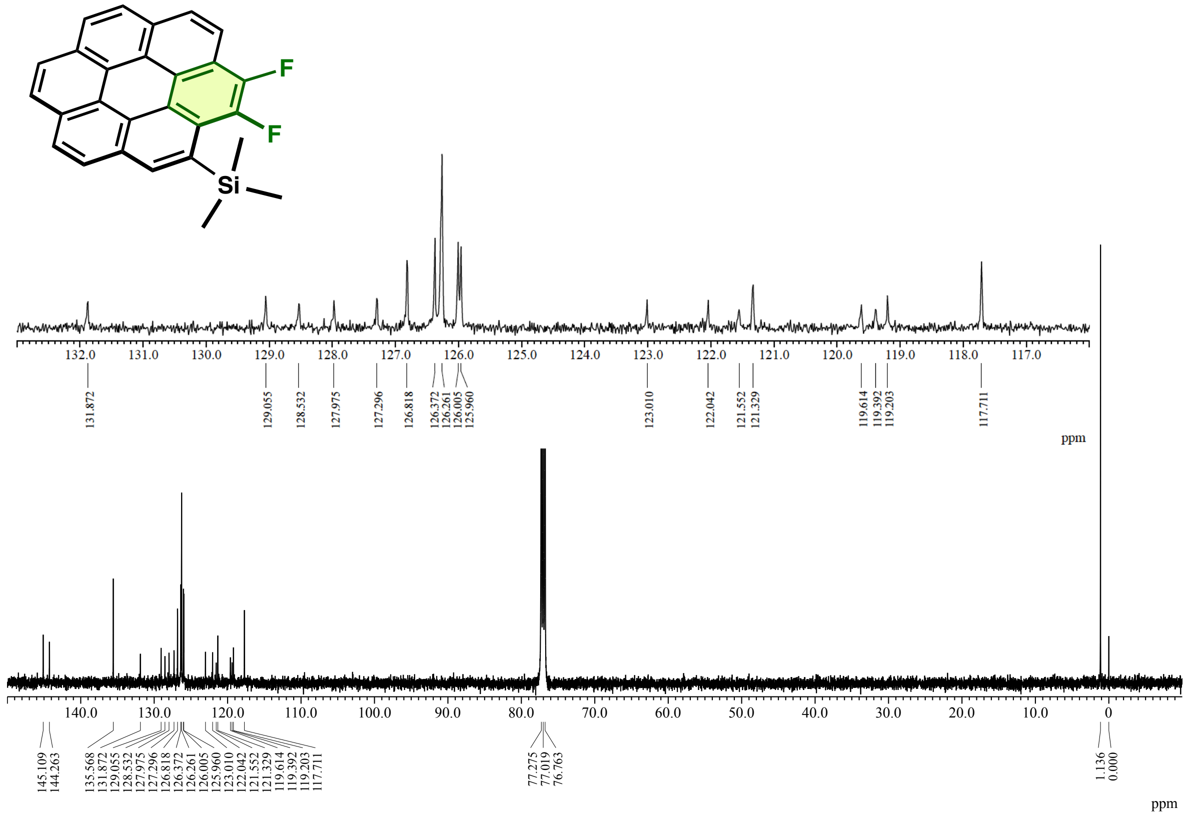

Figure S24. $^{13}C\{^{1}H, ^{19}F\}$ NMR spectrum (125 MHz, $CDCl_3$, 298 K) of **TMS-2**.

Mes-2

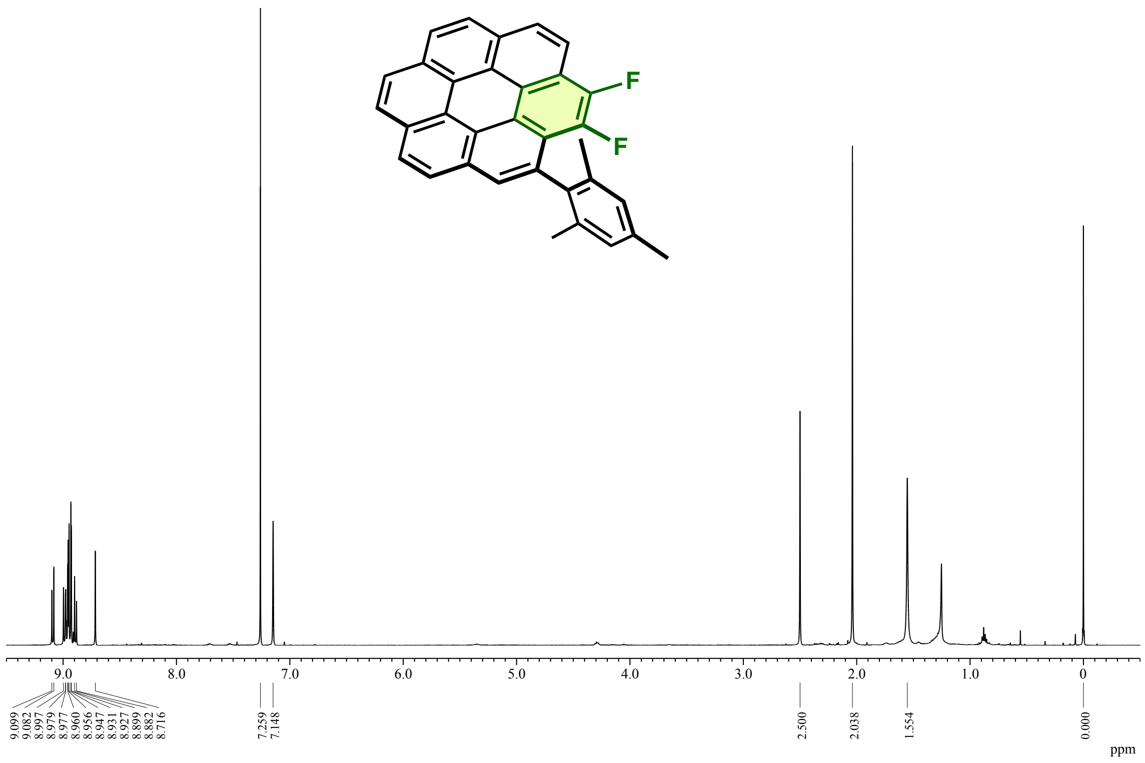

Figure S25. ^{1}H NMR spectrum (500 MHz, $CDCl_3$, 298 K) of **Mes-2**.

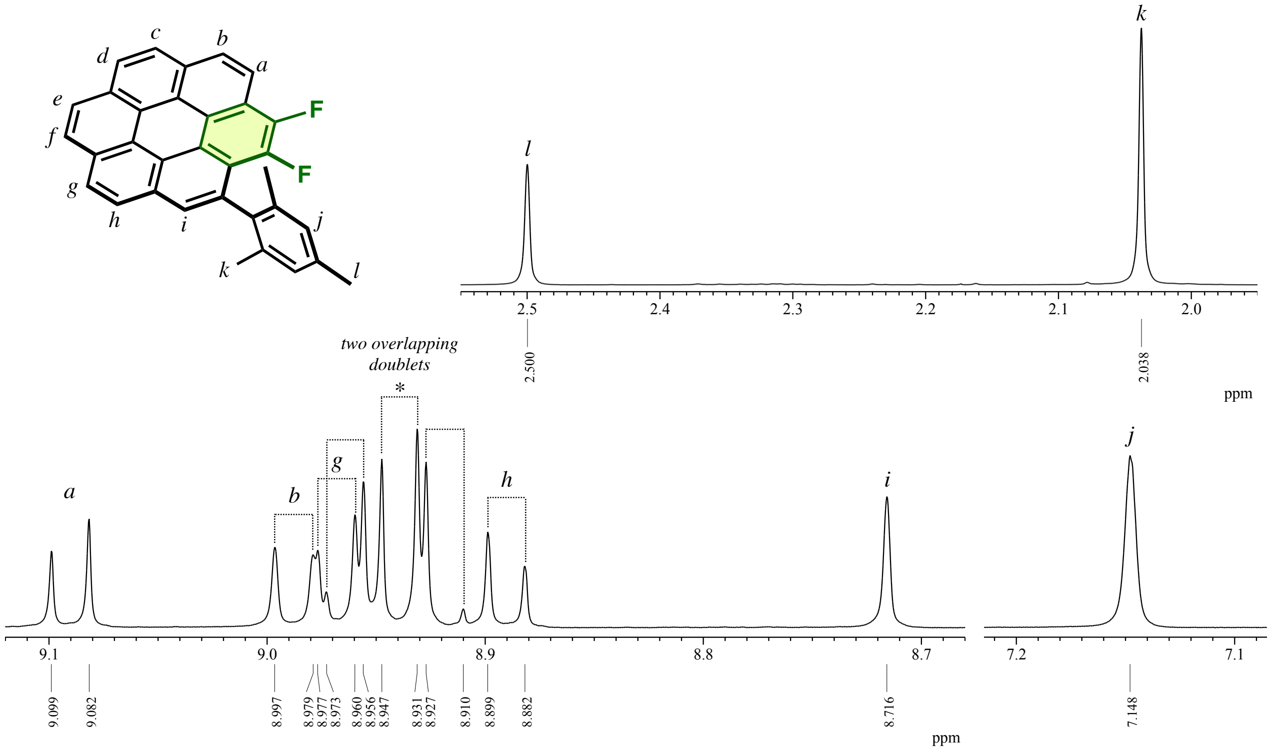

Figure S26. Enlarged ^{1}H NMR spectrum (500 MHz, $CDCl_3$, 298 K) of **Mes-2**.

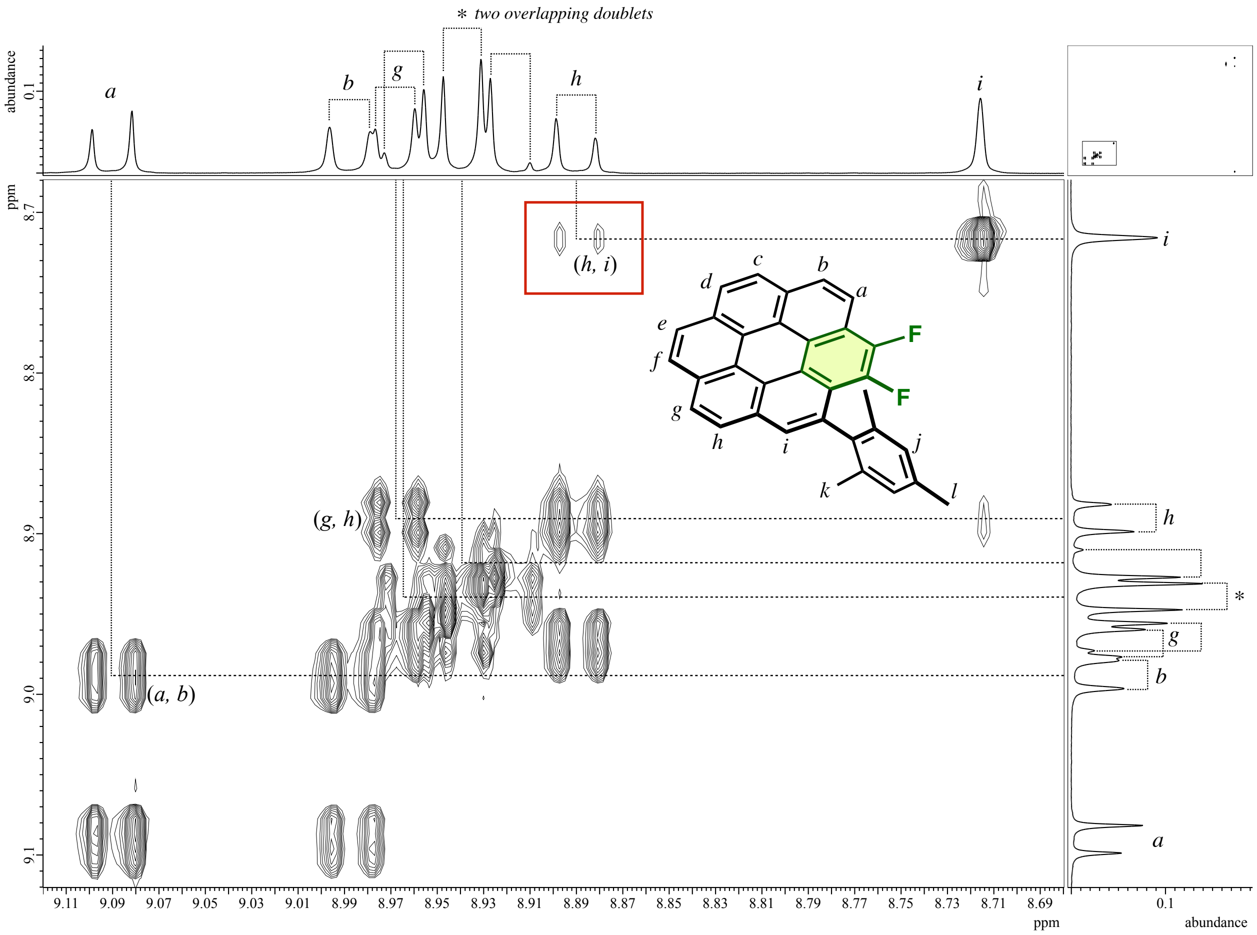

Figure S27. ^1H–^1H COSY NMR spectrum (500 MHz, $CDCl_3$, 298 K) of **Mes-2**. The long-range correlation between the signals H_h and H_i in the COSY NMR spectrum, along with the exclusive downfield shift of signal H_a, confirms the position of the mesityl group on the F_2-coronene framework.

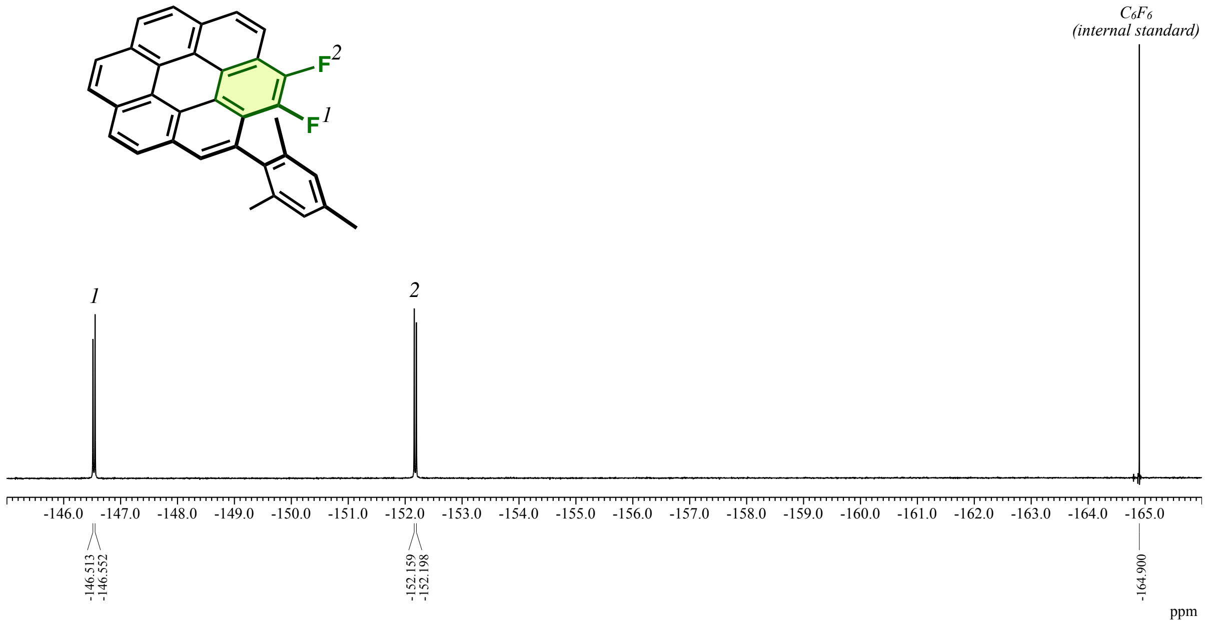

Figure S28. ^{19}F NMR spectrum (470 MHz, $CDCl_3$, 298 K) of **Mes-2**.

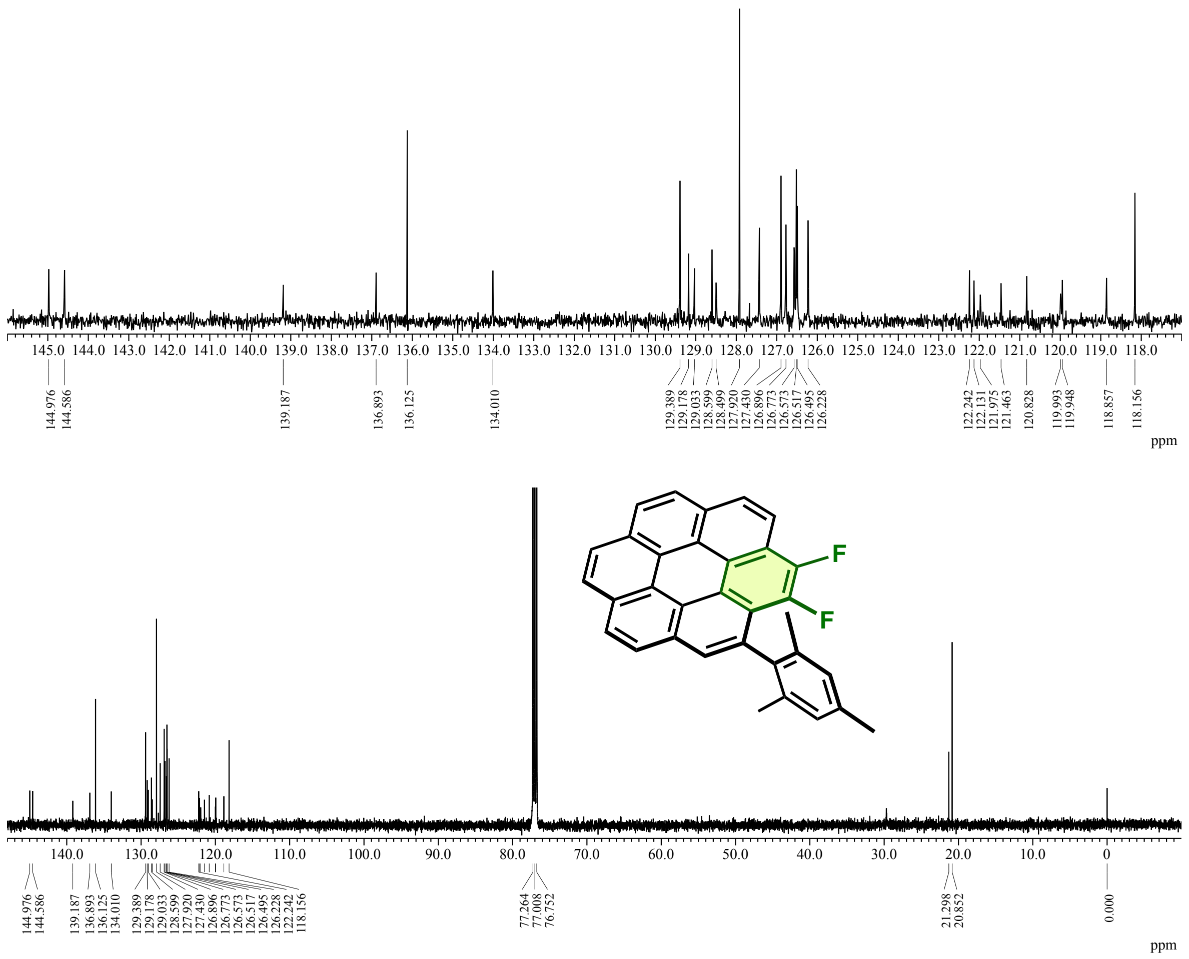

Figure S29. $^{13}C\{^{1}H, ^{19}F\}$ NMR spectrum (125 MHz, $CDCl_3$, 298 K) of **Mes-2**.

TMS-1d

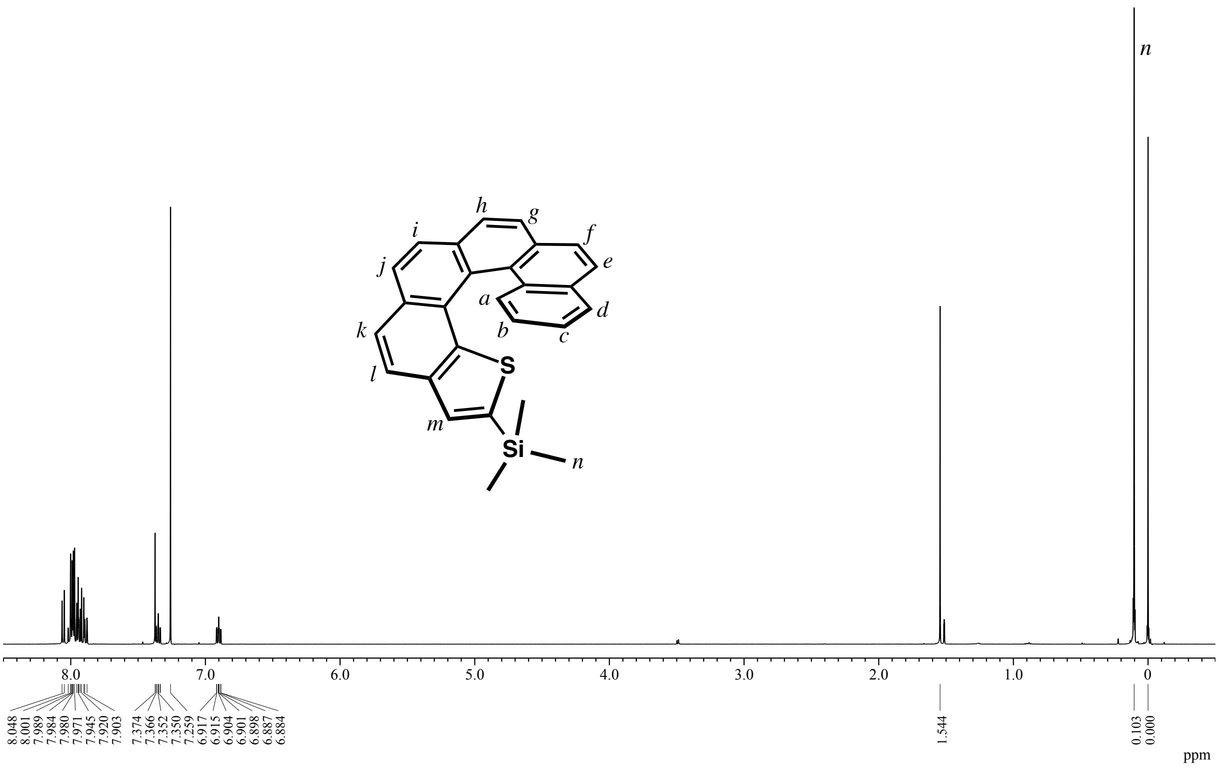

Figure S30. ^{1}H NMR spectrum (500 MHz, $CDCl_3$, 298 K) of **TMS-1d**.

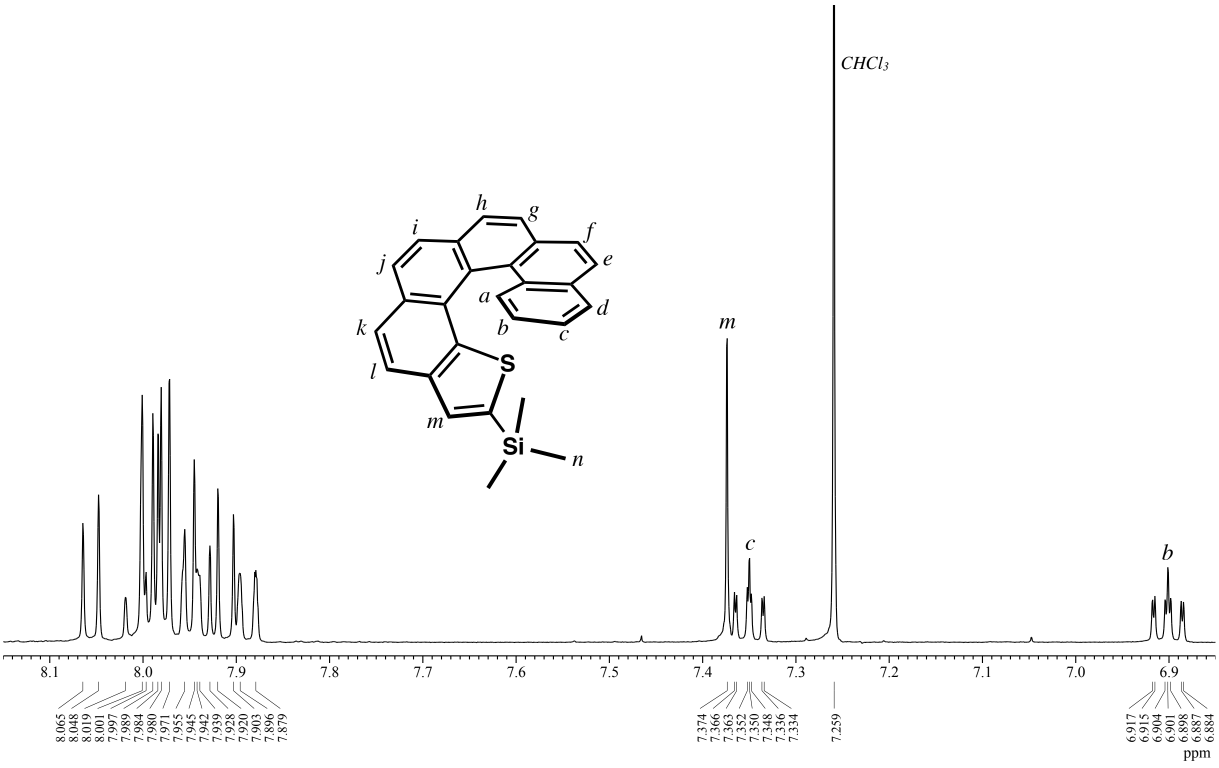

Figure S31. Enlarged ^{1}H NMR spectrum (500 MHz, $CDCl_3$, 298 K) of **TMS-1d**.

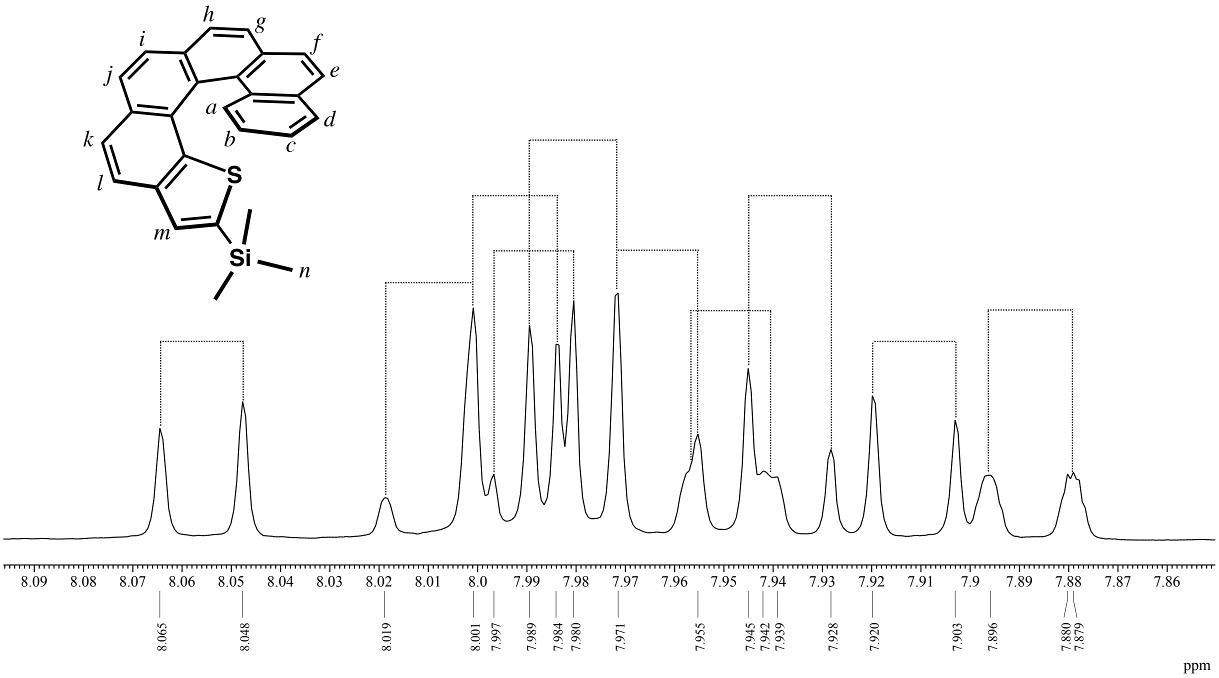

Figure S32. Enlarged ^{1}H NMR spectrum (500 MHz, $CDCl_3$, 298 K) of **TMS-1d**. Although unambiguous signal assignment was not possible due to extensive overlap, the spectrum was carefully divided into regions based on peak shape and intensity, as indicated by dotted lines.

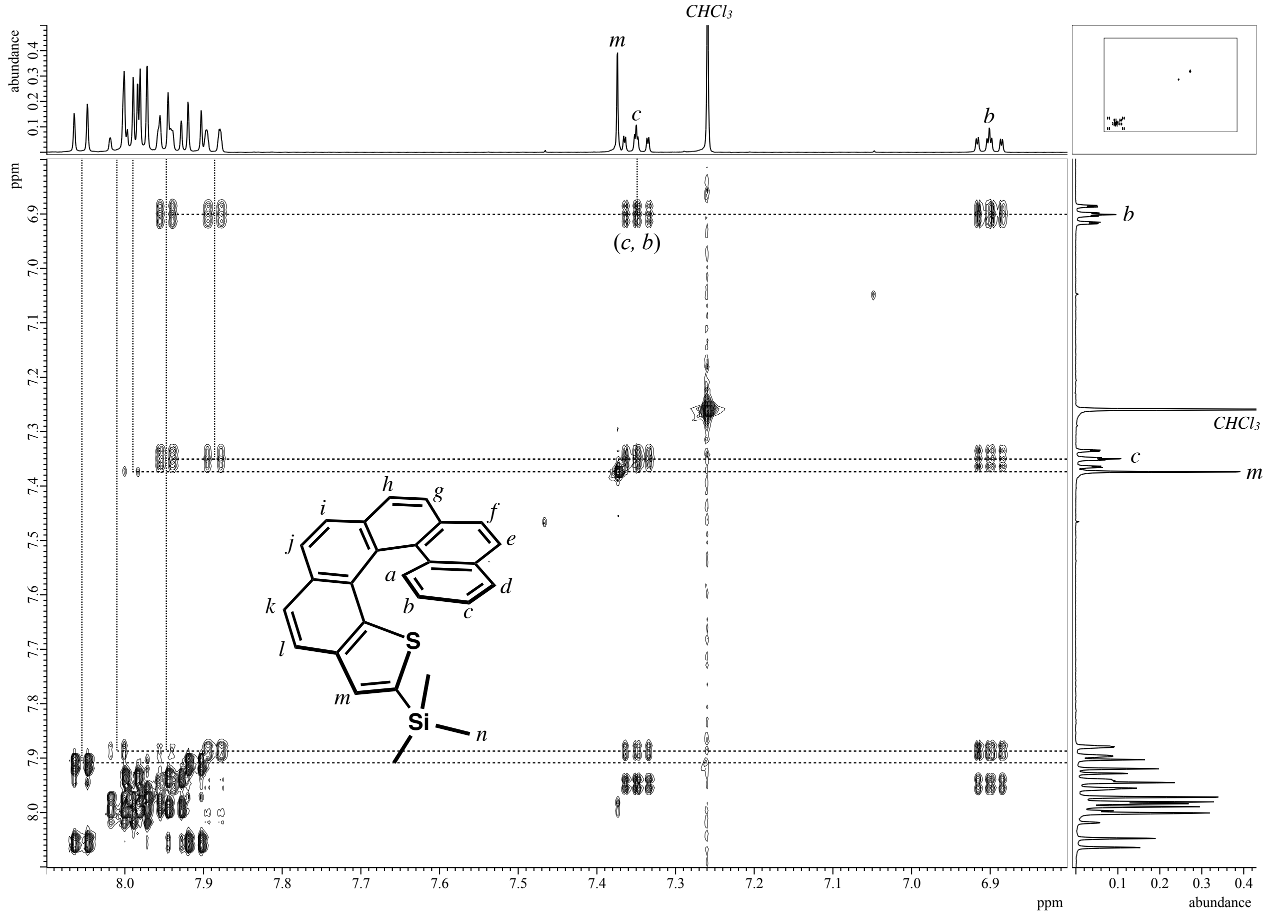

Figure S33. ^{1}H–^{1}H COSY NMR spectrum (500 MHz, $CDCl_3$, 298 K) of **TMS-1d**.

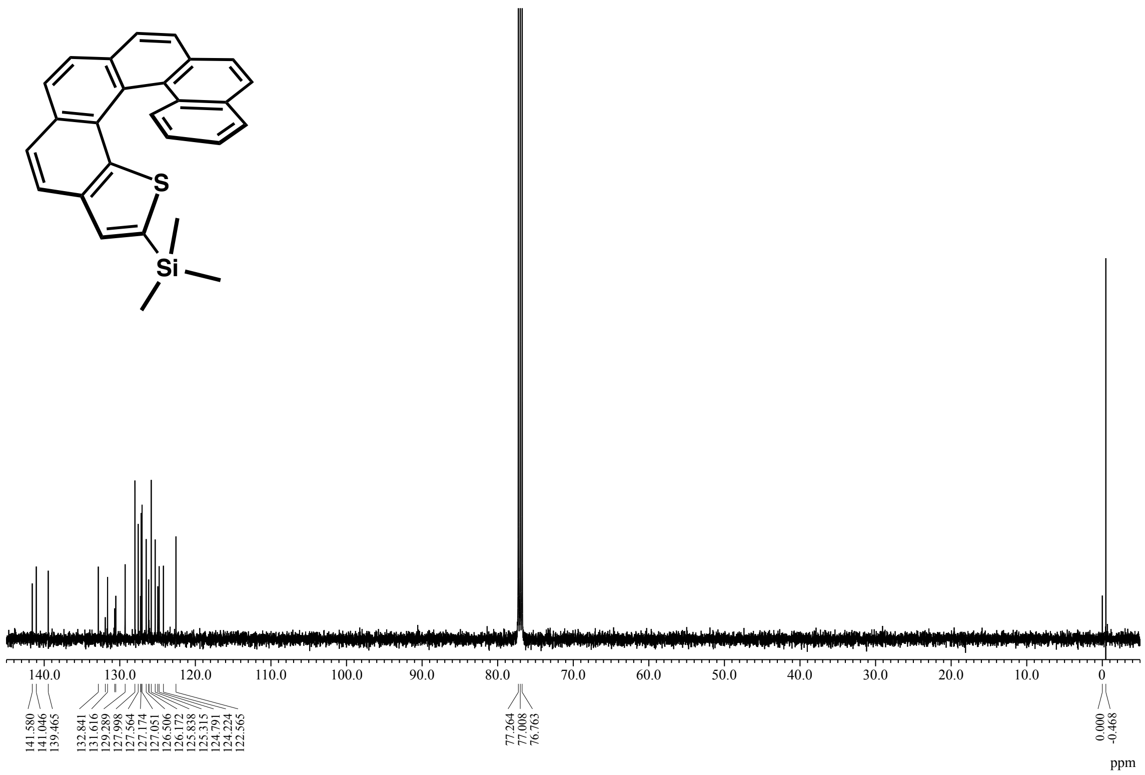

Figure S34. $^{13}C\{^{1}H\}$ NMR spectrum (125 MHz, $CDCl_3$, 298 K) of **TMS-1d**.

TMS-1e

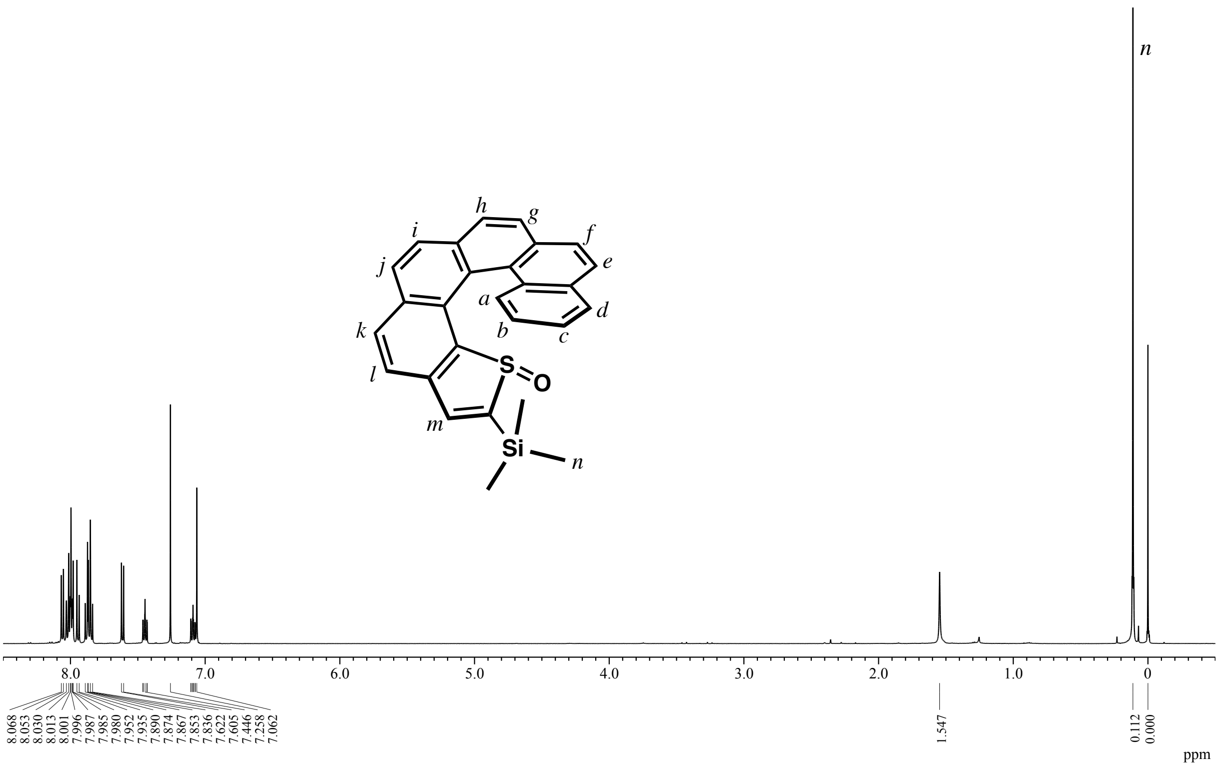

Figure S35. ^{1}H NMR spectrum (500 MHz, $CDCl_3$, 298 K) of **TMS-1e**.

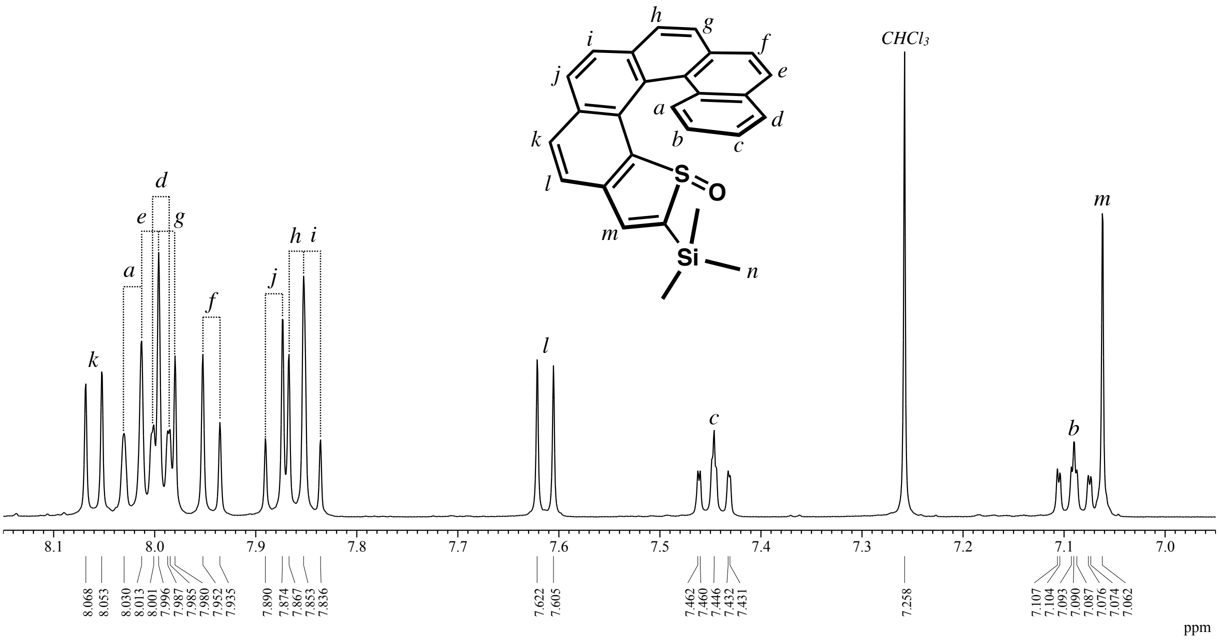

Figure S36. Enlarged ^{1}H NMR spectrum (500 MHz, $CDCl_3$, 298 K) of **TMS-1e**.

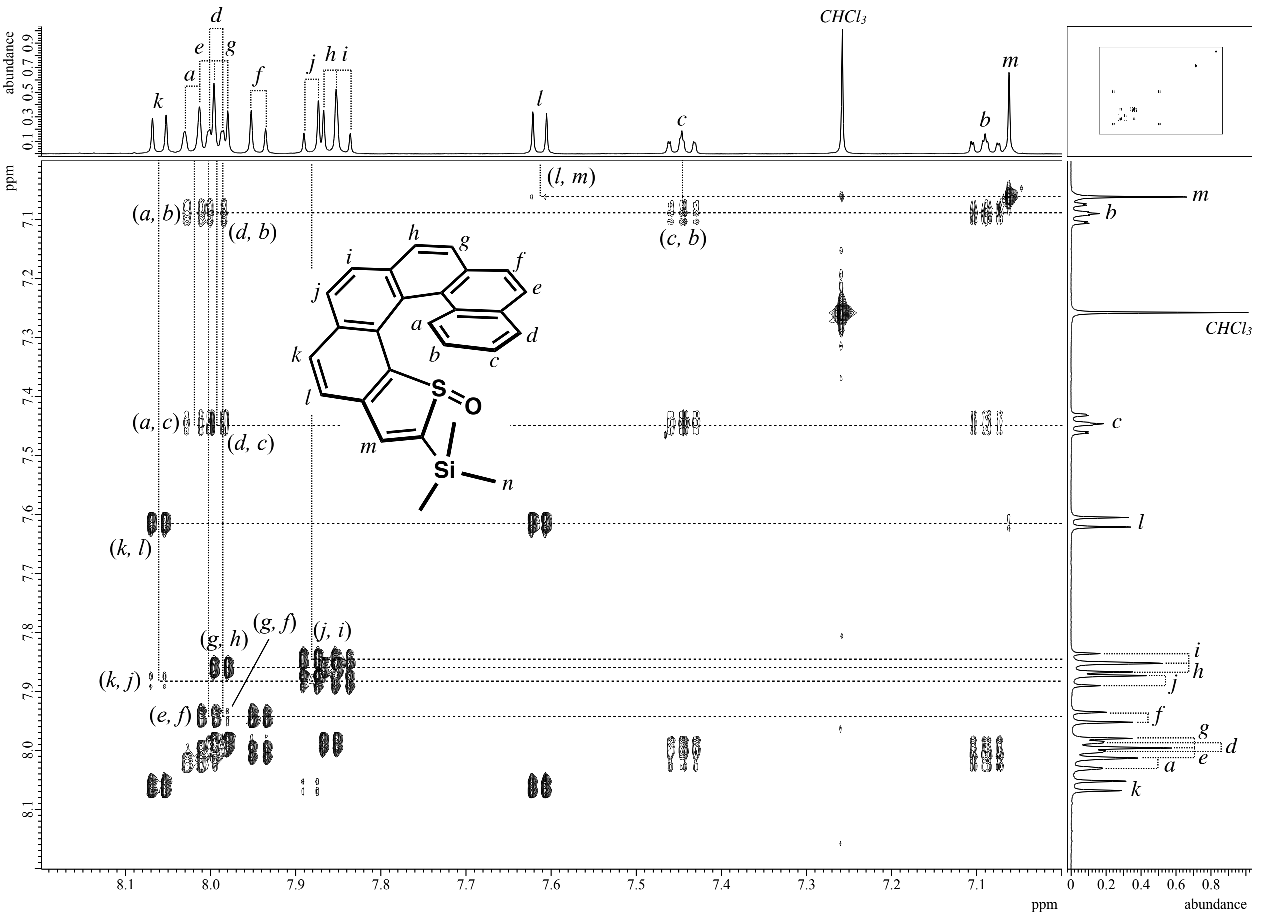

Figure S37. ^{1}H–^{1}H COSY NMR spectrum (500 MHz, $CDCl_3$, 298 K) of **TMS-1e**.

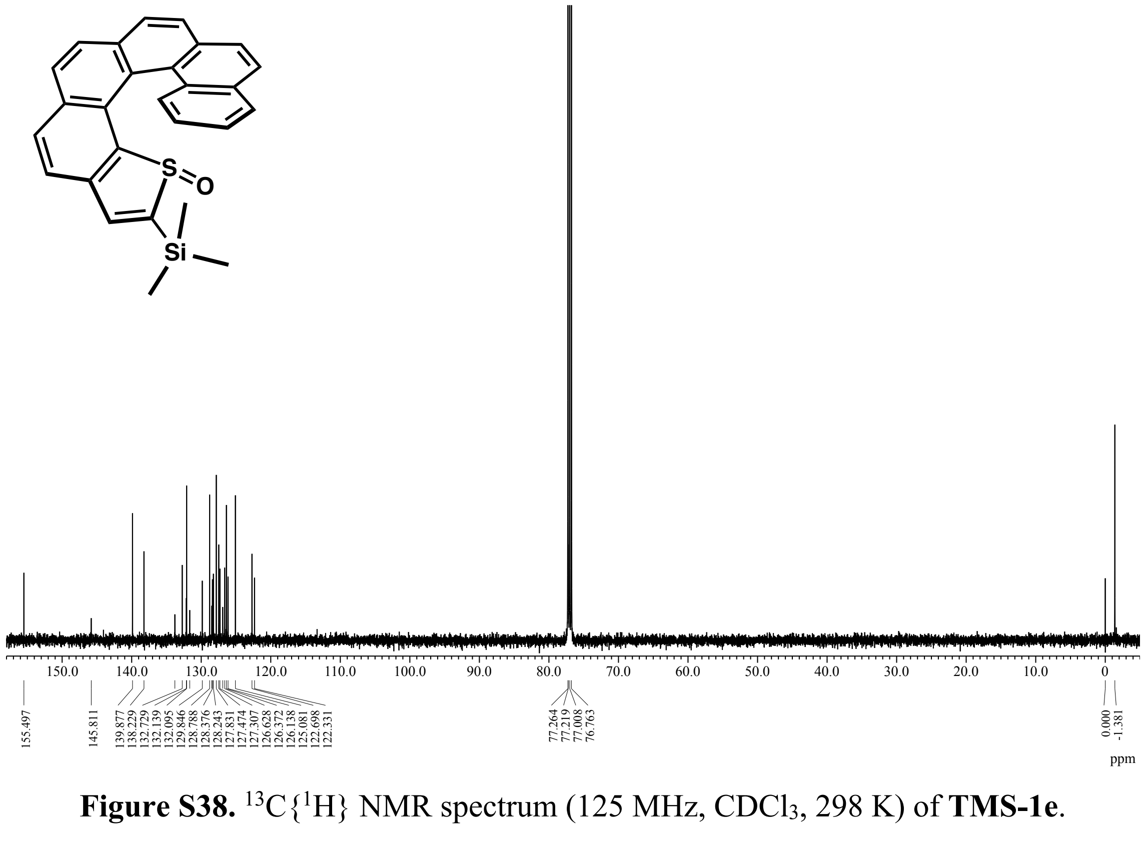

Figure S38. $^{13}C\{^1H\}$ NMR spectrum (125 MHz, $CDCl_3$, 298 K) of **TMS-1e**.

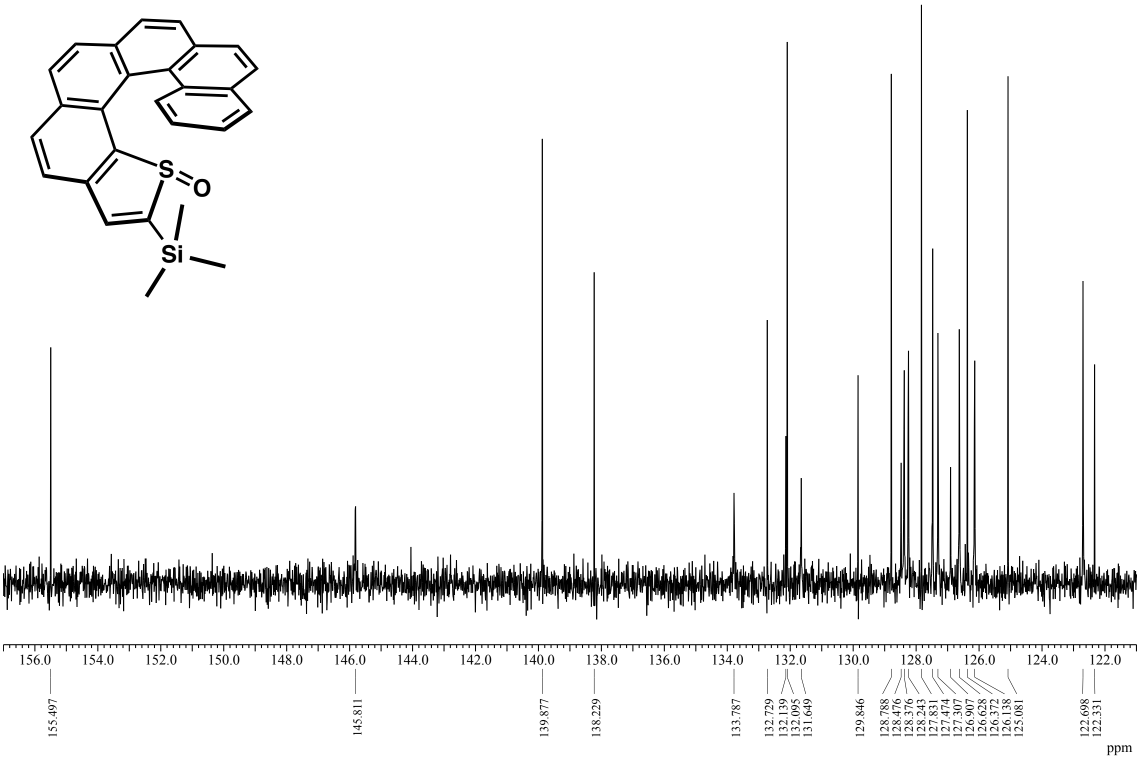

Figure S39. Enlarged $^{13}C\{^1H\}$ NMR spectrum (125 MHz, $CDCl_3$, 298 K) of **TMS-1e**.

TMS-coronene

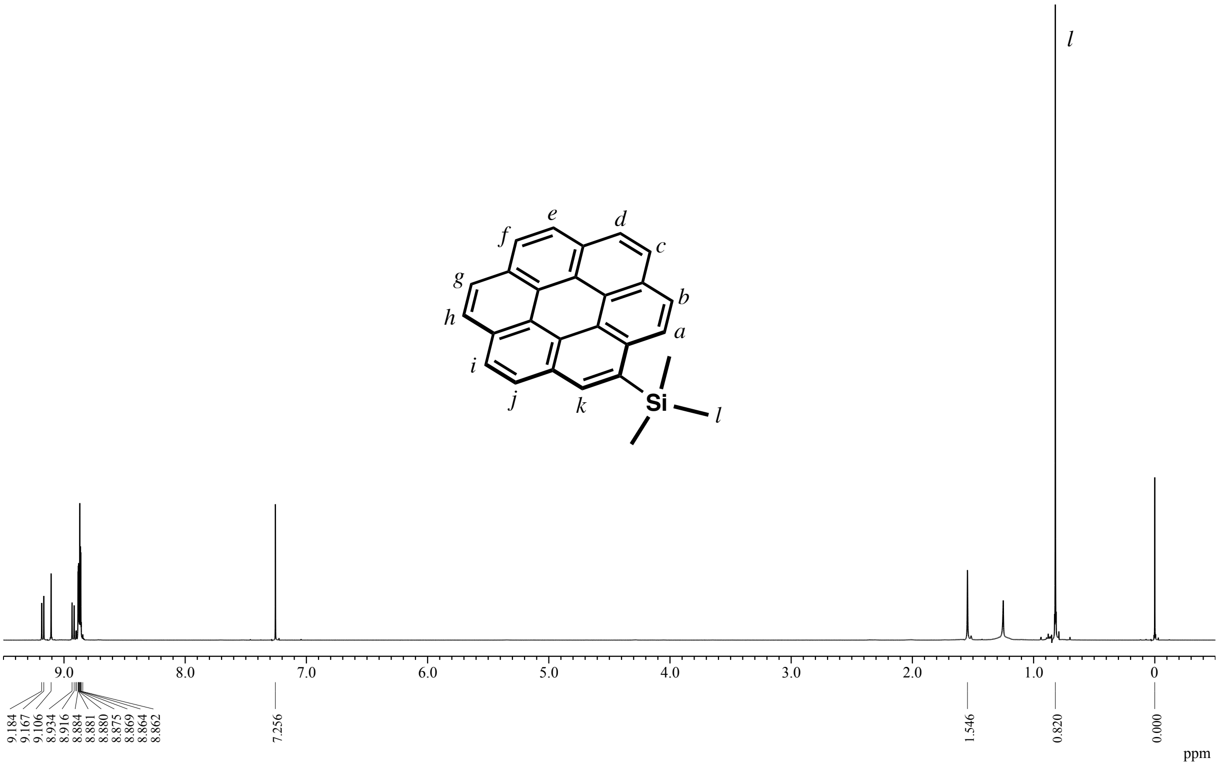

Figure S40. ^{1}H NMR spectrum (500 MHz, $CDCl_3$, 298 K) of **TMS-coronene**.

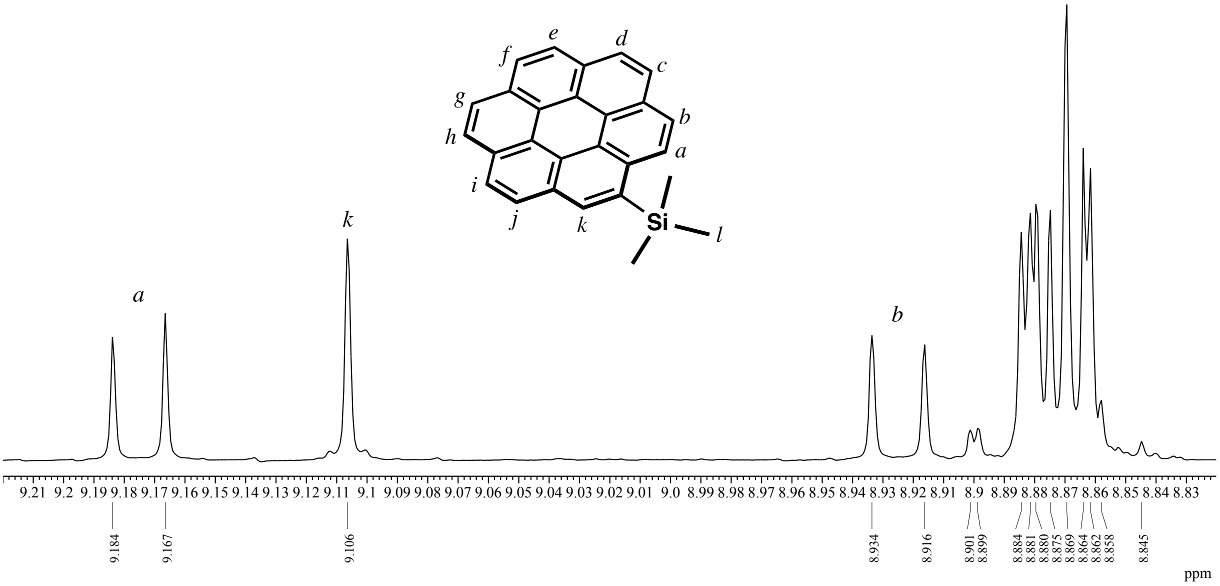

Figure S41. Enlarged ^{1}H NMR spectrum (500 MHz, $CDCl_3$, 298 K) of **TMS-coronene**.

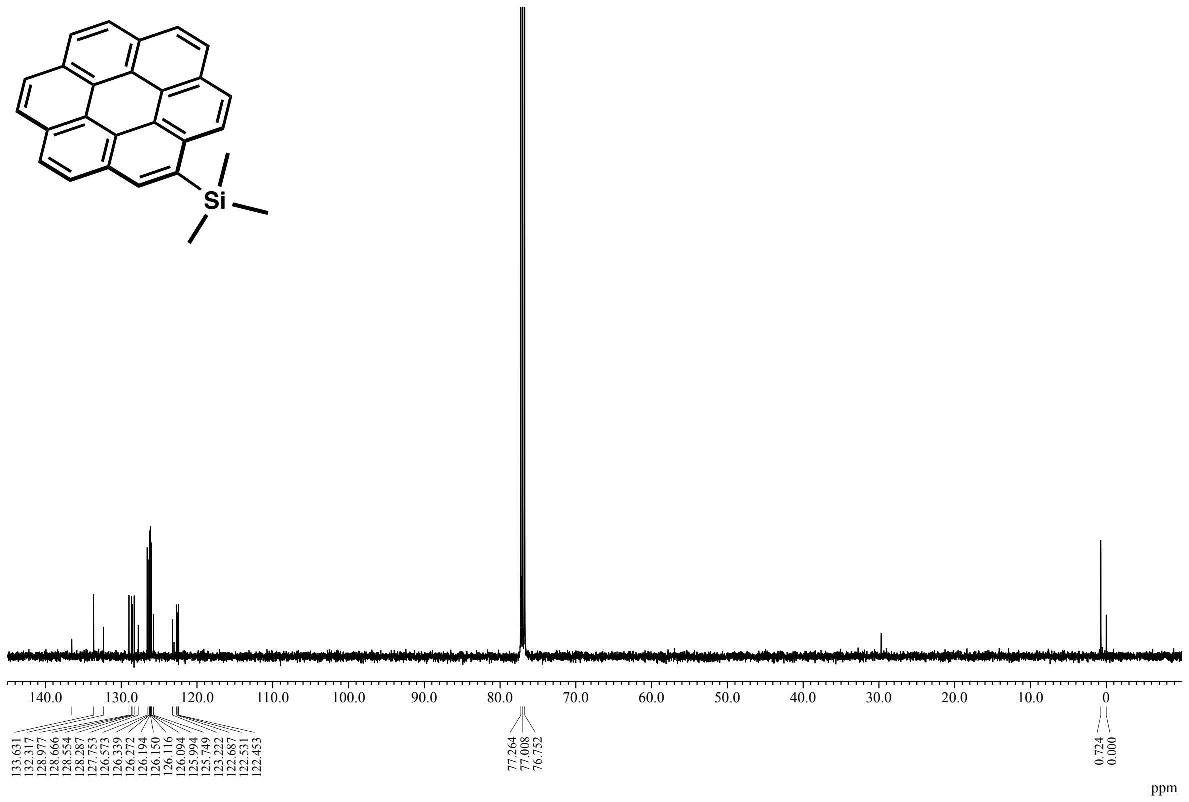

Figure S42. $^{13}C\{^1H\}$ NMR spectrum (125 MHz, $CDCl_3$, 298 K) of **TMS-coronene**.

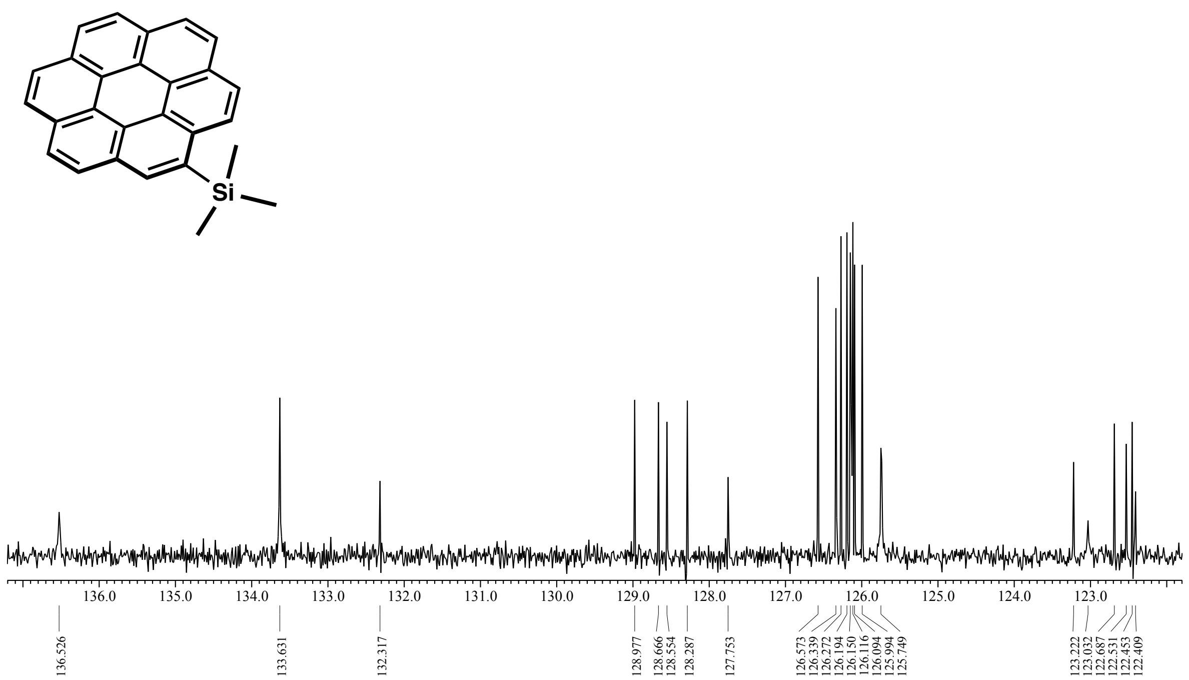

Figure S43. Enlarged $^{13}C\{^1H\}$ NMR spectrum (125 MHz, $CDCl_3$, 298 K) of **TMS-coronene**.

6. MS spectra

TMS-1a

TDCMAS ESI-TOF

Analysis Info

Analysis Name	D:\Data\ofcbunseki\irai\2024\aliance\murase\241216\MRS-044-000002.d	Acquisition Date	2024/12/13 9:30:59
Method	esi_posi_low.m	Operator	BDAL@DE
Sample Name	MRS-044-	Instrument / Ser#	micrOTOF 213750.10321
Comment			

Acquisition Parameter

Source Type	ESI	Ion Polarity	Positive	Set Nebulizer	0.3 Bar
Focus	Not active			Set Dry Heater	180 ‹C
Scan Begin	50 m/z	Set Capillary	4500 V	Set Dry Gas	4.0 l/min
Scan End	1300 m/z	Set End Plate Offset	-500 V	Set Divert Valve	Waste

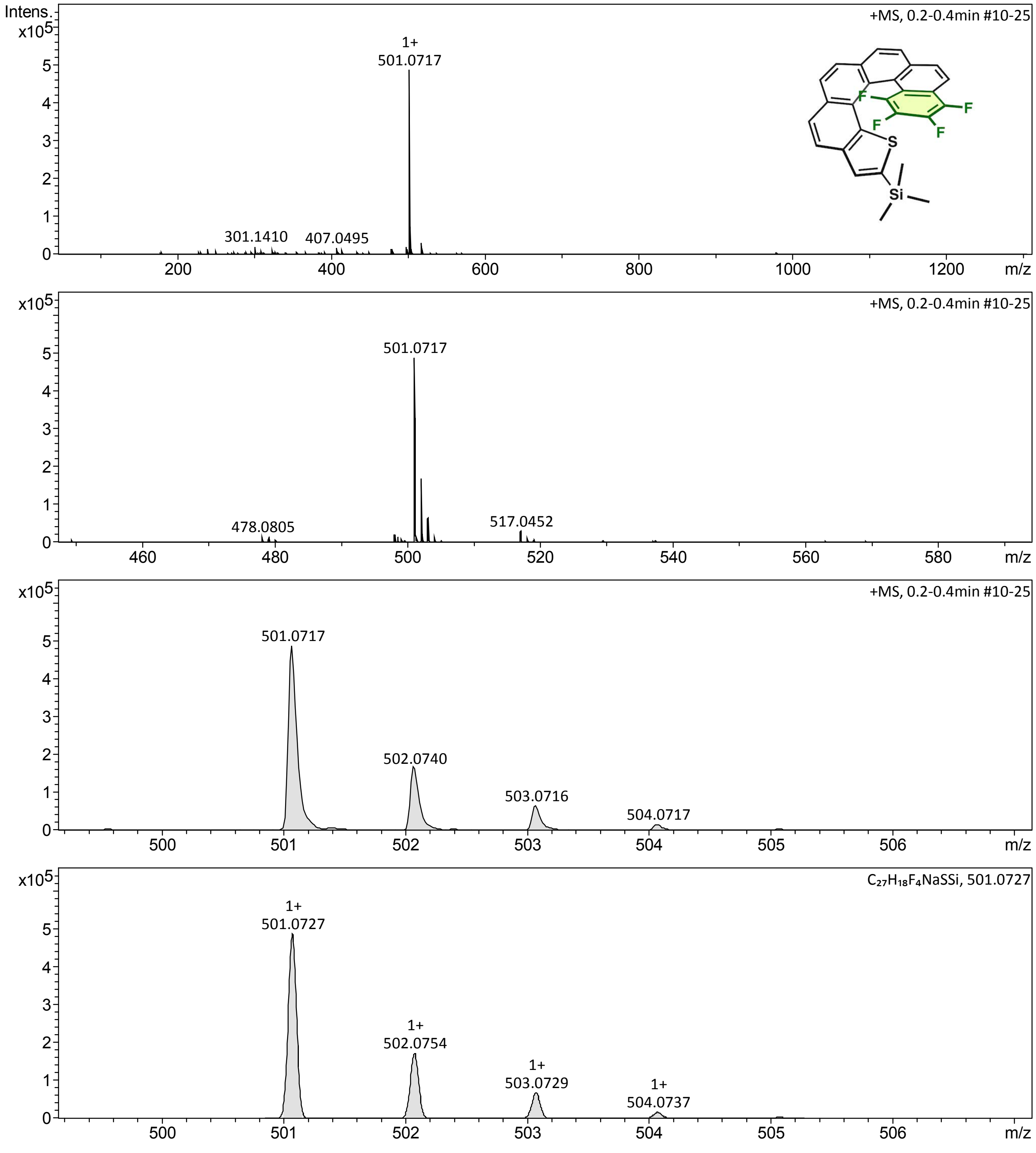

TMS-1b

TDCMAS ESI-TOF

Analysis Info

Analysis Name	D:\Data\ofcbunseki\irai\2024\aliance\murase\241216\MRS-045-000001.d	Acquisition Date	2024/12/13 9:38:55	
Method	esi_posi_low.m	Operator	BDAL@DE	
Sample Name	MRS-045-	Instrument / Ser#	micrOTOF	213750.10321
Comment				

Acquisition Parameter

Source Type	ESI	Ion Polarity	Positive	Set Nebulizer	0.3 Bar
Focus	Not active			Set Dry Heater	180 ‹C
Scan Begin	50 m/z	Set Capillary	4500 V	Set Dry Gas	4.0 l/min
Scan End	1300 m/z	Set End Plate Offset	-500 V	Set Divert Valve	Waste

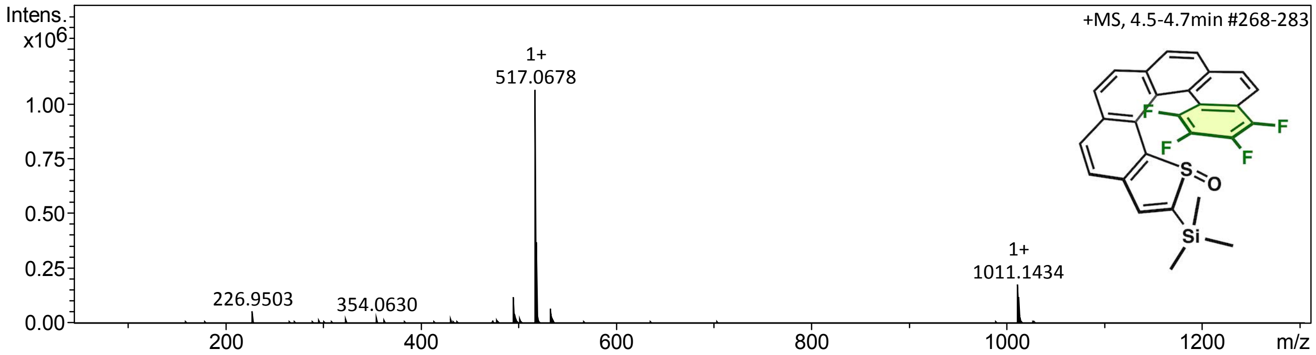

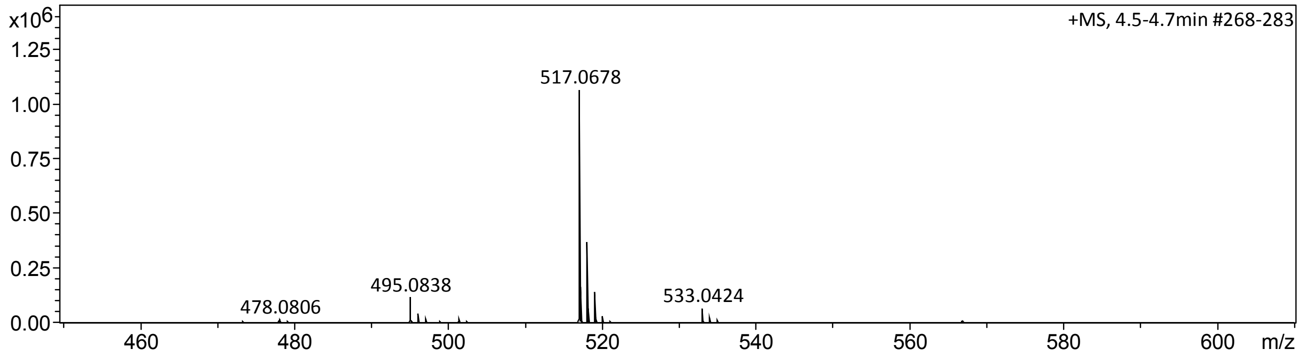

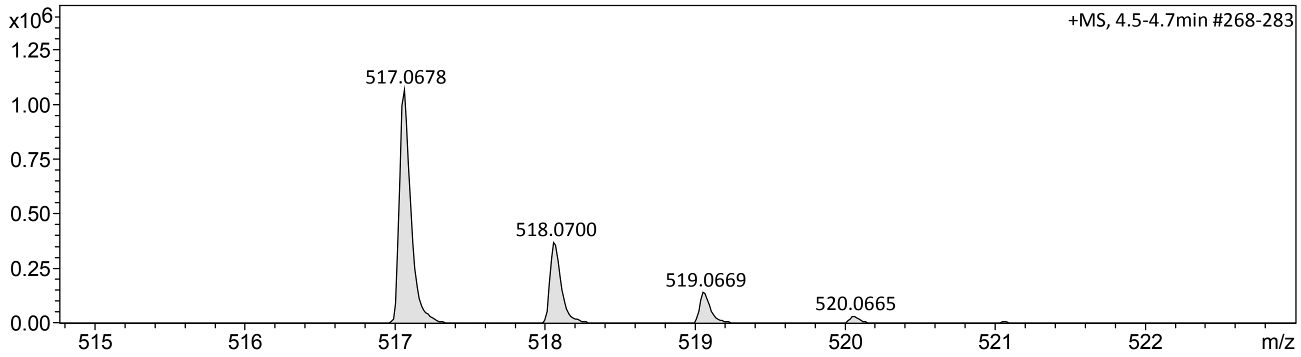

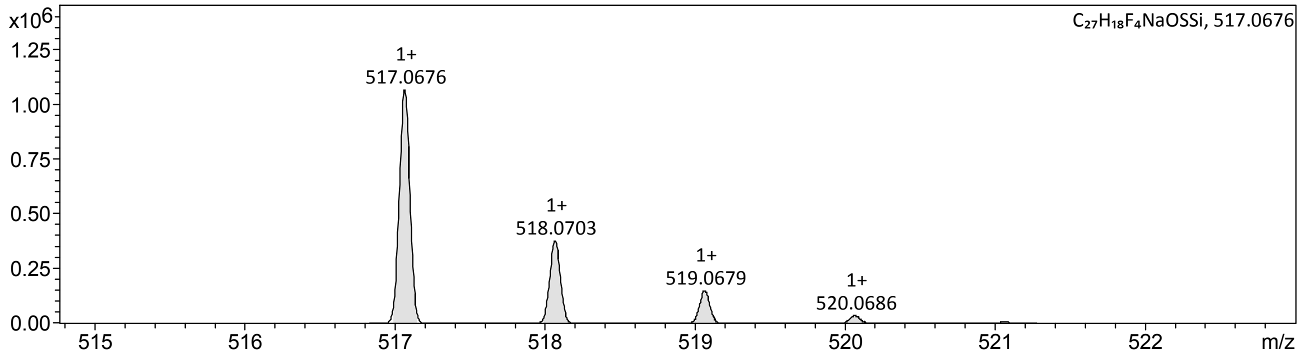

TMS-1c

TDCMAS ESI-TOF

Analysis Info

Analysis Name	D:\Data\ofcbunseki\irai\2024\aliance\murase\241216\MRS-046-000003.d	Acquisition Date	2024/12/13 9:57:08
Method	esi_posi_low.m	Operator	BDAL@DE
Sample Name	MRS-046-	Instrument / Ser#	micrOTOF 213750.10321
Comment			

Acquisition Parameter

Source Type	ESI	Ion Polarity	Positive	Set Nebulizer	0.3 Bar
Focus	Not active			Set Dry Heater	180 ‹C
Scan Begin	50 m/z	Set Capillary	4500 V	Set Dry Gas	4.0 l/min
Scan End	1300 m/z	Set End Plate Offset	-500 V	Set Divert Valve	Waste

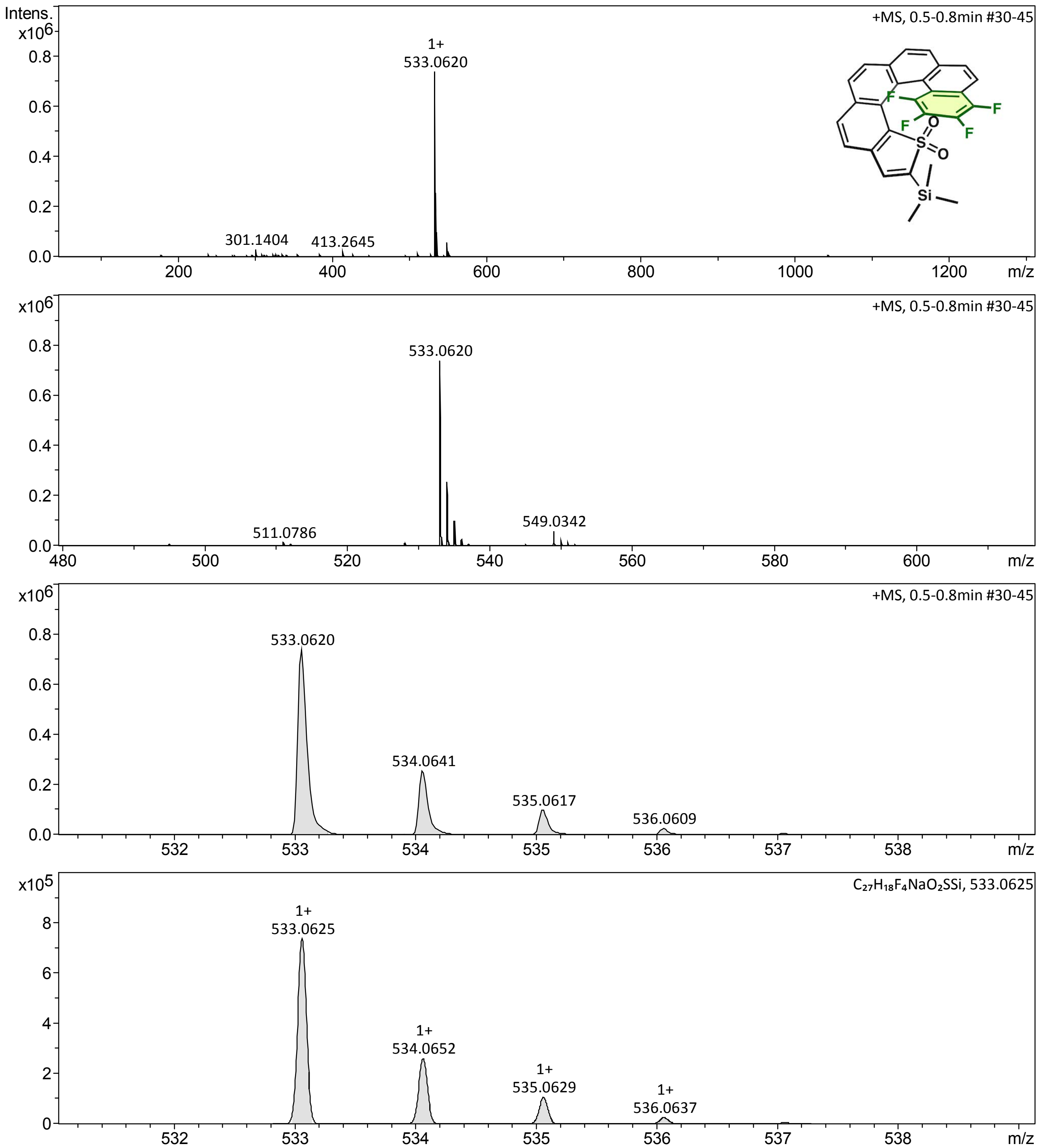

TMS-2

TDCMAS ESI-TOF

Analysis Info

		Acquisition Date	2024/12/13 10:36:54
Analysis Name	D:\Data\ofcbunseki\irai\2024\aliance\murase\241216\MRS-047-APCI-000003.d		
Method	APCI_II_pos_microTOF.m	Operator	BDAL@DE
Sample Name	MRS-047-	Instrument / Ser#	micrOTOF 213750.10321
Comment			

Acquisition Parameter

Source Type	APCI	Ion Polarity	Positive	Set Nebulizer	2.0 Bar
Focus	Not active			Set Dry Heater	200 ‹C
Scan Begin	50 m/z	Set Capillary	4500 V	Set Dry Gas	3.5 l/min
Scan End	2000 m/z	Set End Plate Offset	-500 V	Set Divert Valve	Waste

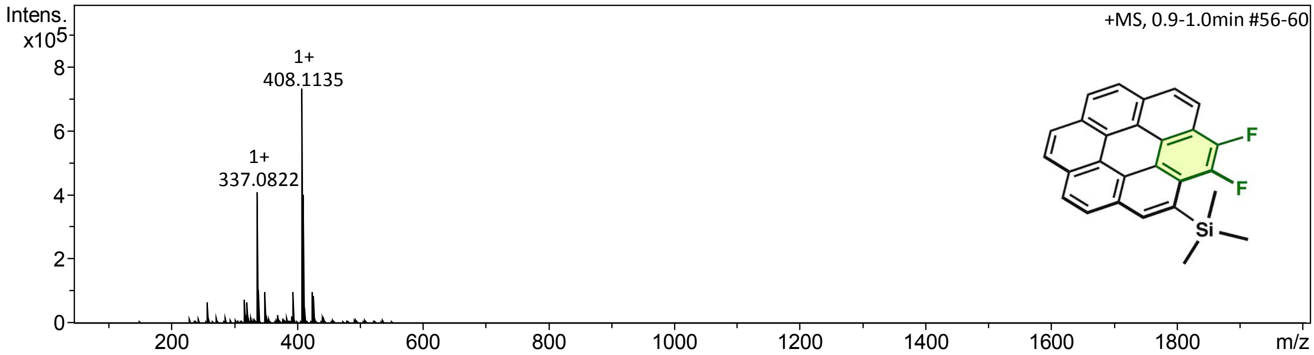

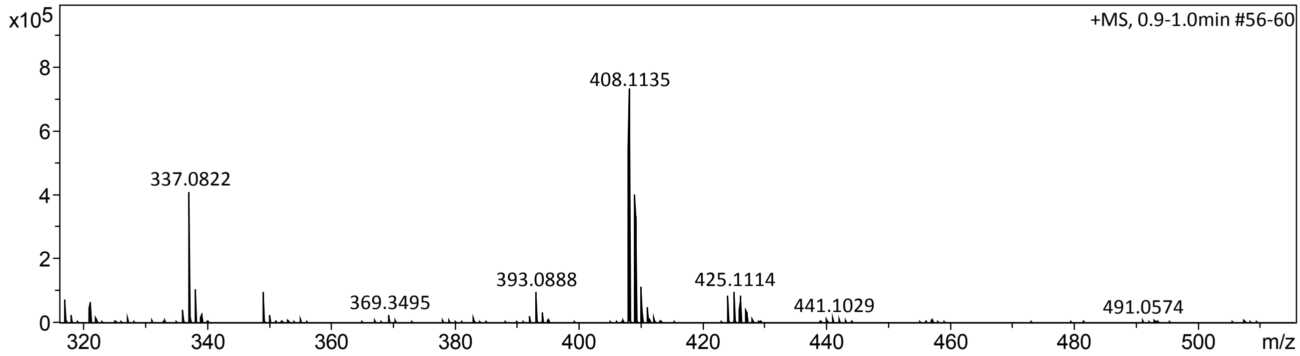

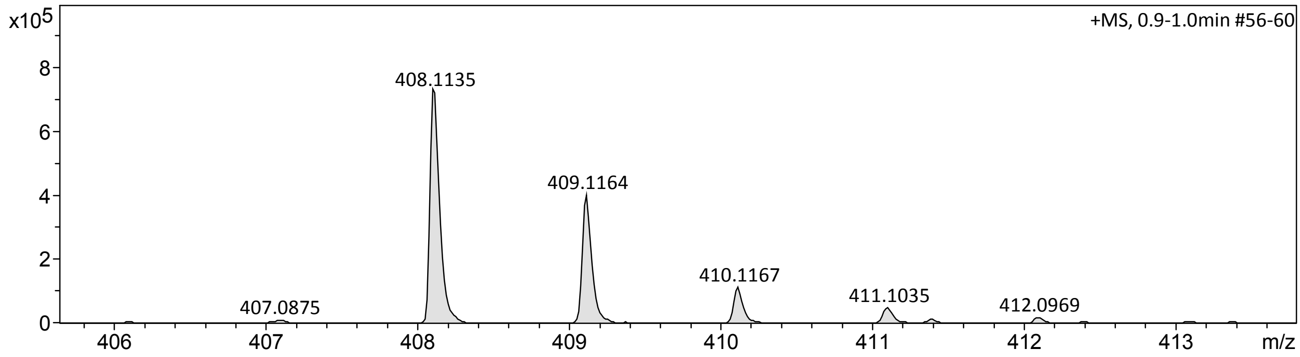

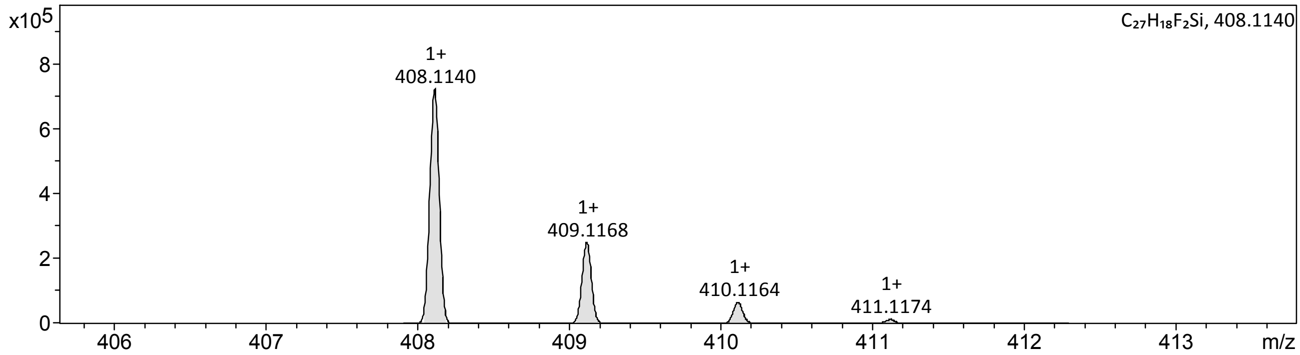

Mes-2

TDCMAS ESI-TOF

Analysis Info

Analysis Name	D:\Data\ofcbunseki\irai\2025\aliance\250513murase\MRS-048-APCI-000002.d	Acquisition Date	2025/05/13 11:03:34
Method	APCI_II_pos_microTOF.m	Operator	BDAL@DE
Sample Name	MRS-048-APCI-	Instrument / Ser#	micrOTOF 213750.10321
Comment			

Acquisition Parameter

Source Type	APCI	Ion Polarity	Positive	Set Nebulizer	2.0 Bar
Focus	Not active			Set Dry Heater	200 ‹C
Scan Begin	50 m/z	Set Capillary	4500 V	Set Dry Gas	3.5 l/min
Scan End	2000 m/z	Set End Plate Offset	-500 V	Set Divert Valve	Waste

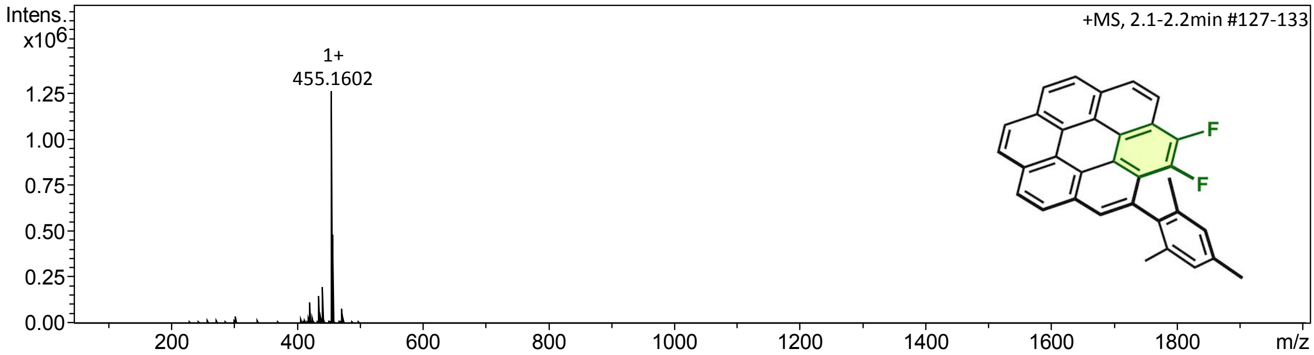

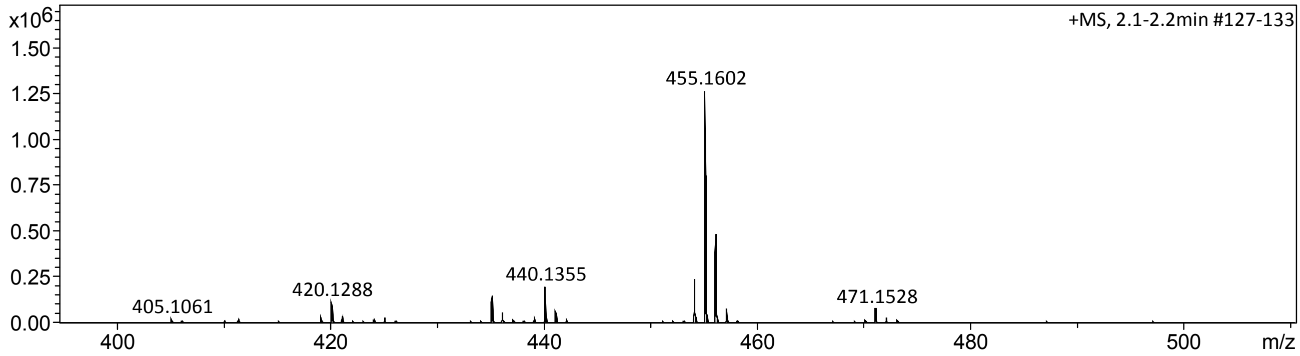

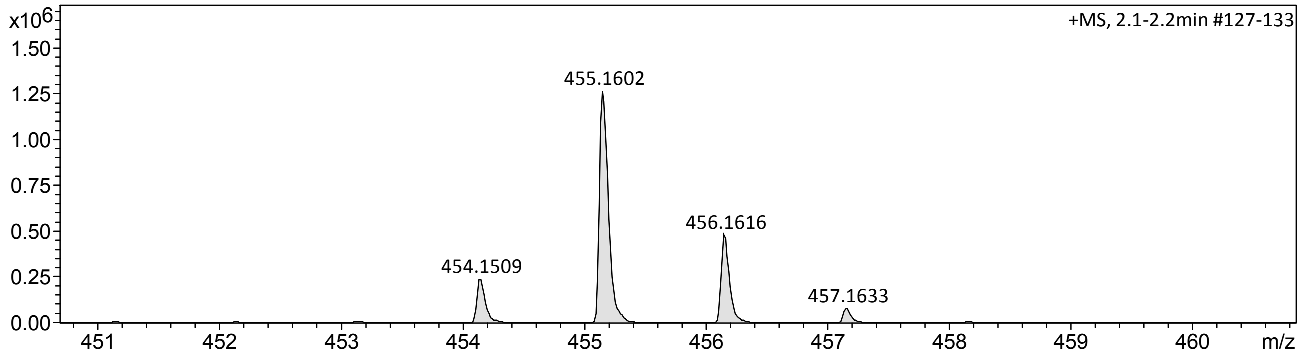

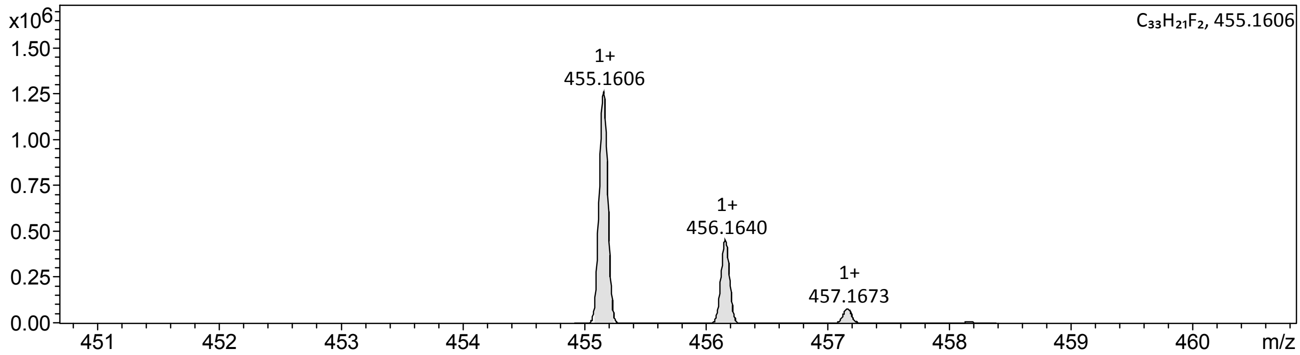

TMS-1d

TDCMAS ESI-TOF

Analysis Info

Analysis Name	D:\Data\ofcbunseki\irai\2025\aliance\250513murase\MRS-049-000002.d	Acquisition Date	2025/05/12 11:12:35
Method	check.m	Operator	BDAL@DE
Sample Name	MRS-049-	Instrument / Ser#	micrOTOF 213750.10321
Comment			

Acquisition Parameter

Source Type	ESI	Ion Polarity	Positive	Set Nebulizer	0.3 Bar
Focus	Not active			Set Dry Heater	180 ‹C
Scan Begin	50 m/z	Set Capillary	4500 V	Set Dry Gas	4.0 l/min
Scan End	1200 m/z	Set End Plate Offset	-500 V	Set Divert Valve	Waste

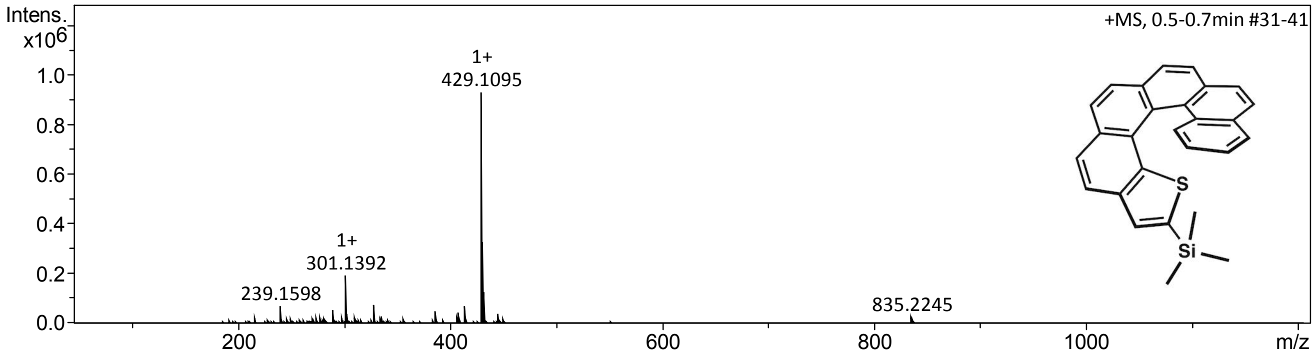

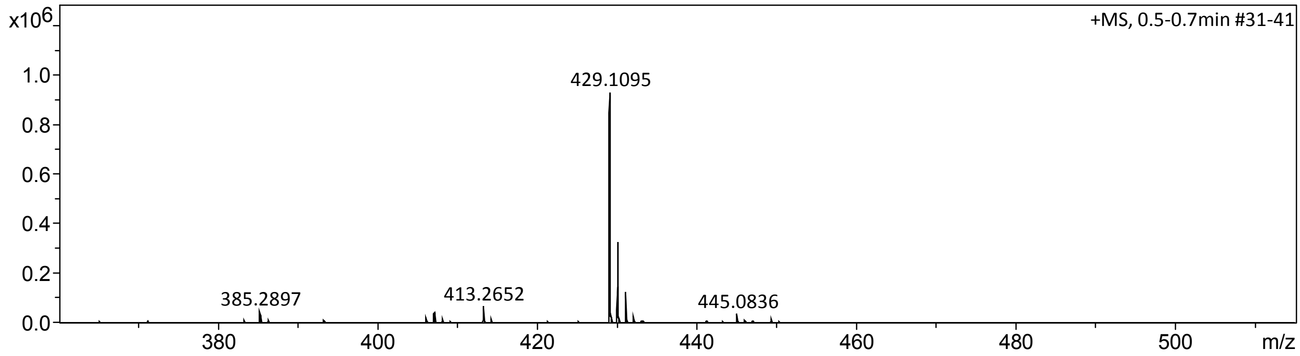

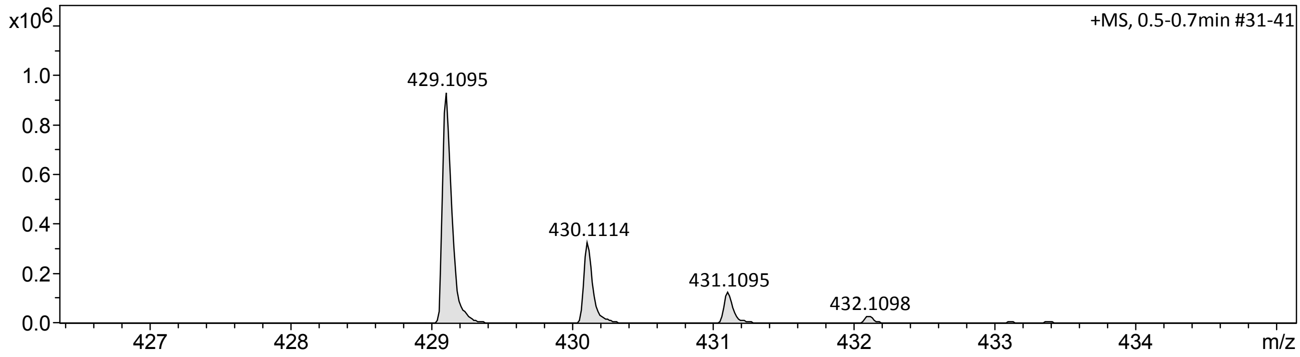

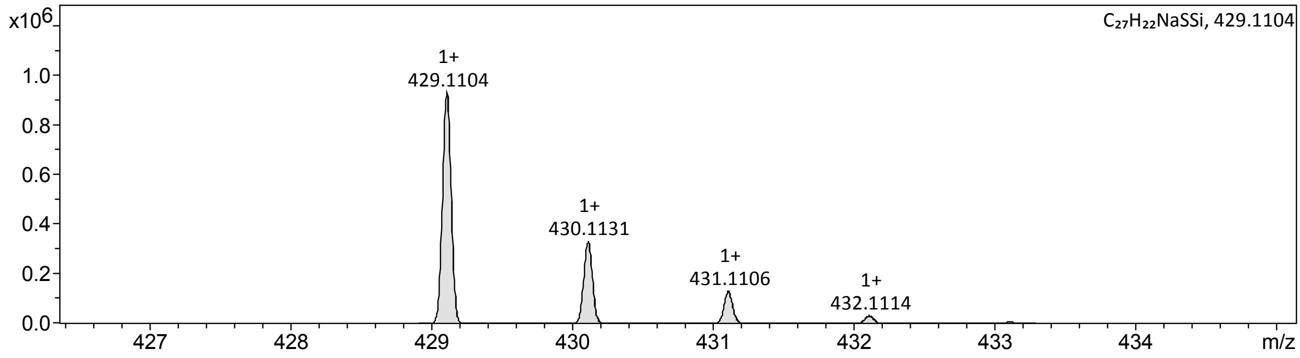

TMS-1e

TDCMAS ESI-TOF

Analysis Info

Analysis Name	D:\Data\ofcbunseki\irai\2025\aliance\250513murase\MRS-050-000001.d	Acquisition Date	2025/05/12 11:24:21
Method	check.m	Operator	BDAL@DE
Sample Name	MRS-050-	Instrument / Ser#	micrOTOF 213750.10321
Comment			

Acquisition Parameter

Source Type	ESI	Ion Polarity	Positive	Set Nebulizer	0.3 Bar
Focus	Not active			Set Dry Heater	180 ‹C
Scan Begin	50 m/z	Set Capillary	4500 V	Set Dry Gas	4.0 l/min
Scan End	1200 m/z	Set End Plate Offset	-500 V	Set Divert Valve	Waste

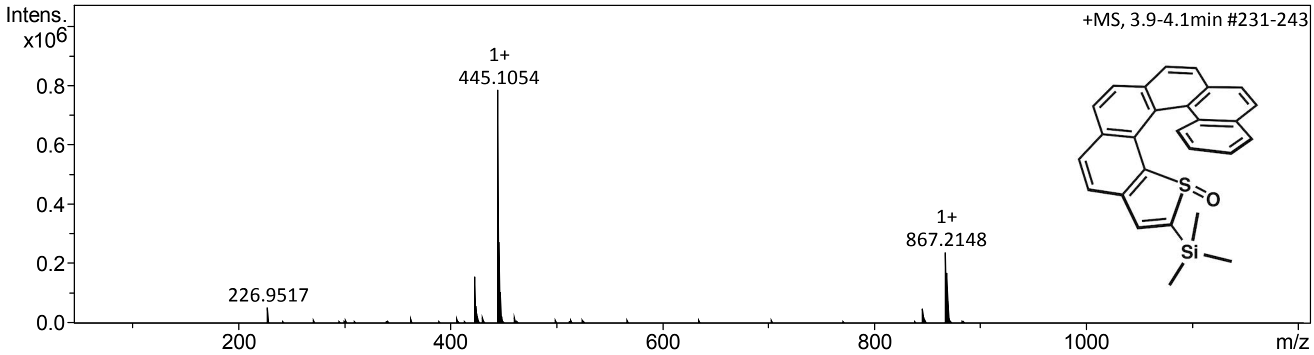

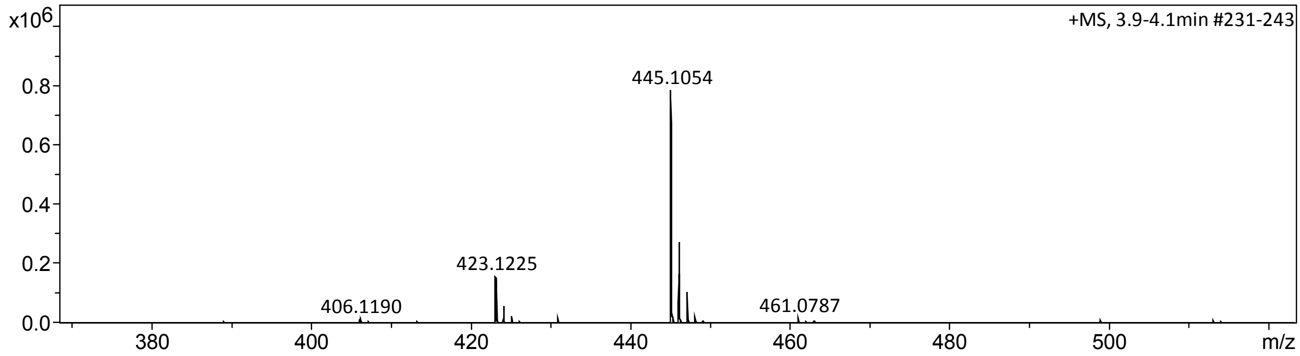

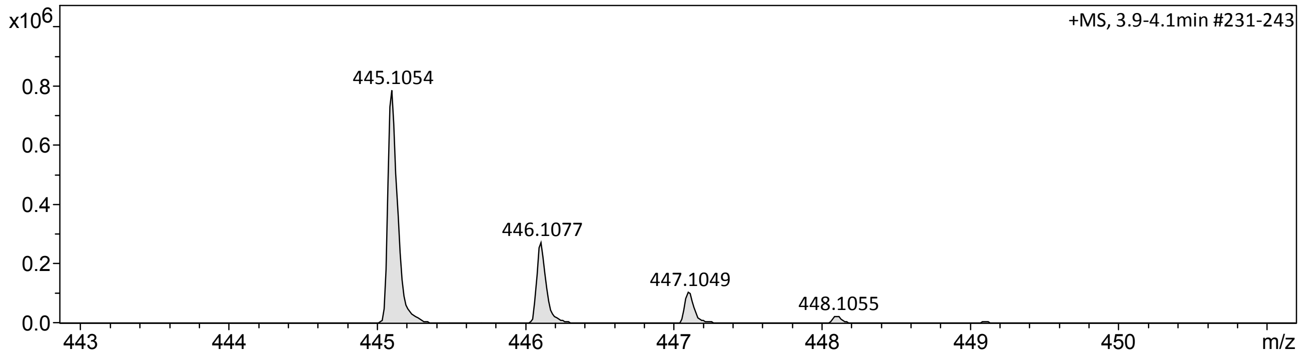

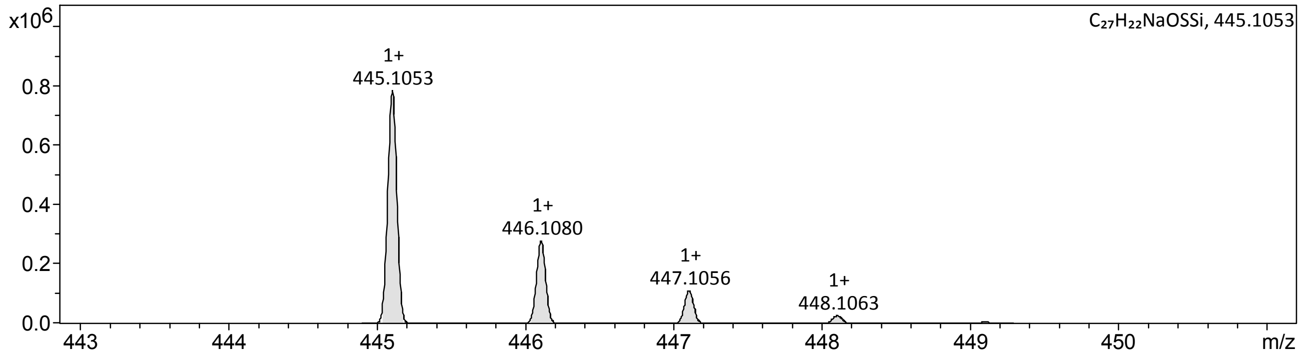

TMS-coronene

TDCMAS ESI-TOF

Analysis Info

Analysis Name	D:\Data\ofcbunseki\irai\2025\aliance\250513murase\MRS-052-APCI-000003.d	Acquisition Date	2025/05/13 10:52:51
Method	APCI_II_pos_microTOF.m	Operator	BDAL@DE
Sample Name	MRS-052-	Instrument / Ser#	micrOTOF 213750.10321
Comment			

Acquisition Parameter

Source Type	APCI	Ion Polarity	Positive	Set Nebulizer	2.0 Bar
Focus	Not active			Set Dry Heater	200 ‹C
Scan Begin	50 m/z	Set Capillary	4500 V	Set Dry Gas	3.5 l/min
Scan End	2000 m/z	Set End Plate Offset	-500 V	Set Divert Valve	Waste

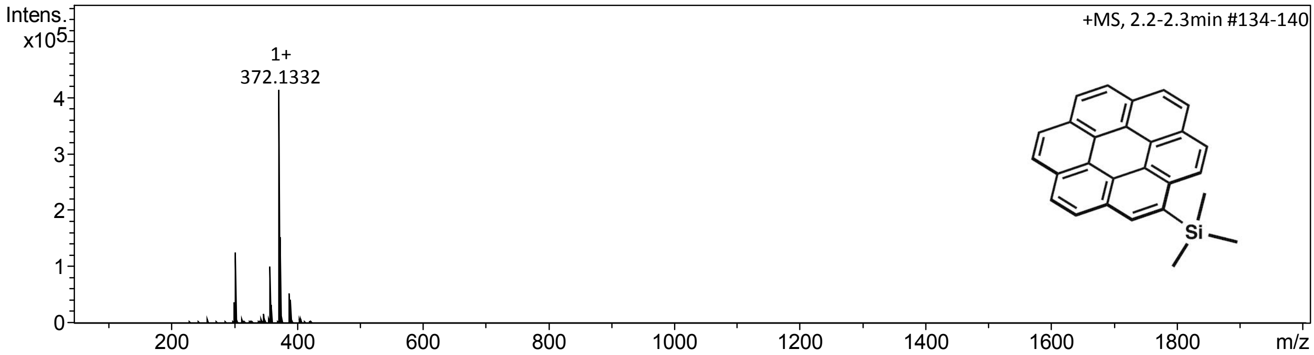

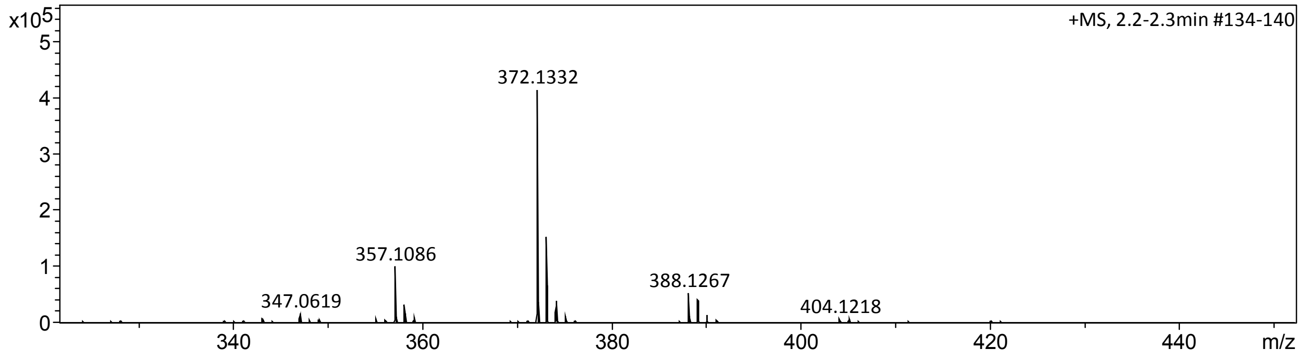

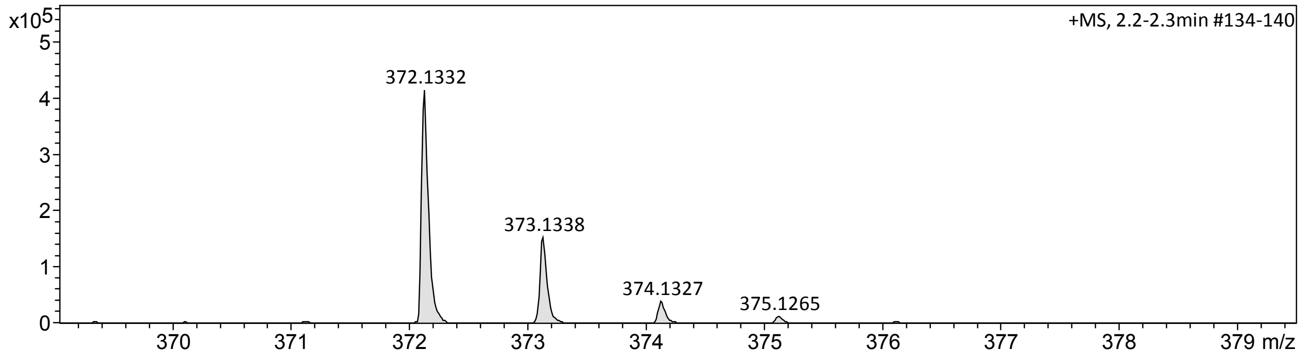

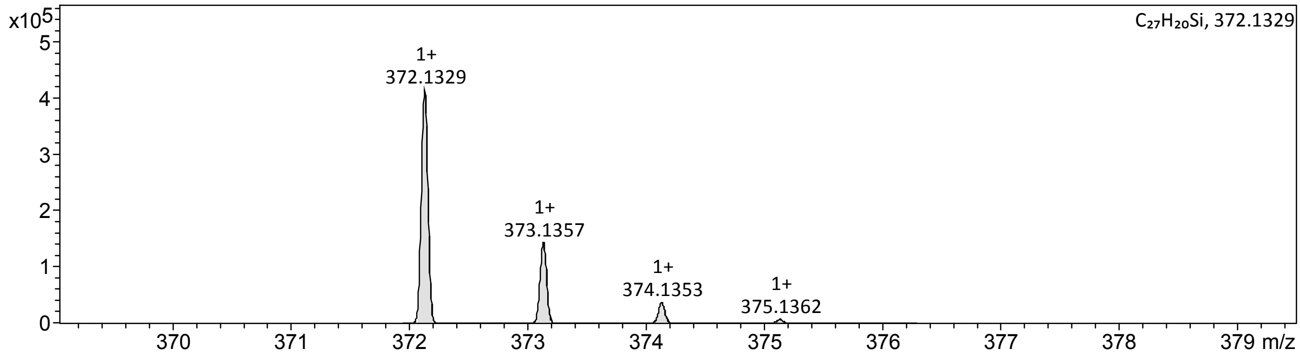

7. References

[S1] K. Seino, T. Okano, K. Oya, H. Katagiri, T. Murase, *Chem. Eur. J.* **2024**, *30*, e202402445.

[S2] *CrysAlisPro CCD, CrysAlisPro RED and ABSPACK in CrysAlisPro RED*. Oxford Diffraction Ltd, Abingdon, England, **2006**.

[S3] SHELXT: G. M. Sheldrick, *Acta Crystallogr., Sect. A* **2015**, *71*, 3–8.

[S4] SHELXL2014: G. M. Sheldrick, *Acta Crystallogr., Sect. C* **2015**, *71*, 3–8.

[S5] a) Yadokari–XG: K. Wakita, Software for crystal Structure Analyses, **2001**; b) Yadokari–XG 2009: C. Kabuto, S. Akine, T. Nemoto, E. Kwon, *J. Cryst. Soc. Jpn.* **2009**, *51*, 218–224.
